# Identification and validation of tumor microenvironment-related signature for predicting prognosis and immunotherapy response in patients with lung adenocarcinoma

**DOI:** 10.1038/s41598-023-40980-2

**Published:** 2023-08-21

**Authors:** Chunhong Li, Yixiao Yuan, Xiulin Jiang, Qiang Wang

**Affiliations:** 1 Department of Oncology, Suining Central Hospital, Suining, 629000 Sichuan China; 2 Key Laboratory of Molecular Oncology and Epigenetics, The First Affiliated Hospital of Chongqing Medical University, Chongqing, China; 3 Gastrointestinal Surgical Unit, Suining Central Hospital, Suining, 629000 Sichuan China

**Keywords:** Cancer, Computational biology and bioinformatics, Biomarkers

## Abstract

Mounting evidence has found that tumor microenvironment (TME) plays an important role in the tumor progression of lung adenocarcinoma (LUAD). However, the roles of tumor microenvironment-related genes in immunotherapy and clinical outcomes remain unclear. In this study, 6 TME-related genes (PLK1, LDHA, FURIN, FSCN1, RAB27B, and MS4A1) were identified to construct the prognostic model. The established risk scores were able to predict outcomes at 1, 3, and 5 years with greater accuracy than previously known models. Moreover, the risk score was closely associated with immune cell infiltration and the immunoregulatory genes including T cell exhaustion markers. In conclusion, the TME risk score can function as an independent prognostic biomarker and a predictor for evaluating immunotherapy response in LUAD patients, which provides recommendations for improving patients’ response to immunotherapy and promoting personalized tumor immunotherapy in the future.

Lung cancer is the most common form of cancer and is the leading cause of cancer-related deaths worldwide^1^. It poses a serious threat to human health. Lung adenocarcinoma (LUAD) is the main pathologic subtype of lung cancer. Nowadays, immunotherapy represented by anti-PD-1/PD-L1 has yielded a considerable clinical benefit for patients with various cancer types^2–5^, including lung cancer. However, most patients showed minimal or no efficacy, which is far from the clinical need^6^. Thus, it is urgently needed to identify reliable prognostic biomarkers that can predict clinical outcomes and guide targeted clinical therapy, to improve the prognosis and increase the proportion of responders to immunotherapy in LUAD.

The tumor microenvironment (TME) has attracted increasing attention due to its crucial roles in angiogenesis, metastasis, and therapeutic response^7–9^. The TME is mainly composed of immune cells (lymphocytes, dendritic cells, macrophages), stromal cancer-associated fibroblasts (CAFs), endothelial cells, extracellular matrix (ECM), and soluble signaling molecules^10–13^. TME is mainly composed of immune infiltrating cells, tumor-related fibroblasts, blood vessels, as well as extracellular matrix (ECM) and signaling molecules^7^. Notably, studies have shown that the degree and proportion of tumor-infiltrating cells contribute to the distinct prognosis of LUAD patients. Immune cells with immunosuppressive phenotypes such as myeloid-derived suppressor cells (MDSCs), tolerogenic DCs (tDCs), tumor-associated macrophages (TAMs), and regulatory T cells (T regs) accumulate in LUAD^14^. Therefore, tumor tissue gene expression profiles can reflect the relationship between TME and lung cancer patient prognosis.

In our study, we investigated the potential role of TME for lung cancer with the TCGA-LUAD and diverse GEO cohorts. Based on the above data, we established a prognostic model and a nomogram combining risk signature and clinical features according to TME-related genes. In addition, we also verified the synergistic effect of risk score and tumor immune infiltration microenvironment, tumor mutational burden (TMB), the relationship of immunotherapy responsiveness, and drug sensitivity.

## Materials and methods

### Data download and extraction

The RNA-seq (FPKM values) and somatic mutation data (MuTect2) of LUAD patients were obtained from the TCGA data portal (https://portal.gdc.cancer.gov/) and the FPKM values were transformed into TPM values. The clinicopathological characteristics of the TCGA-LUAD samples were curated from the cBioportal for Cancer Genomics database (https://www.cbioportal.org/). The TCGA-LUAD cohort consisted of 595 RNA sequencing samples, including 59 normal samples and 535 tumor samples. We removed samples without clinical follow-up information from the TCGA-LUAD cohort, resulting in 504 tumor samples. We also downloaded somatic mutation data from the TCGA database for further analysis of copy number variation (CNV). We obtained the GSE68571 cohort from the GEO database as an external validation dataset. Microarray data of GSE68571 were obtained from Affymetrix Human Full Length HuGeneFL Array, and the normalized matrix file was downloaded directly. All cases in the GSE68571 cohort contain survival information. TME-related genes were obtained from previous studies^15–17^, which, after summarizing, provided 4,061 genes (Table S1). GEO cohort (GSE50081, GSE13213, and GSE10072) using validation of the prognosis and immune role of TME-related gene in LUAD.

### Selection of differentially expressed genes (DEGs) related to TME

The R package “limma” was applied to calculate the expression of candidate genes in lung cancer and normal lung tissue. We identified the false discovery rate (FDR) < 0.05 and |log_2_Fold Change (FC)|> 1 as the threshold to screen DEGs. Log_2_FC > 1 represents gene up-regulation, and log_2_FC < −1 represents gene down-regulation in tumor tissue.

### Molecular subtype identification using a non-negative matrix factorization (NMF) Algorithm

We intended to cluster the sample with the inner feature in tumor samples and extracted biological correlation coefficients by NMF algorithm, an R package “NMF” was used to take the above action. We selected an optimum k value in 2 to 10 in consideration of stability and clustering performance.

### Definition and comparison of tumor-infiltrating immune cells

By using Microenvironment Cell Populations, we calculated the absolute abundance of immune cells and stromal cells. By contrasting the scores between clusters, it was possible to identify the infiltrating cells.

### Construction and validation of TME-related genes prognosis features

To screen out the DEGs that may be related to prognosis, we performed Cox regression with R packages “survival” and “survminer” to obtain candidate genes. The Lasso algorithm was executed to construct a prognostic model by the “glmnet” R package. We determined the risk score for each sample using the formula "RiskScore = eSi(Coefi·Expi)" and used the median risk score as the cut-off to categorize each sample as high- or low-risk groups. The training set was made up of TCGA data, and the testing set was made up of GEO data. Receiver operating curve (ROC) analysis was used to confirm that the prognostic model was stable. To establish the risk score as a significant prognostic factor, a univariate and multivariate Cox regression was conducted. The "heatmap" package showed the relationship between risk scores and clinical variations. The risk score of each sample was calculated with the optimized genes based on the following formula:

$$ {\text{Risk}}\,{\text{score}} = \sum {\text{ni}} = {\text{1Coefi}}*{\text{XiRisk}}\,{\text{score}} = \sum {\text{i}} = {\text{1nCoefi}}*{\text{Xi}}, $$

where CoefiCoefi is the risk coefficient of each factor calculated by the multivariate Cox model, and XiXi is the expression level of each TME-related genes.

### Gene set enrichment analysis (GSEA)

GSEA 4.1 software was used to investigate the function annotation in high- and low-risk groups using the c2.cp. Kegg.v7.4.symbols collection. Results were deemed statistically significant when *p* < 0.05. The top eight outcomes were chosen for visualization.

### Establishment and verification of the nomogram

R program "rms" was used to finish the establishment, and "regplot" was used to complete the visualization. The model was built taking into account nomogram risk scores and clinical features. We offer a measurable tool to forecast overall survival at 1, 3, and 5 years. The calibration curves were created to evaluate the effectiveness of the nomogram.

### Correlation between TMB and risk scores

We took information about somatic mutations from the TCGA-LUAD cohort. We counted the somatic non-synonymous point mutations in each sample using the "maftools" R program.

### Correlation of risk score with tumor immune infiltration microenvironment (TIME) characterization

We used 7 methods, including XCELL, TIMER, QUANTISEQ, MCPcounter, EPIC, CIBERSORT, and CIBERSORT-ABS, to assess immune cell infiltration in the tumor microenvironment. The "ggpubr" package was used to visualize the results above.

### Gene set variation analysis (GSVA)

We downloaded two collections from the Molecular Signatures Database: c7.immunesigdb HALLMARK and c2.cp. Kegg.v7.4. Additionally, we downloaded 47 genes related to immune checkpoint blockades. We calculated the standardized GSVA of each gene set in each sample. As a result, we were able to determine each gene set's relative pathway activity, immunological hallmarks, and immune checkpoints.

### Anticancer drug sensitivity prediction

In clinical settings, the two main treatments for lung cancer are chemotherapy and immunotherapy. To compare the half-maximal inhibitory concentration (IC50) of LUAD samples between high- and low-risk groups. The data of the package "pRRophetic" is based on TCGA gene expression profiles and cell line expression profiles from Genomics of Cancer Drug Sensitivity. One type of treatment for advanced lung cancer is immune checkpoint inhibitors. We used the immunogen score (IPS), which uses the expression of genes related to immune checkpoint blockade [data from Genecards database(https://www.genecards.org/)] to assess the immunogenicity of a tumor in high- and low-risk populations.

### Cell culture

BEAS-2B cell line was purchased from ATCC, and cultured in BEGM media (Lonza, CC-3170). Lung cancer cell lines, including A549 and H1299, were purchased from Cobioer, China with STR document, and were cultured in RPMI-1640 medium (Corning) supplemented with 10% fetal bovine serum (FBS) and 1% penicillin/streptomycin. HEK-293 T cell line was purchased from ATCC and cultured in a DMEM medium (Corning).

### Real-time RT-PCR assay

For Real-time RT-PCR assay, indicated cells were lysed by RNAiso Plus (Takara Bio). Total RNAs were extracted according to the manufacturer’s protocol and then reverse-transcribed using an RT reagent Kit. Real-time PCR was performed by FastStart Universal SYBR Green Master Mix using an Applied Biosystems 7500 machine.

### Statistical analysis

The Kruskal–Wallis test was used to compare more than two groups, while the Wilcoxon test was used to compare two groups. It was decided to evaluate the survival curves using the Kaplan–Meier log-rank test. The chi-square test was performed to examine the relationships between risk groupings and somatic mutational burden, and Spearman analysis was utilized to determine correlation coefficients. The CIBERSORT algorithm was used to further examine the results (*p* < 0.05). Statistics were regarded as significant when a two-sided *p* < 0.05 was reached. Utilizing the software R, all statistical analyses were completed (version 4.1.1).

## Results

### Identification of DEGs between normal lung and LUAD tissues

**Figure 1:**
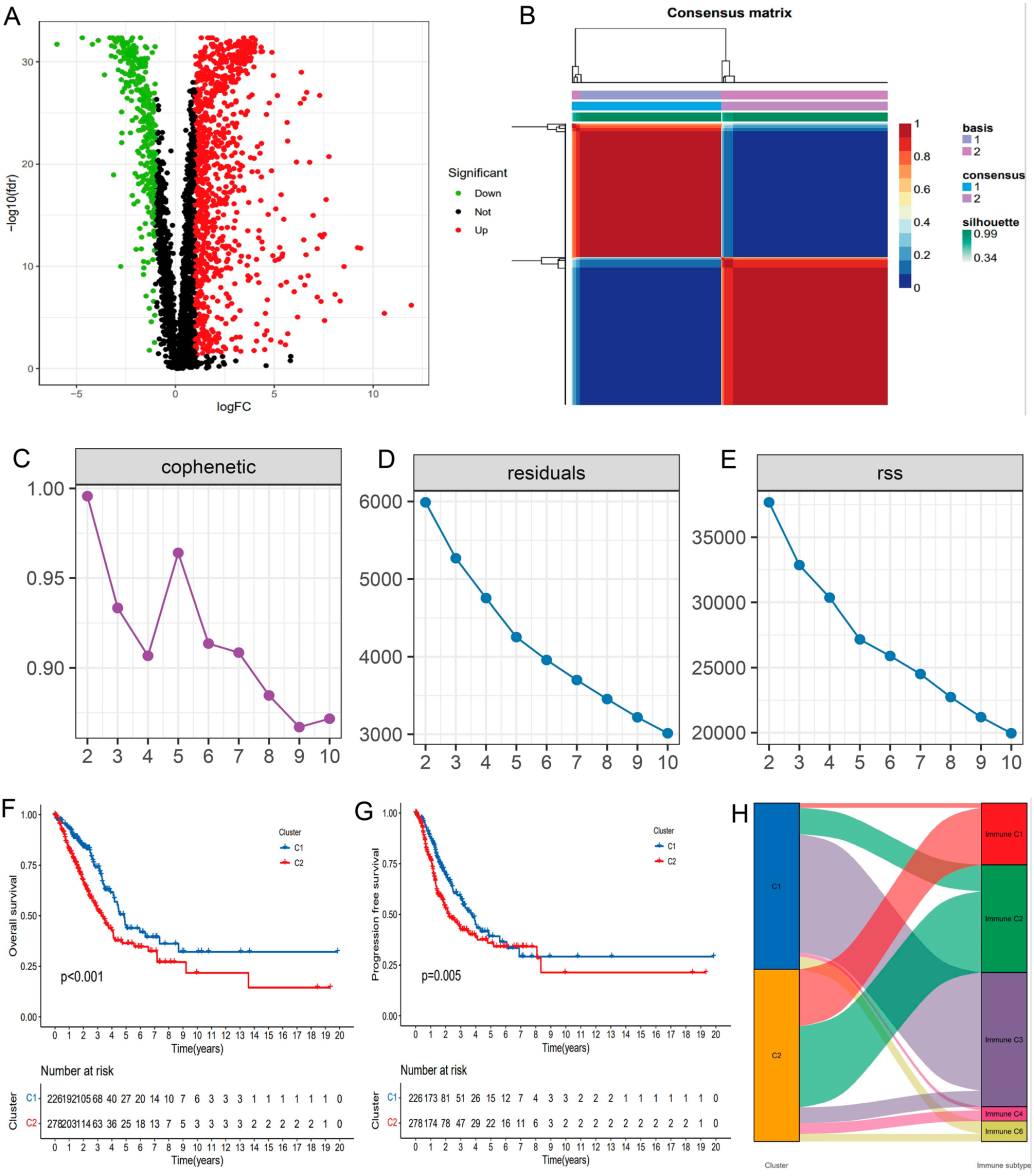
Comparison of the two clusters 1 and 2 (C1 and C2) in the TCGA-LUAD cohort. (**A**) Volcano map of the TCGA database differentially expressed genes in LUAD. (**B**) NMF algorithm-based clustering of the consensus map. (**C**,**D**) The cophenetic correlation coefficient is a measure that can be used to determine how stable the cluster is that was produced using NMF. (**E**) RSS reflects the model's performance in terms of clustering. (**F**) There were statistically significant differences between C1 and C2 in terms of overall survival. (**G**) The progression-free survival (PFS) demonstrated a significant difference between stages C1 and C2 of the disease. (H) A plot of the data from the alluvial deposits shows what percentage of C1 and C2 are present in each of the several molecular subtypes.

The flow chart describing our study is shown in Supplementary Fig. S1. From the data obtained from the TCGA-LUAD, we compared the levels of gene expression in 59 normal tissues with those in 504 malignant tissues. A total of 203 genes associated with TME were analyzed and compared. The batch effect was removed from the data of the TCGA database, and the data were normalized. There were 1251 TME-associated genes (FDR < 0.05 and |log2FC|> 1) differentiated in expression between LUAD and normal lung tissues (Table S2). Among the TME-related DEGs, 920 genes were found to be significantly up-regulated, while 331 genes were found to be significantly down-regulated. The top 50 genes in terms of differential expression that were either up-regulated or down-regulated were plotted as volcanoes (Fig. 1A).

### NMF algorithm-based molecular subtypes of OS-related genes

**Figure 2:**
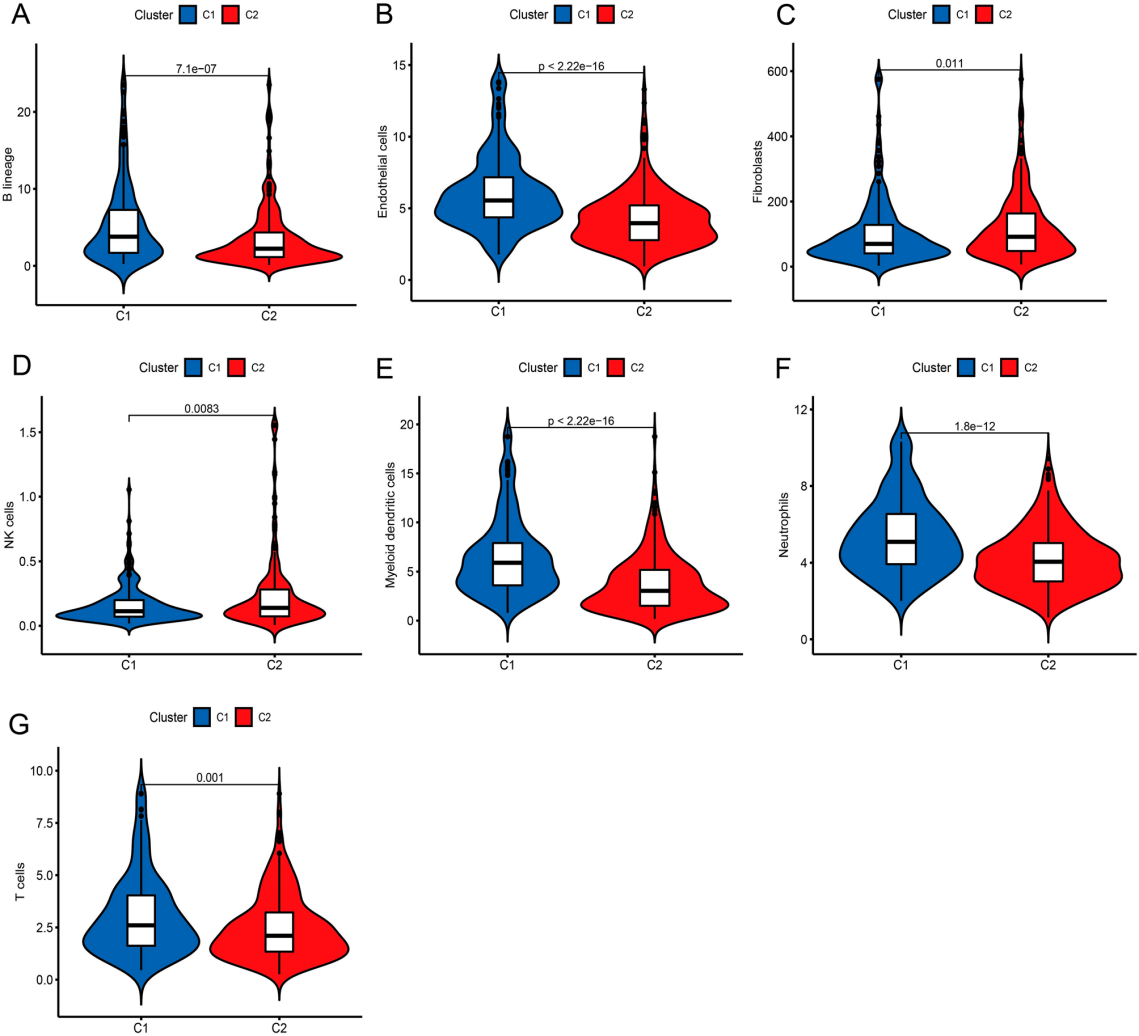
Substantial variations in immune cells of the tumor microenvironment (TME). (**A**–**G**) Numerous TME stromal cells displayed considerable differences.

Covariance and RSS were used to develop C1 and C2, and k = 2 was determined to be the optimal value for clustering based on its stability and performance (Fig. 1B–E). According to the findings of the Kaplan–Meier curve, samples that fell into the C1 cluster enjoyed longer OS and PFS than those samples that fell into the C2 cluster (Fig. 1F–H). There were significant differences between C1 and C2 in immune and stromal scores (Fig. 2A–G). The above results showed that OS-related genes can be clustered, and these clusters were related to a variety of immune cell clusters.

### Developing and validating a prognostic prediction model for OS-related genes

**Figure 3:**
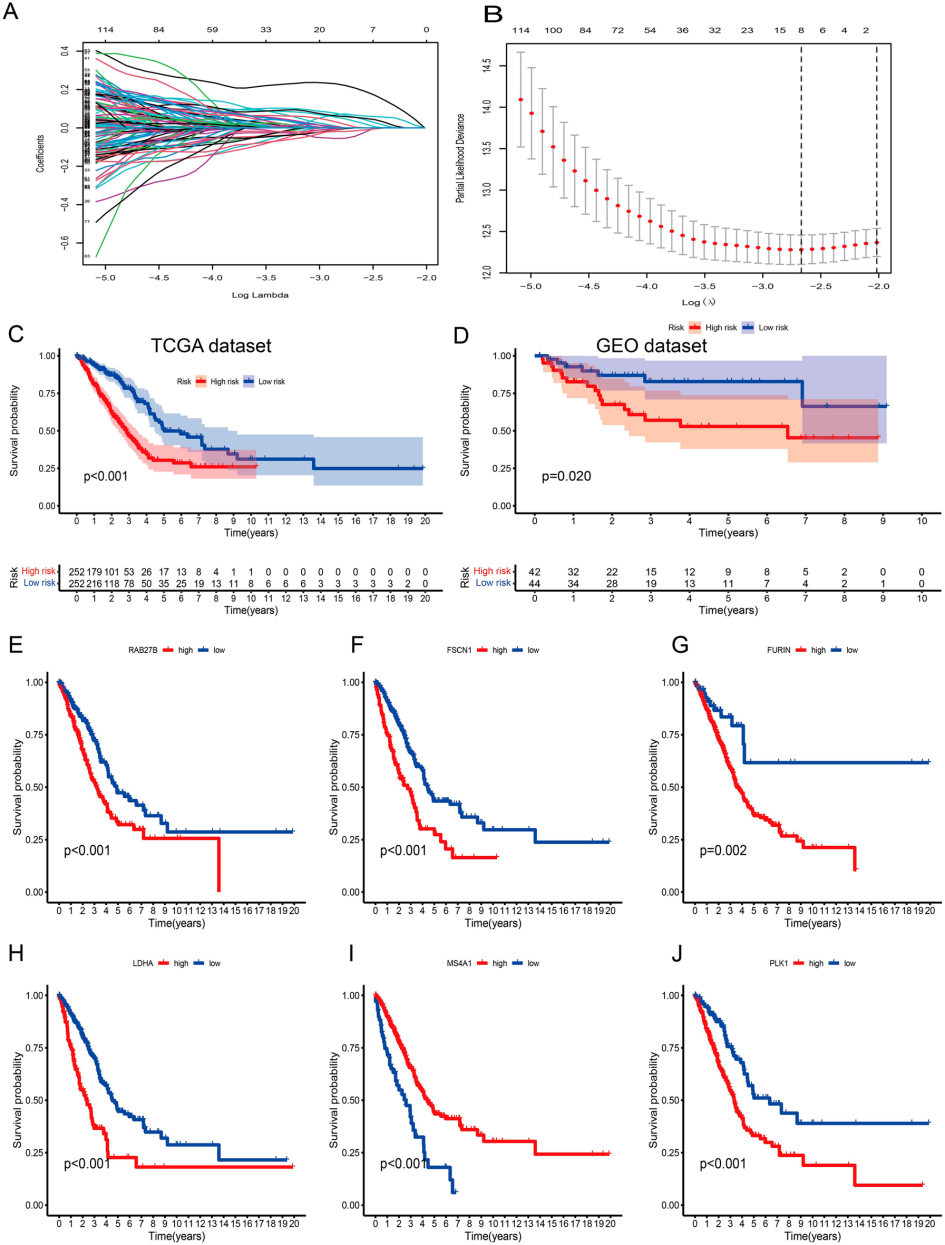
Construction and validation of the TME-related 6-gene risk score (RS). (**A**) Profiles of 203 candidate genes' LASSO coefficients. The tenfold cross-validation value is marked by a vertical line. (**B**) A lasso regression parameter adjustment using a ten-fold cross-validation. Vertical lines are formed from the best data when the minimal criterion and the one standard error criterion are both met. The vertical lines on the left side of the image show the four genes that have not yet been found. (**C**,**D**) A Kaplan–Meier curve analysis was performed on the TCGA and GEO databases to demonstrate the difference in overall survival between high-risk and low-risk groups. (**E**–**J**) A Kaplan–Meier curve analysis illustrating the difference in overall survival between patients with high and low expression of PLK1, LDHA, FURIN, FSCN1, RAB27B, and MS4A1 in lung cancer.

**Figure 4:**
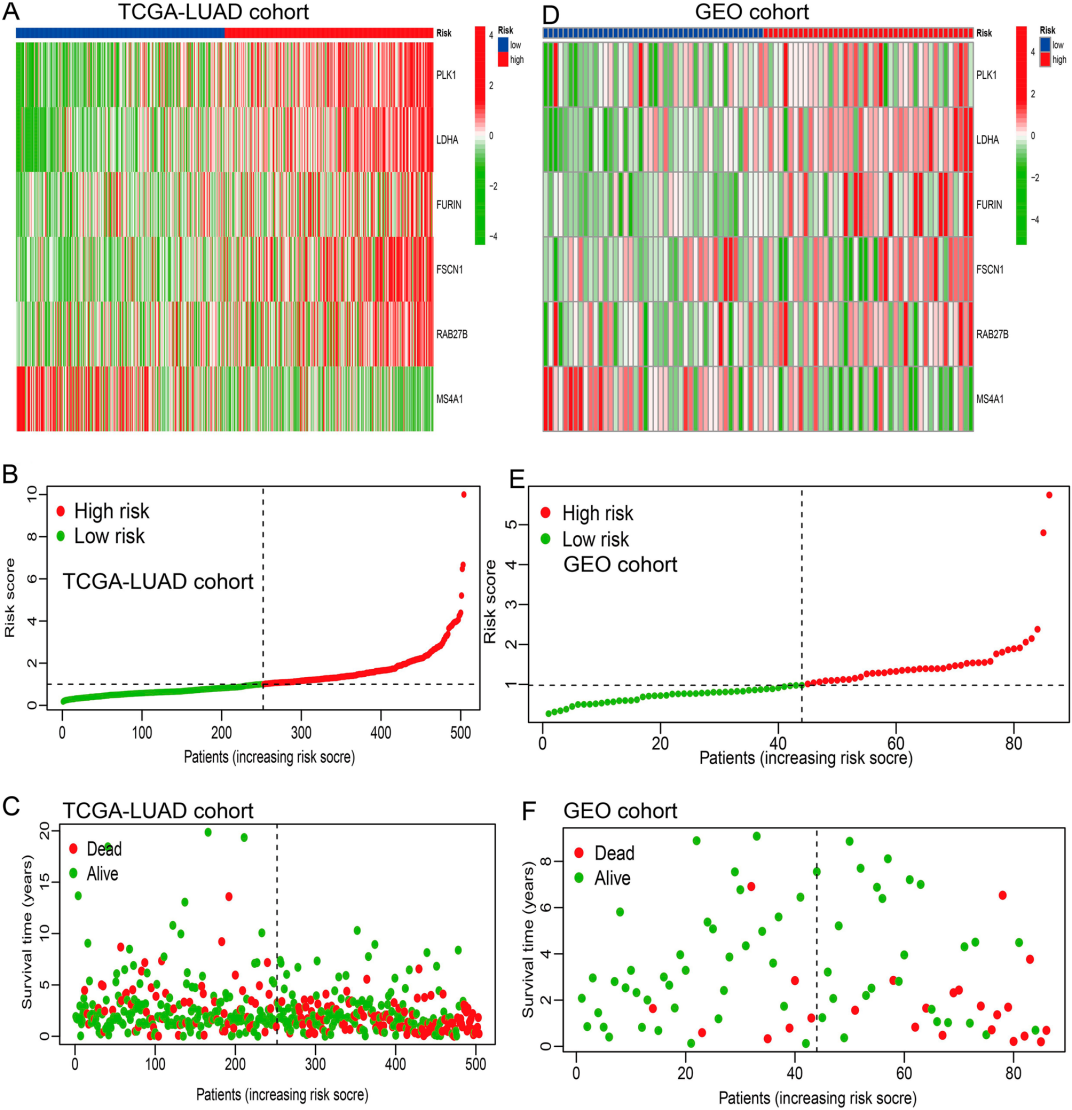
Evaluation and validation of the prediction value of the six-gene risk signature by the GEO dataset. (**A**) Confirmation of the predictive value of risk scores applied to the TCGA cohort. (**D**) The prognostic risk ratings of the GEO cohort were checked and found to be accurate. (**B**) The survival rate and urination of TCGA cohort patients who were diagnosed with LUAD. (**E**) The survival rate and overall duration of patients diagnosed with LUAD who were part of the GEO cohort. (**C**) The distribution of polygenic model risk scores among the members of the TCGA cohort. (**F**) The distribution of polygenic model risk scores among individuals in the GEO cohort.

In the expression matrix of the whole TCGA-LUAD samples with 1251 genes, 203 prognostic TME-related genes were obtained by univariate Cox analysis (Table S3). We applied LASSO and Cox regression analyses to further narrow the range of prognostic genes, and finally, constructed a TME-related gene signature involving 6 genes in the training cohort. The results of the LASSO analysis were shown in Fig. 3A,B and Table 1. The risk score for each patient was calculated as follows: risk score = (PLK1 * 0.1835) + (LDHA * 0.38) + (FURIN * 0.17) + (FSCN1 * 0.160) + (RAB27B * 0.19) + (MS4A1 * −0.14). The Clinical Proteomic Tumor Analysis Consortium (CPTAC) database was used to explore protein expression levels in LUAD samples. We extracted IHC from the HPA database. The results showed PLK1, LDHA, FURIN, FSCN1, and RAB27B were up-regulated in lung cancer tissues, and MS4A1 was down-regulated in lung cancer tissues (Supplementary Fig. S2A,B). Furthermore, we also found that PLK1, LDHA, FURIN, FSCN1, and RAB27B were increased in NSCLC cancerous cell lines compared with that in normal human bronchial epithelium cell line BEAS-2B, MS4A1 was down-regulated in lung cancer cells lines (Supplementary Fig. S2C,D). Using the median risk score in the training cohort as the cutoff point, the patients were divided into low-risk and high-risk groups. KM survival analysis indicated that the low-risk group had better overall survival than the high-risk group (P < 0.001, Fig. 3C,D). There was a significant difference in OS between the two patient groups that had high and low expression of the six genes (Fig. 3E–J). Figure 4A–C show the expression patterns of the six genes in the TCGA-LUAD cohort, the distribution of sample survival status, and corresponding risk scores. Both internal validations with the TCGA-LUAD cohort and external validation with the GSE68571 cohort (Fig. 4D–F) demonstrated a stable and robust prognostic value for this risk prognostic feature (Fig. 4D–F). We further assess the predictive value of the TME-related gene signature in the external test cohort. The results from the above data (GSE13213/ GSE50081 and GSE10072 cohort) shared the same trend in survival, respectively (Supplementary Fig. S3A).

**Table 1:** A six-gene signature in lung adenocarcinoma microenvironment.

Gene	HR	HR.95L	HR.95H	*p* Value
PLK1	1.436513	1.234352	1.671783	2.86E−06
LDHA	1.788229	1.441504	2.218351	1.26E−07
FURIN	1.227379	1.091252	1.380487	0.000636
FSCN1	1.286063	1.146587	1.442505	1.74E−05
RAB27B	1.208766	1.062161	1.375607	0.004051
MS4A1	0.818131	0.731369	0.915186	0.000449

### Construction of prognostic nomogram

**Figure 5:**
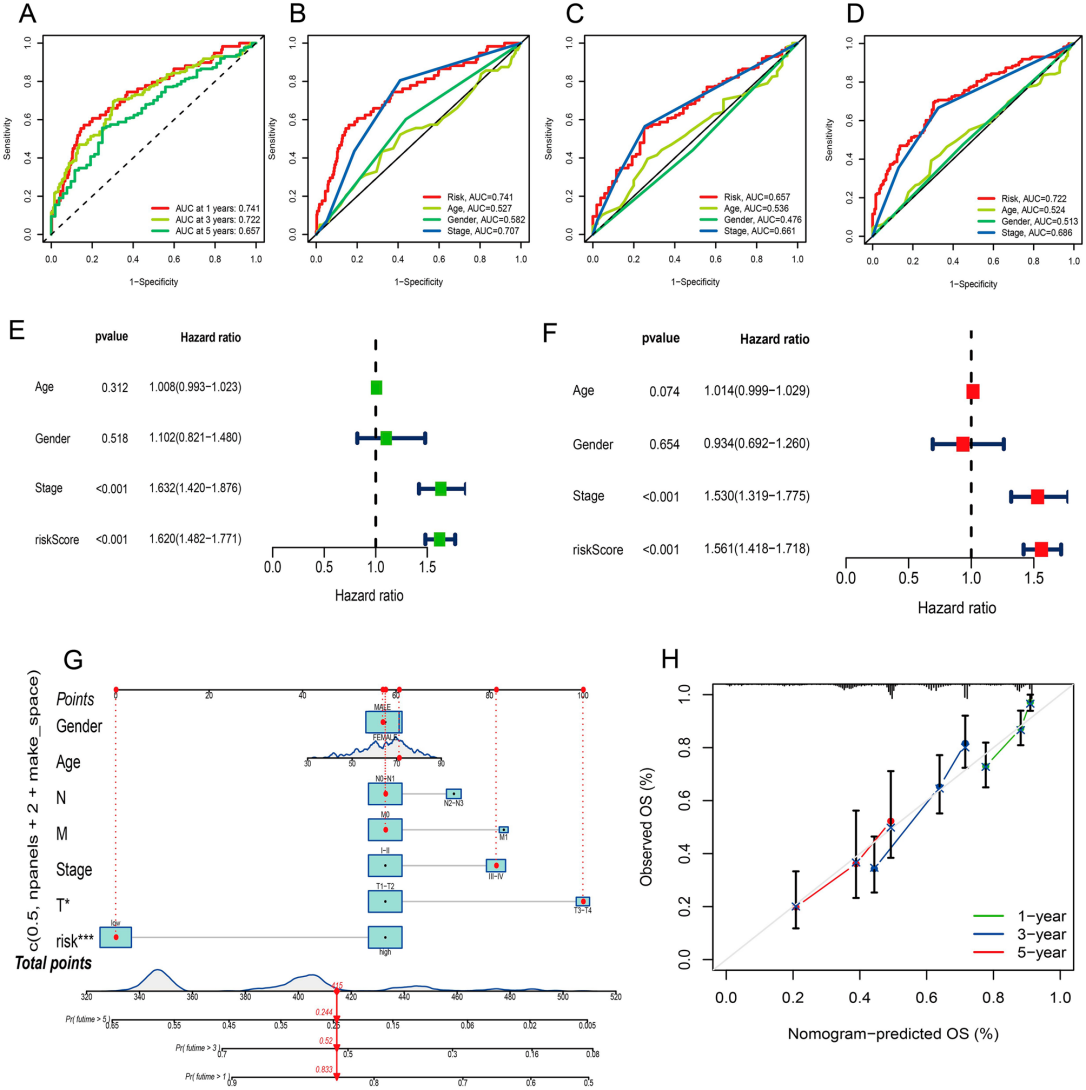
Validation of risk signatures prognostic effectiveness. (**A**–**D**) A ROC analysis was carried out so that the prognostic value of the prognostic characteristics could be determined. The area under the curve of the risk score, together with other clinical indicators used to predict overall survival at 1, 3, and 5 years (**E**) The results of running a Cox regression with just one variable to analyze overall survival. (**F**) The conclusions were drawn from running a multivariate Cox regression on overall survival data. (G) In LUAD patients, a nomogram was utilized to predict survival. (**H**) Curves for the 1-, 3-, and 5-year nomogram calibration.

The area under the ROC curve was 0.741 for a 1-year overall survival rate, 0.722 for a 3-year overall survival rate, and 0.657 for a 5-year overall survival rate (Fig. 5A). We integrated gender, tumor grade, T, N, and M stages for AUC analysis of 1-year (Fig. 5B), 3-year (Fig. 5C), and 5-year (Fig. 5D) OS to further confirm that risk score can be a prognostic signal among numerous clinicopathological characteristics. This was done to further confirm that the risk score can be a prognostic signal among numerous clinicopathological characteristics. When compared to the AUC value the risk score and clinicopathological features were all less significant. There was a significant difference between the univariate and multivariate Cox regression results for overall survival, with the difference being significant (Fig. 5E,F). To evaluate the significance of model prediction, we developed a predictive nomogram consisting of risk score and clinicopathological features (Fig. 5G). Constructing the calibration curve allowed for the evaluation of the nomogram's predictive performance, which may result in an underestimate of the 5-year survival rate (Fig. 5H).

### Functional analysis of OS-related genes

**Figure 6:**
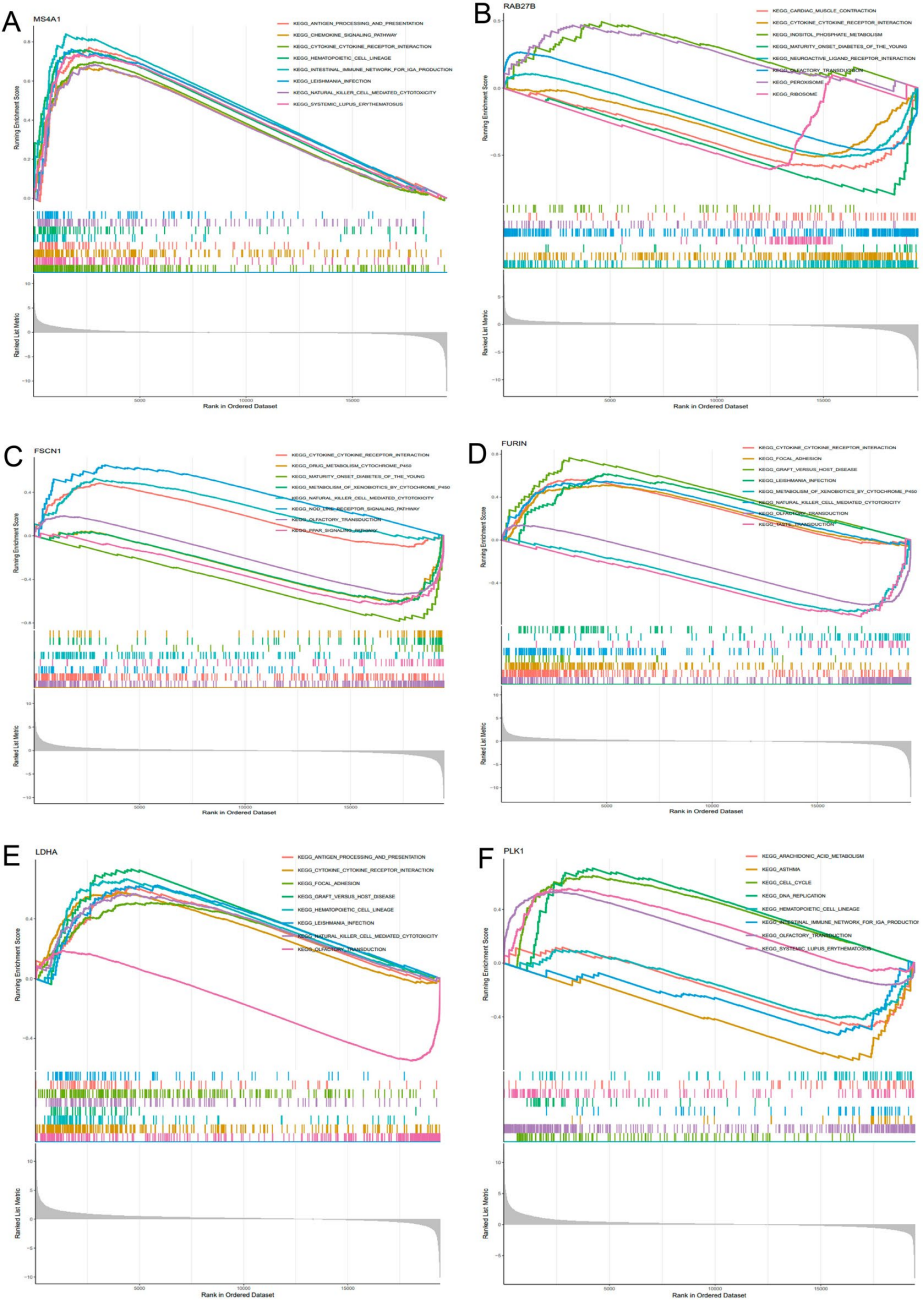
GSEA results for samples that have high or low expression of 9 core genes. (**A**–**F**) An enriched gene set was compiled with KEGG using data from samples that had high PLK1, LDHA, FURIN, FSCN1, RAB27B, and MS4A1 expression.

The median expression of the hub genes was chosen as the cut-off value, and all samples were divided into low-expression and high-expression groups based on where they fell on that scale. GSEA later uncovered a functional enrichment of genes. According to the findings of an analysis conducted by KEGG, a high level of MS4A1 expression is connected to certain signaling pathways, such as the cytokine-cytokine receptor interaction circuit and the chemokine signaling network. There was a correlation between the increased expression of LDHA and the cytosolic DNA sensing pathway, in addition to other signaling pathways. Two distinct signaling routes were found to be connected to the elevated levels of PLK1 expression. These pathways were the chemokine signaling pathway and the system that involves the interaction of cytokines with their respective receptors. The enhanced expression of RAB27B was found to be related to the activation of certain signaling pathways, including cytochrome P450-mediated xenobiotic metabolism and retinol metabolism (Fig. 6A–F).

### Association of risk characteristics with clinicopathological variables

**Figure 7:**
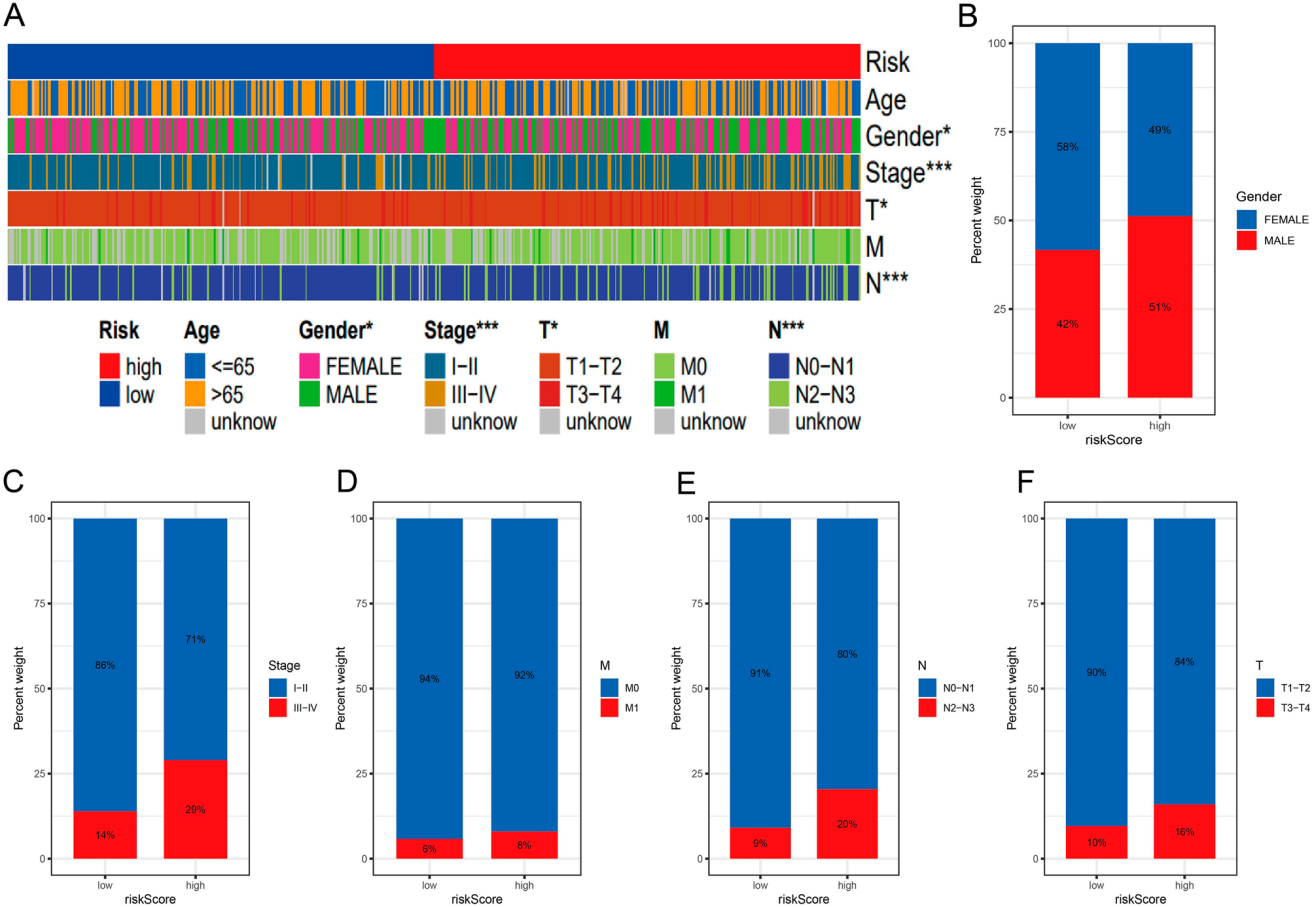
The significance of predictive risk variables in clinical practice. (**A**) A heatmap illustrating the distribution of the clinical characteristics and risk ratings associated with each sample. Prevalence of clinically different subgroups based on LRG or HRG. (**B**) Gender, (**C**) Tumor stage, (**D**) Stage M, (**E**) Stage N and (**F**) Stage T.

To explore the correlation between risk and clinicopathological variables, we visualized a plot based on clinicopathological features (Fig. 7A–F). There was a difference in gender, stage, T stage, M stage, and N stage. The results were consistent with clinical practice.

### Association of risk signature with TMB

**Figure 8:**
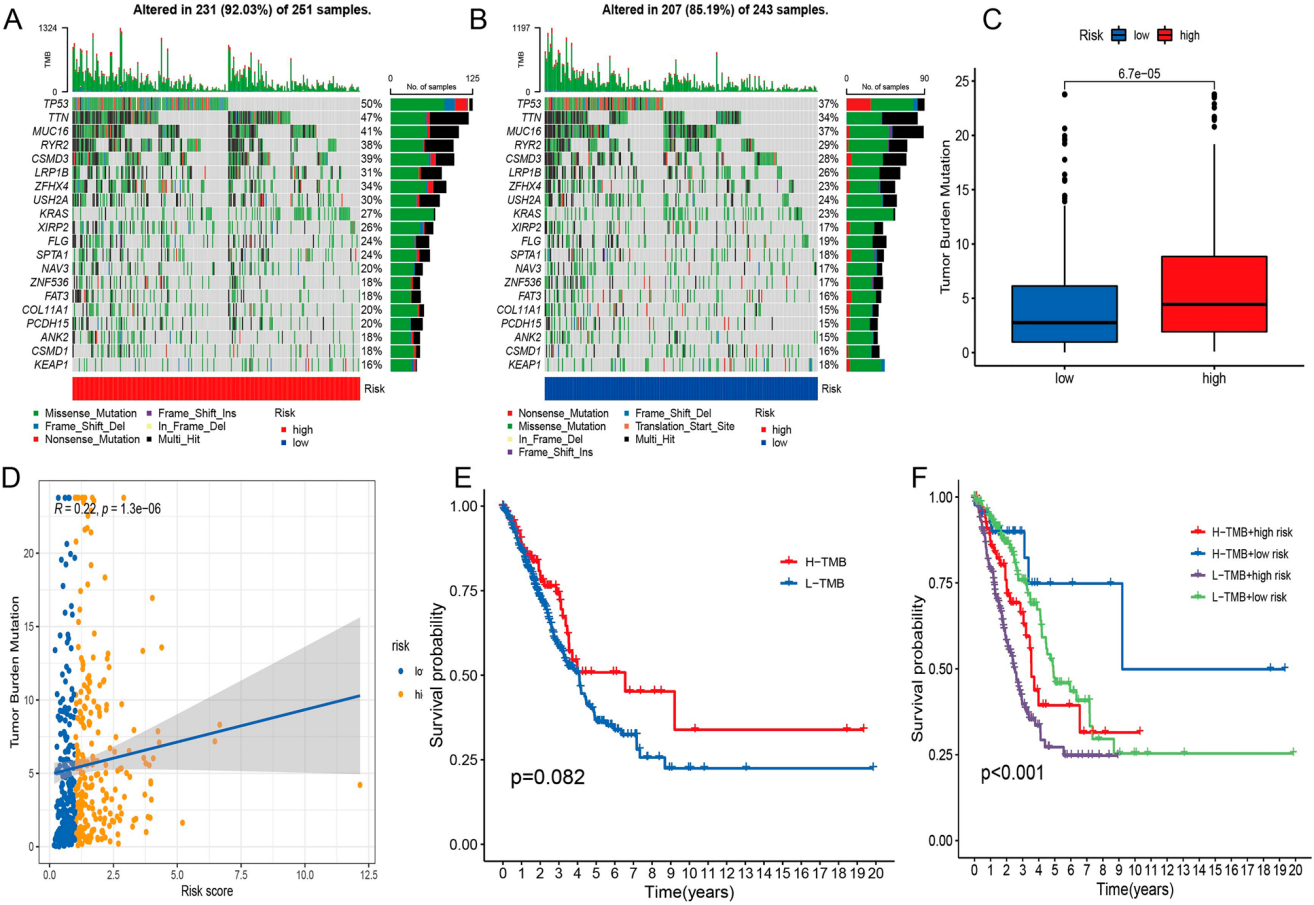
Correlation between risk score and TMB. High-risk score (**A**) and low-risk score were used to create the oncoPrint (**B**). (**C**,**D**)Correlation between risk score and TMB. (**E**) The Kaplan–Meier curves for groups with high and low TMB. (**F**) Patient stratification using the Kaplan–Meier curve based on TMB and risk signature.

To study the connection between risk score and gene mutations, we first determined how gene changes were distributed between high-risk (HRG) and low-risk (LRG) groups, and then we visualized the top 20 gene waterfalls that had the most common somatic mutations (Fig. 8A,B). Significantly, mutated gene mutation profiles revealed that TP53 had a higher rate of somatic mutations gene (SMG) in the high-risk group (50% vs 37%), whereas KEAP1 had a higher rate of somatic mutation in the low-risk group (18% vs 16%). This difference was because TP53 is associated with a higher risk of developing cancer overall. We found that HRG had a higher tumor mutational burden (TMB) (Fig. 8C). The Risk scores (RS) and TMB of each sample are shown in Fig. 8D. According to the findings of the Kaplan–Meier analysis, overall survival of high and low levels of TMB does not no any difference ( Fig. 8E). Patients were categorized into the following four categories according to their TMB risk. The plot implied that TMB did not impair the risk score's prognostic ability (*p* < 0.001, Fig. 8F). The aforesaid results proved that risk scores can predict the effect of immunotherapy and were considered a prognostic indicator.

### Risk signature in TIME context

**Figure 9:**
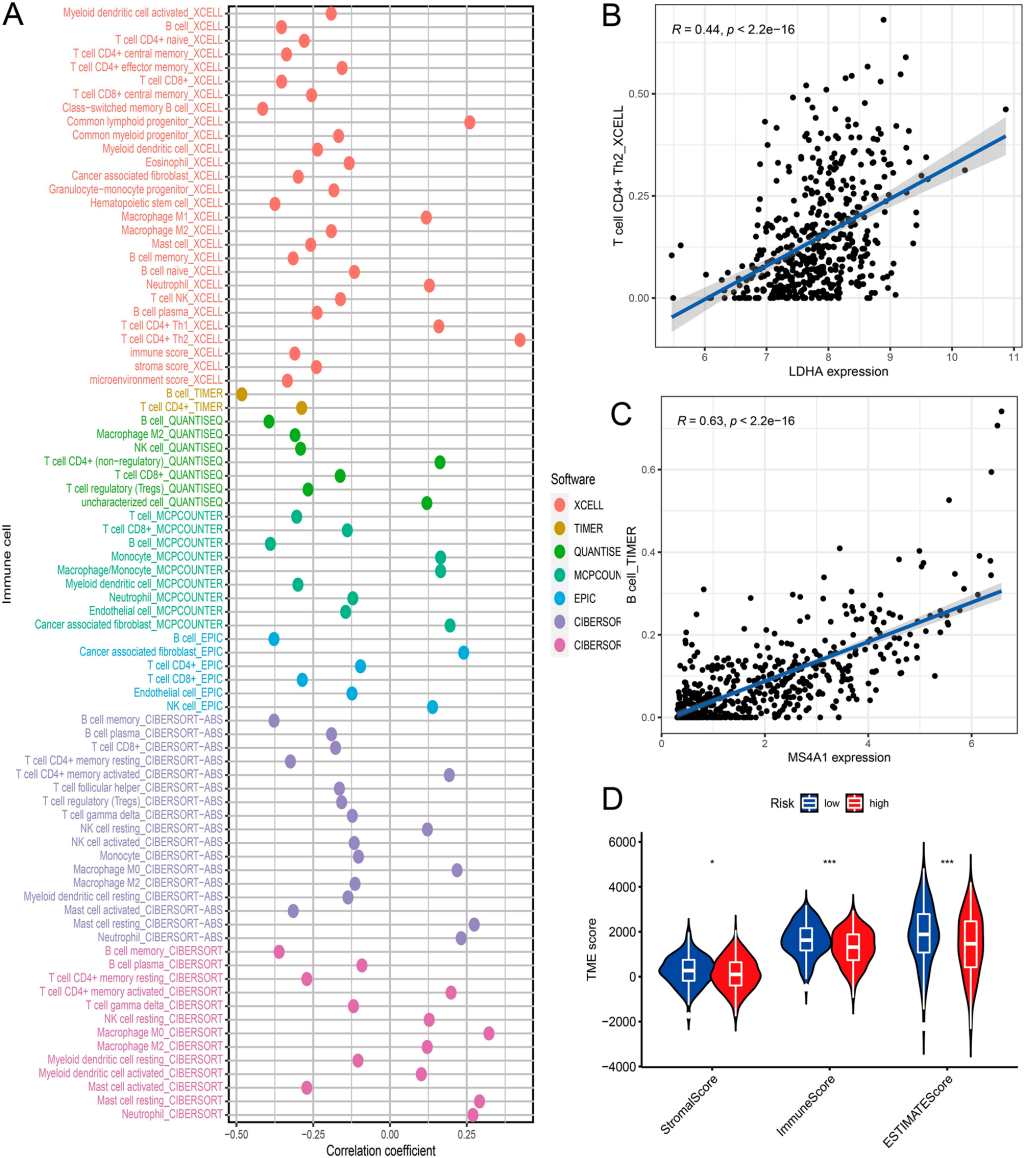
Tumor-infiltrating cells abundance estimation. The results of the Spearman correlation analysis demonstrated a greater link between patients in the (**A**) high-risk category and tumor-infiltrating immune cells. (**B**,**C**) The correlation between the score of the microenvironment and the expression of LDHA and MS4A1. (**D**) A comparison of the TME score for groups that are high risk with groups that are low risk.

We used 7 methods to investigate the connection between risk score and immune infiltration as well as stromal cells to gain a deeper understanding of the connection between risk score and TIME (Fig. 9A). The XCELL study found that the microenvironment score increased in direct proportion to the level of LDHA and MS4A1 expression (Fig. 9B,C). A strong upward trend in high-risk scores was seen when utilizing the ESTIMATE method, and there were significant differences between high-risk and low-risk groups in the ESTIMATE scores, immunological scores, and stromal scores (Fig. 9D).

### Biological function and signal pathway enrichment analysis

**Figure 10:**
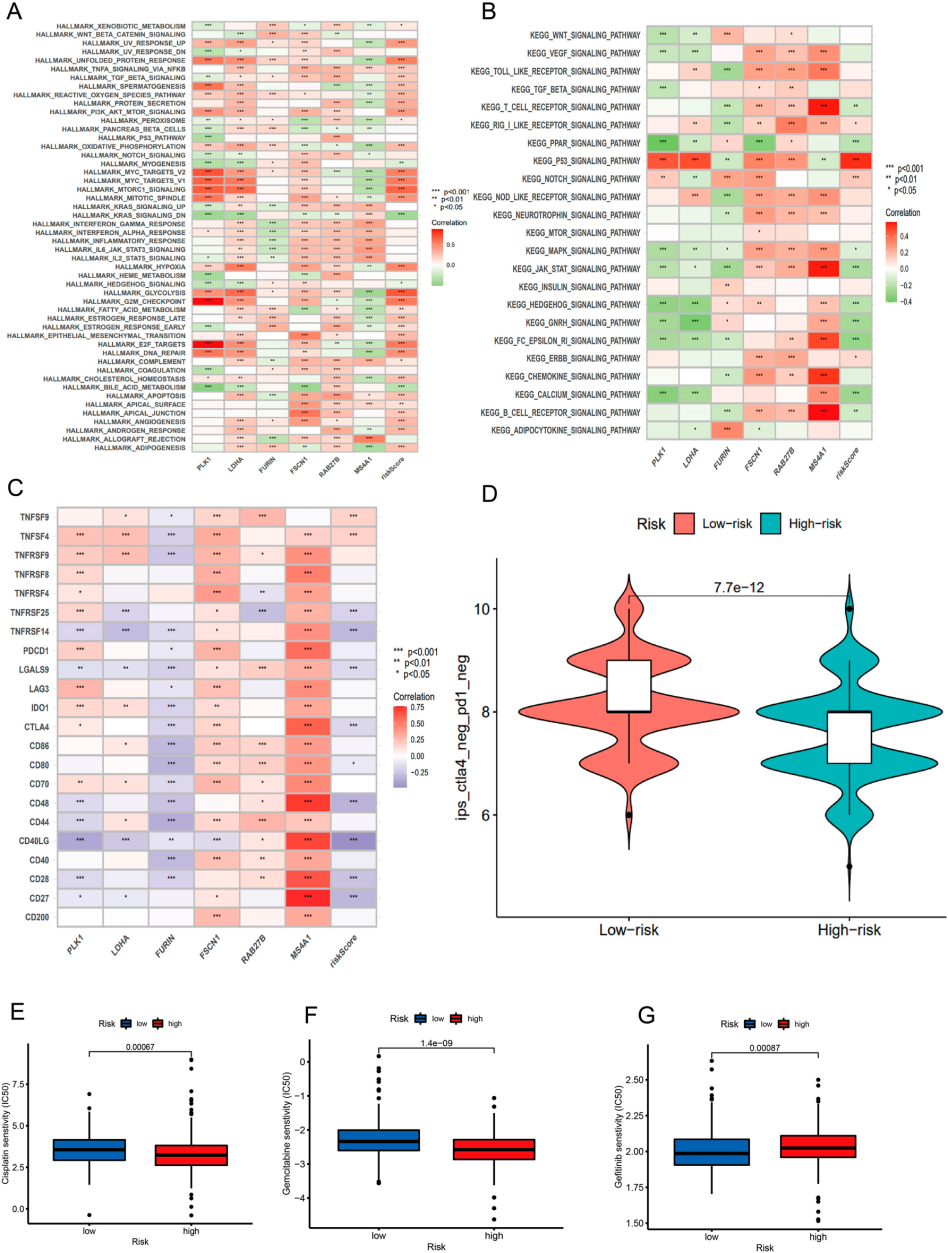
Gene Enrichment pathways of GSVA. (**A**) A heatmap illustrating the link between risk ratings and representative pathway entries on the KEGG database. (**B**) A heatmap illustrating the relationship between immunological signatures and risk scores. (**C**) The correlation between the levels of gene expression for immune checkpoint blockage and risk ratings. estimating how well immunotherapy will work. (**D**) IPS distribution map estimations of the effect that immune checkpoint inhibitors have on risk scores (**E**–**G**) A sensitivity analysis of cisplatin, gemcitabine, and gefitinib was performed on patients who had either a high or low-risk score.

According to the findings of the GSVA investigation, the individuals who belonged to the high-risk group exhibited increased apical junction and epithelial-mesenchymal transition, in addition to the activated MAPK pathway and chemokine signaling pathway **(**Fig. 10A,B**)**.

### Sensitivity of immunotherapy and chemotherapy prediction

We also try to predict the sensitivity of immunotherapy. 47 genes related to checkpoint blockade were retrieved (Table S4). The plot suggested that many check-point blockade-related genes (TNFSF4, PDCD1LG2, LAG3, CTLA4, and CD86) were positively correlated with risk scores (Fig. 10C). According to the risk assessment system, LRG has a high IPS score (PD1- negative, CTLA4-negative(Fig. 10D). In addition to this, we evaluated the efficacy of LUAD in combination with standard chemotherapy drugs such as cisplatin, docetaxel, and methotrexate. The newly identified indicators, such as IPS, TIDE score, and predicted neoantigens, were used to evaluate the possibility of immunotherapy response in TCGA-LUAD samples. Accordingly, we found that the PD1 and PD-L1 were significantly elevated in the high-risk group, and LAG3 was significantly decreased in the high-risk group (Supplementary Fig. S3B). Through analysis, we found that the IC50 of the three chemotherapeutic drugs (cisplatin, gemcitabine, and gefitinib) showed significant differences in HRG/LRG. The drug sensitivities of cisplatin (Fig. 10E) and gemcitabine (Fig. 10F) were higher in HRG than in LRG, whereas gefitinib (Fig. 10G) had higher drug sensitivities in LRG. These results suggest a potential link between the risk signature and chemotherapeutic drug sensitivity.

## Discussion

Lung cancer is a malignant tumor of the respiratory system with a high incidence, and it has the highest mortality rate among both men and women, non-small cell lung cancer (NSCLC) accounts for 80% of all lung cancer pathology types, and half of NSCLC is LUAD^18^. Targeting TME could assist traditional therapies and improve clinical responses in numerous cancer types^19^. Growing evidence has shown that TME has a significant impact on the growth and development of cancerous cells, and affects the response to immunotherapy.

In this research, samples of lung adenocarcinoma were interrogated with TME-related genes, among which high expression of PLK1, LDHA, FURIN, FSCN1, and RAB27B was correlated with poor OS, while high expression of MS4A1 was correlated with longer OS compared with the MS4A1 low expression. Researchers have demonstrated that PLK1 promotes the development of Kras/ Tp53-mutant lung adenocarcinoma through transcriptional activation of the receptor RET^20^. High expression of LDHA regulators contributes to the tumor microenvironment and predicts prognosis in lung adenocarcinoma^21^. FURIN correlated with immune infiltration serves as a potential biomarker in SARS-CoV-2 infection-related lung adenocarcinoma^22^. FSCN1-induced PTPRF-dependent tumor microenvironment inflammatory reprogramming promotes lung adenocarcinoma progression via regulating macrophagic glycolysis^23^. Rab27b Is a potential indicator for lymph node metastasis and unfavorable prognosis in lung adenocarcinoma^24^. MS4A1 dysregulation in asbestos-related lung squamous cell carcinoma is due to CD20 stromal lymphocyte expression^25^. The role of MS4A1 in tumor progression is still in the initial stage of research and is worthy of further exploration. In line with previous research, our study found that high TME risk was identified as a poor factor for OS in TCGA and GEO datasets^16^. In the field of lung cancer, the genes in our six-gene signature have not been extensively studied. However, the six-gene signature has a significant role in predicting and diagnosing LUAD in our research, indicating it or each gene in it may be the potential specific directions for future research on LUAD. Recently, we notice that there is another prognostic model built by an immune gene in LUAD^17^. however, when we compared our risk model with other prognosis risk signatures, the C-index demonstrated the highest AUC of our model. These results indicate that the overall performance of our proposed model is superior to others.

To explore the biological function of these six TME-related genes, we performed enrichment analysis and found that in lung adenocarcinoma, TME was most related to the MAPK pathway and chemokine signaling pathway. Chemotherapy is the main adjuvant treatment for lung urothelial carcinoma, and immune checkpoint inhibitors are the most promising new treatment. The immune checkpoints PD-L1/CD274, CTLA4, and B7-H3/CD276 were significantly correlated with the risk scores. The combination of CTLA4 antibody, tremelimumab, and durvalumab in the treatment of metastatic lung cancer is in progress^26^. Anti-CD3 antibody chemically coupled with anti-B7-H3 mAb antibody has been clinically approved^27^.

As to treatment strategies, the high-risk group has a higher expression of co-inhibitory factors and is more resistant to common chemotherapy including cisplatin, gemcitabine, and gefitinib. The risk assessment demonstrated that on the gene set level, the TME-related gene set indicates higher disease risk, and more potential to perform immune evasion and establish chemotherapy resistance. Although this research is mainly based on bioinformatics analysis, this is an innovative and cutting-edge study based on big data and could serve as assistance to clinical practice.

## Conclusion

Our results demonstrated that TME-related gene signature shows potential roles in the prediction of prognosis and immunotherapy response in LUAD patients. In addition, the risk model is remarkably associated with immune cell infiltration and modulates the T cell function in LUAD, implying the potential role in predicting immunotherapy response. Therefore, the TME-related gene signature can provide recommendations for improving patients’ response to immunotherapy and promote personalized tumor immunotherapy in the future.

### Supplementary Information

Supplementary Information.

## Data Availability

The datasets analyzed during the current study are available in The Cancer Genome Atlas database (TCGA-LUAD, https://tcga-data.nci.nih.gov/tcga/) and the Gene Expression Omnibus database [GSE68571(https://www.ncbi.nlm.nih.gov/geo/query/acc.cgi), GSE50081(https://www.ncbi.nlm.nih.gov/geo/query/acc.cgi), GSE13213(https://www.ncbi.nlm.nih.gov/geo/query/acc.cgi) and GSE10072(https://www.ncbi.nlm.nih.gov/geo/query/acc.cgi)].

## References

[1] Ferlay J (2021). Cancer statistics for the year 2020: An overview. Int. J. Cancer.

[2] Fradet Y (2019). Randomized phase III KEYNOTE-045 trial of pembrolizumab versus paclitaxel, docetaxel, or vinflunine in recurrent advanced urothelial cancer: Results of >2 years of follow-up. Ann. Oncol..

[3] Cella D (2019). Patient-reported outcomes of patients with advanced renal cell carcinoma treated with nivolumab plus ipilimumab versus sunitinib (CheckMate 214): a randomised, phase 3 trial. Lancet Oncol..

[4] Hodi FS (2018). Nivolumab plus ipilimumab or nivolumab alone versus ipilimumab alone in advanced melanoma (CheckMate 067): 4-Year outcomes of a multicentre, randomised, phase 3 trial. Lancet Oncol..

[5] Kang YK (2017). Nivolumab in patients with advanced gastric or gastro-oesophageal junction cancer refractory to, or intolerant of, at least two previous chemotherapy regimens (ONO-4538-12, ATTRACTION-2): A randomised, double-blind, placebo-controlled, phase 3 trial. Lancet.

[6] Topalian SL (2012). Safety, activity, and immune correlates of anti-PD-1 antibody in cancer. N. Engl. J. Med..

[7] Jenkins BH (2022). Targeting cancer-associated fibroblasts: Challenges, opportunities and future directions. Pharmacol. Ther..

[8] Quail DF, Joyce JA (2013). Microenvironmental regulation of tumor progression and metastasis. Nat. Med..

[9] Schulz M (2019). Microenvironmental regulation of tumor progression and therapeutic response in brain metastasis. Front. Immunol..

[10] Gilazieva Z (2022). The dual role of mesenchymal stromal cells and their extracellular vesicles in carcinogenesis. Biology.

[11] Giraldo NA (2019). The clinical role of the TME in solid cancer. Br. J. Cancer.

[12] Mao X (2021). Crosstalk between cancer-associated fibroblasts and immune cells in the tumor microenvironment: New findings and future perspectives. Mol. Cancer.

[13] Madeddu C (2022). EGFR-mutated non-small cell lung cancer and resistance to immunotherapy: Role of the tumor microenvironment. Int. J. Mol. Sci..

[14] Cairns RA, Harris IS, Mak TW (2011). Regulation of cancer cell metabolism. Nat. Rev. Cancer.

[15] Rooney MS (2015). Molecular and genetic properties of tumors associated with local immune cytolytic activity. Cell.

[16] Wu J (2021). A risk model developed based on tumor microenvironment predicts overall survival and associates with tumor immunity of patients with lung adenocarcinoma. Oncogene.

[17] Yue C, Ma H, Zhou Y (2019). Identification of prognostic gene signature associated with microenvironment of lung adenocarcinoma. PeerJ.

[18] Hua FF (2017). MiRNA-338-3p regulates cervical cancer cells proliferation by targeting MACC1 through MAPK signaling pathway. Eur. Rev. Med. Pharmacol. Sci..

[19] Bejarano L, Jordāo MJC, Joyce JA (2021). Therapeutic targeting of the tumor microenvironment. Cancer Discov..

[20] Kong Y (2022). The kinase PLK1 promotes the development of Kras/Tp53-mutant lung adenocarcinoma through transcriptional activation of the receptor RET. Sci. Signal.

[21] Shang S (2022). Lactate regulators contribute to tumor microenvironment and predict prognosis in lung adenocarcinoma. Front. Immunol..

[22] Luo L (2022). FURIN correlated with immune infiltration serves as a potential biomarker in SARS-CoV-2 infection-related lung adenocarcinoma. Clin. Exp. Med..

[23] Huang Y (2022). FSCN1 induced PTPRF-dependent tumor microenvironment inflammatory reprogramming promotes lung adenocarcinoma progression via regulating macrophagic glycolysis. Cell Oncol..

[24] Zhang L (2018). Rab27b is a potential indicator for lymph node metastasis and unfavorable prognosis in lung adenocarcinoma. Dis. Mark..

[25] Wright CM (2012). MS4A1 dysregulation in asbestos-related lung squamous cell carcinoma is due to CD20 stromal lymphocyte expression. PLoS ONE.

[26] Zhou WT, Jin WL (2021). B7–H3/CD276: An emerging cancer immunotherapy. Front. Immunol..

[27] Ma J (2016). B7–H3 as a promising target for cytotoxicity T cell in human cancer therapy. Oncotarget.

