# Supplementary material for: Identification and validation of tumor microenvironment-related signature for predicting prognosis and immunotherapy response in patients with lung adenocarcinoma
Source: Sci Rep. 2023 Aug 21;13:13568. doi: 10.1038/s41598-023-40980-2 (PMC10442419; doi:10.1038/s41598-023-40980-2)
Supplement: Supplementary file 1 — Supplementary Information. [file 41598_2023_40980_MOESM1_ESM.docx]

**Supplementary Materials**


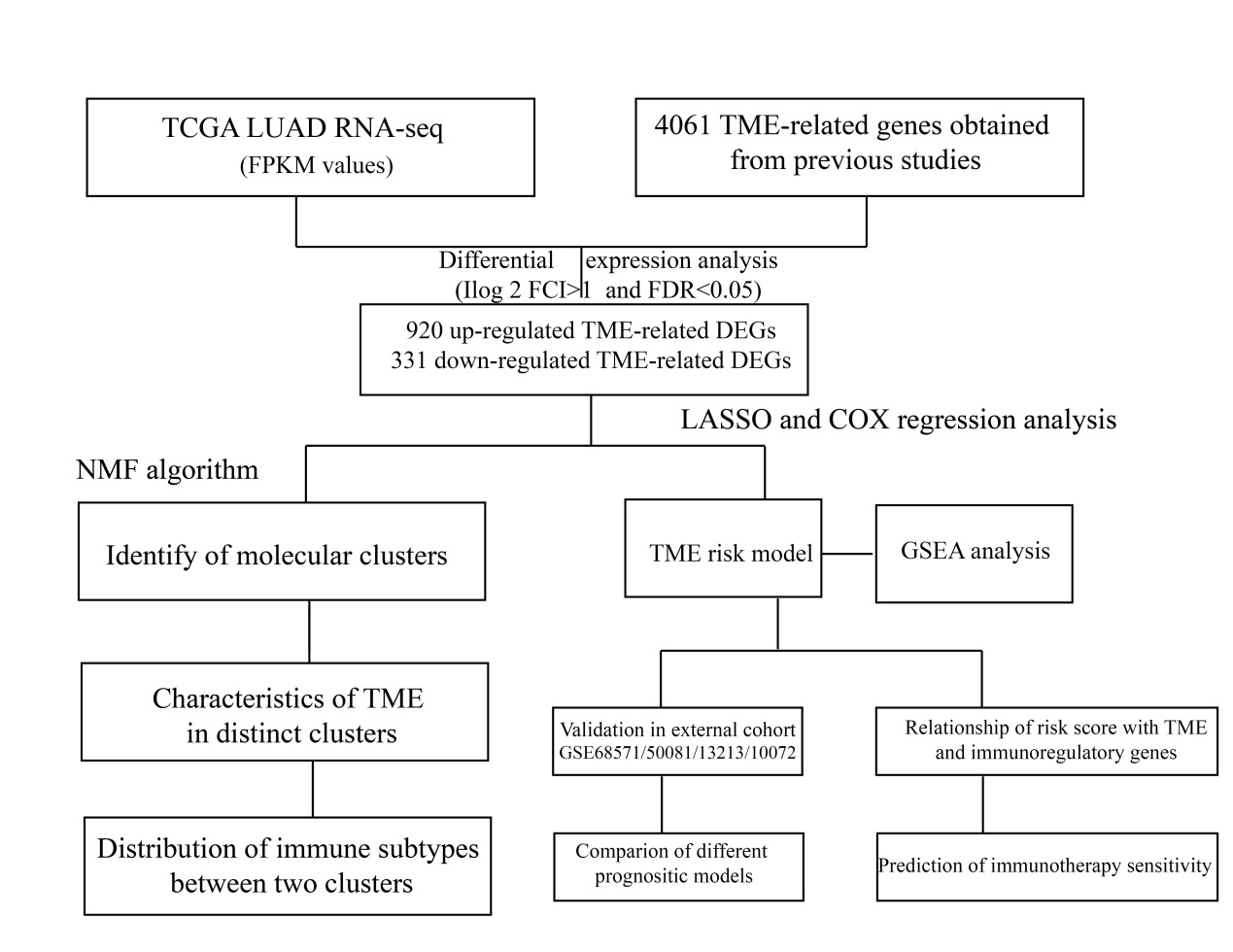


**Supplementary Figure 1 The flow chart describing this study.**


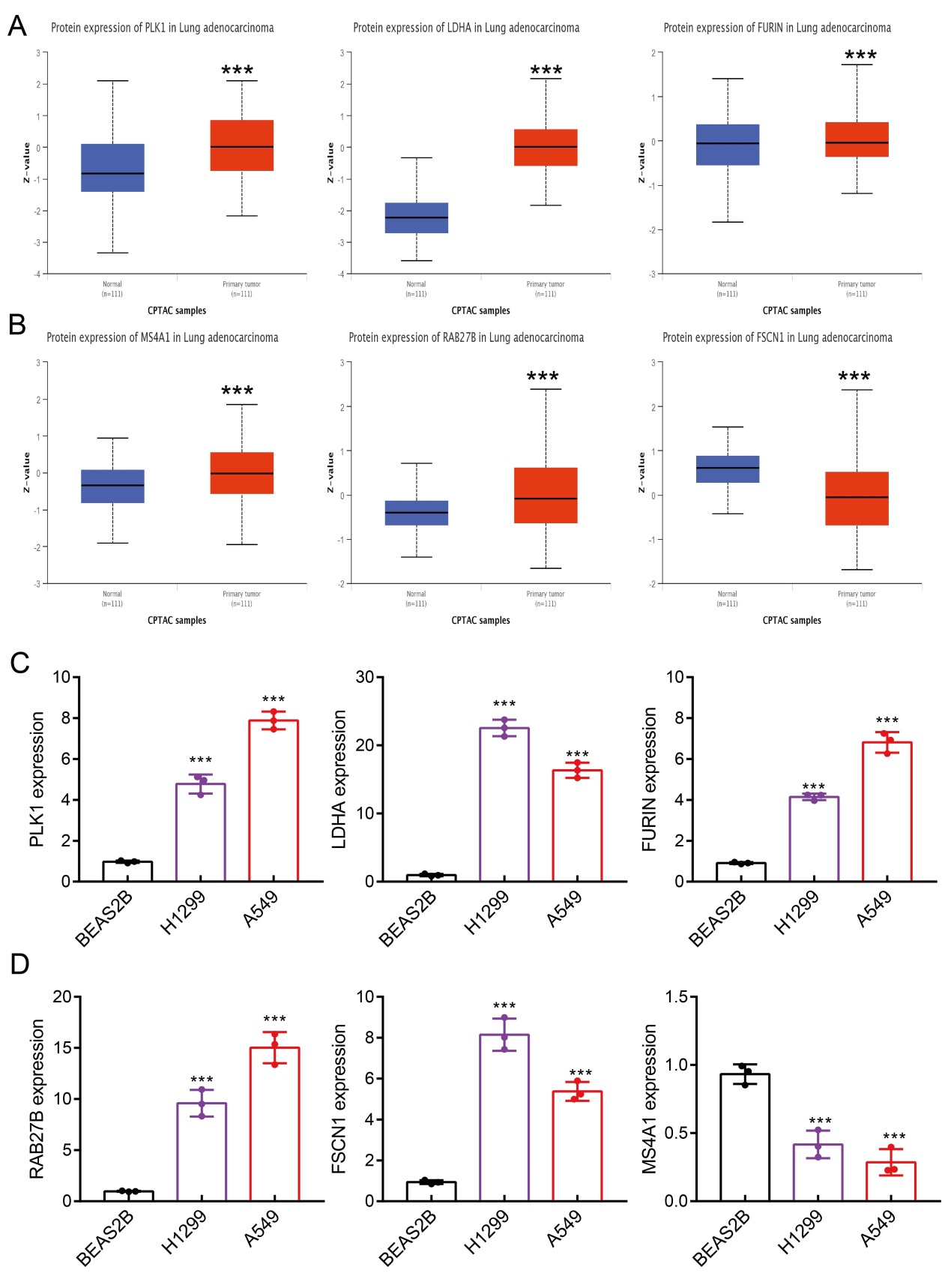


**Supplementary Figure 2 Validation of TME-related gene expression in lung cancer cells.** (A-B) The protein expression of TME-related in lung cancer tissues by Clinical Proteomic Tumor Analysis Consortium. (C-D) The RNA expression of TME-related in lung cancer cells by using qPCR assay.


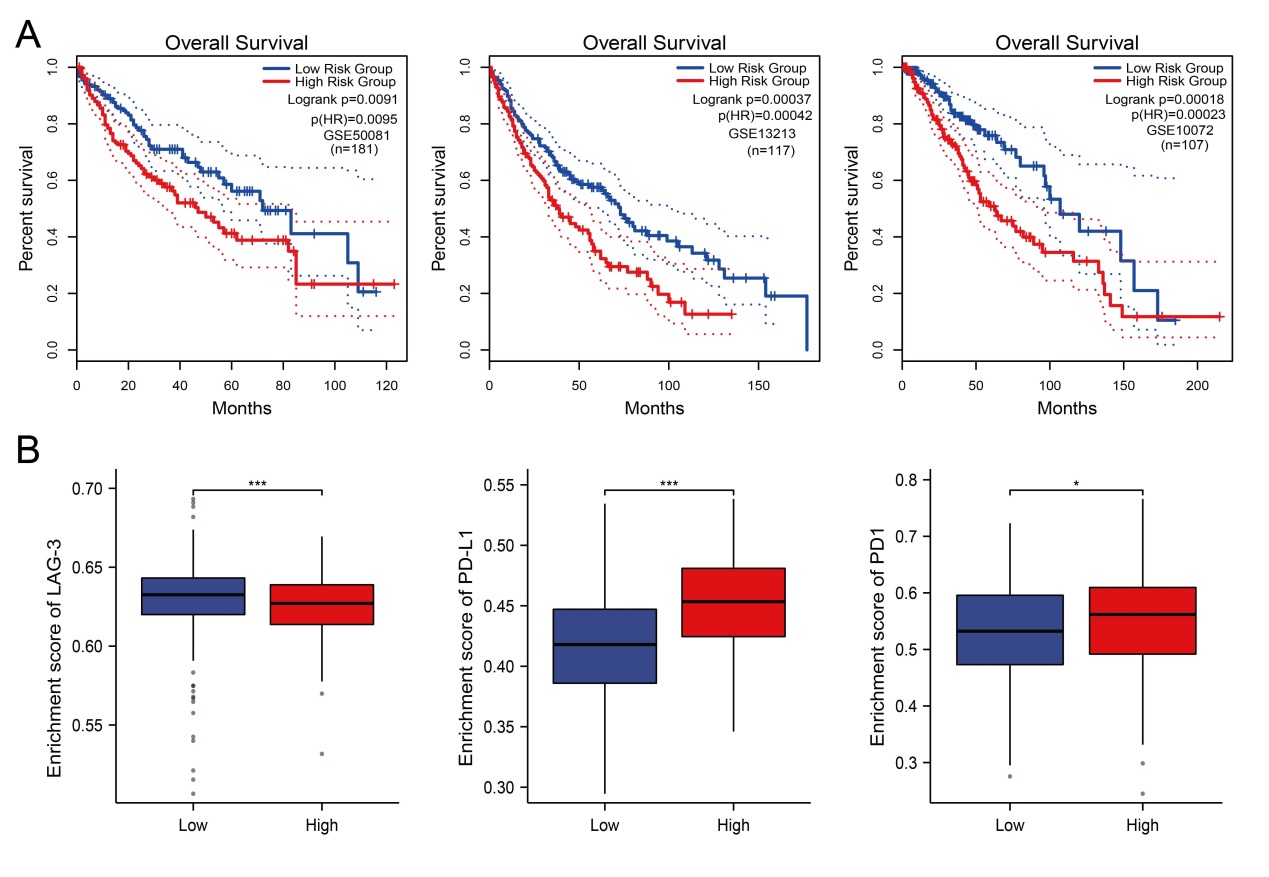


**Supplementary Figure 3 Validation of the prognosis and immune role of TME-related gene in LUAD.** (A) Kaplan-Meier survival curves of TME-related gene in lung cancer by GEO cohort. (B) High-risk patients had higher expression of PD-1, PDL-1, and lower expression of LAG-3.

**Supplementary Table 1:** The TME-related genes were obtained from previous studies.

**Supplementary Table 2:** There were 1251 TME-associated genes (FDR<0.05 and |log2FC|>1) differentiated in expression between LUAD and normal lung tissues.

**Supplementary Table 3:** There were 203 prognostic TME-related genes obtained by univariate Cox analysis in LUAD.

**Supplementary Table 4:** There were 47 genes related to checkpoint blockade.

**Supplementary Table 1:** The TME-related genes were obtained from previous studies.

| WINT | FAM110B | CNR1 | GNLY | SLC2A1 | MAP3K1 | MX2 | RNASE10 | TUBA3D |
| --- | --- | --- | --- | --- | --- | --- | --- | --- |
| SRPK1 | CDH2 | AP3S1 | CXCL16 | PIK3R2 | GTSE1 | HSPBP1 | NPIPB3 | SETMAR |
| PPP1R2 | ACP5 | TRAPPC1 | BCL11A | LSM4 | DDX60 | ELOA3 | JAG2 | PBRM1 |
| MIEN1 | THADA | SCAMP3 | VILL | GPNMB | C11orf80 | CCT6B | FBXO30 | LAMP2 |
| HIST1H3J | RCC1 | ORC1 | SLC24A2 | CYP27A1 | ZBTB10 | SYT8 | CFP | GADD45GIP1 |
| DPP10 | NF2 | KLF1 | PHF24 | BIK | SMIM10L2B | PTGIS | AFAP1L2 | CPNE1 |
| CASP3 | IL7R | FOXP1 | LRP5L | VWA5B1 | PLIN2 | MX1 | TMEM178A | ASAH2 |
| ZSCAN5B | FAM102A | CNPY4 | GNG7 | SLC29A4 | MAOB | HSPB7 | RMND1 | TTYH3 |
| SRMS | CDH19 | AP3M2 | CXCL14 | PIK3IP1 | GTF3C5 | ELOA2 | NPIPB15 | SETDB1 |
| PPP1R16B | ACOT9 | TRANK1 | BCAT2 | LSM2 | DDX58 | CCT6A | JAG1 | PBK |
| MICOS13 | TGM5 | SCAMP1 | VHLL | GPM6B | C11orf53 | SYT5 | FBXO27 | LAMC1 |
| HIST1H3H | RCAN3 | OR6B2 | SLC22A4 | CYP26C1 | ZBP1 | PTGIR | CFLAR | GADD45A |
| DPM3 | NEUROD2 | KIT | PHEX | BHLHE41 | SMIM10L2A | MUC20 | AFAP1L1 | CPM |
| CASKIN1 | IL7 | FOXN4 | LRP4 | VWA5A | PLEKHO2 | HSPB6 | TMEM161B | ARX |
| ZSCAN10 | FADS1 | CNPY3 | GNG11 | SLC29A3 | MANEAL | ELN | RMI2 | TTLL4 |
| SRL | CDH12 | AP2S1 | CXCL13 | PIK3CD | GTF3A | CCT5 | NPFFR1 | SETD7 |
| PPP1R16A | ACKR2 | TRAIP | BCAT1 | LSM1 | DDX53 | SYT2 | IZUMO2 | PAXX |
| MICB | TGM3 | SBK1 | VGLL3 | GPI | C11orf45 | PTGFRN | FBXO17 | LAMB4 |
| HIST1H3F | RBPJL | OR5B12 | SLC22A15 | CYP1B1 | ZAP70 | MTX1 | CFL1 | GAD1 |
| DPM1 | NEUROD1 | KISS1R | PHC3 | BHLHE40 | SMG5 | HSPB1 | AEN | CPLX2 |
| CASD1 | IL6ST | FOXM1 | LRP1 | VTI1B | PLEKHH2 | ELMO2 | TMEM160 | ART5 |
| ZPBP2 | FADD | CNN1 | GNAT1 | SLC29A2 | MANEA | CCT3 | RLN2 | TTK |
| SRGN | CDH1 | AP2M1 | CXCL12 | PIGX | GTF2F2 | SYT17 | NPEPL1 | SET |
| PPP1R14B | ACHE | TRAF5 | BCAP31 | LSG1 | DDX49 | PTGER4 | ITPRIPL2 | PAX7 |
| MICAL3 | TGM2 | SBDS | VGLL1 | GPC4 | C10orf82 | MTUS1 | FBXL8 | LAMB1 |
| HIST1H3A | RBPJ | OR52I1 | SLC1A7 | CYP11A1 | YME1L1 | HSPA6 | CFHR3 | GABRR1 |
| DPF1 | NEURL2 | KISS1 | PGPEP1L | BFSP1 | SMG1 | ELMO1 | ADRM1 | CPLX1 |
| CARD9 | IL5RA | FOXJ1 | LRMP | VSTM2L | PLEKHG4 | CCT2 | TMEM156 | ARSH |
| ZP3 | FABP6 | CNIH2 | GNAS | SLC29A1 | MAN1B1 | SYT15 | RLBP1 | TTC7B |
| SRD5A3 | CDCA8 | AP1S1 | CXCL11 | PIGU | GSTCD | PTGER2 | NPDC1 | SESN3 |
| PPP1CC | ACBD7 | TRAF4 | BBS4 | LRRTM1 | DDX41 | MTSS2 | ITM2A | PAX6 |
| MICAL2 | TGIF2LX | SAV1 | VEPH1 | GPC3 | C10orf62 | HSPA4 | FBXL6 | LAMA5 |
| HIST1H2BO | RBP3 | OR51E1 | SLC1A3 | CYLD | YJEFN3 | ELK3 | CETN3 | GABRQ |
| DPEP2 | NEU1 | KIRREL3 | PGP | BEX5 | SMCO3 | CCR9 | ADRB2 | CPEB2 |
| CARD8 | IL5 | FOXI3 | LRIT3 | VSIR | PLEKHG3 | SYT10 | TMEM150C | ARSG |
| ZNF99 | FABP5 | CNGB3 | GNAI1 | SLC27A6 | MAN1A1 | PTGDS | RIPPLY3 | TTC39A |
| SRCIN1 | CDCA7 | AP1M1 | CXCL10 | PIGO | GSTA4 | MTNR1B | NPB | SERTAD2 |
| PPM1N | ACAP1 | TRAF3IP3 | BBS12 | LRRN4CL | DDX4 | HSPA1B | ITK | PAX5 |
| MIA | TGIF1 | SASS6 | VEGFB | GPC2 | BZW2 | ELFN2 | FBXL20 | LAIR2 |
| HIST1H2BN | RBP1 | OR2B6 | SLC18A2 | CYBRD1 | YIPF2 | CCR7 | CERS6 | GABRG3 |
| DPCD | NETO2 | KIRREL1 | PGLYRP1 | BEST1 | SMC5 | SYNJ1 | ADORA1 | CPE |
| CARD19 | IL4R | FOXI2 | LRIG1 | VSIG4 | PLEKHF2 | PTGDR2 | TMEM145 | ARRB1 |
| ZNF92 | FABP4 | CNGB1 | GMPS | SLC26A8 | MALL | MTMR7 | RIPOR2 | TTC38 |
| SRC | CDCA5 | AOX1 | CXCL1 | PIGL | GSS | HSPA1A | NPAS3 | SERPINI1 |
| PPM1L | ACAN | TRAF3IP2 | BBC3 | LRRN3 | DDX3Y | ELF1 | ITIH6 | PAX3 |
| MGST1 | TGFBR2 | SASH3 | VEGFA | GPC1 | BUB3 | CCR6 | FBXL17 | LAIR1 |
| HIST1H2BL | RBMS3 | OR1L8 | SLC17A9 | CYBB | YIF1B | SYNGR3 | CERS4 | GABRD |
| DONSON | NEMP2 | KIR3DL2 | PGK2 | BEND5 | SMC4 | PTGDR | ADIRF | CPD |
| CARD11 | IL4I1 | FOXI1 | LRGUK | VPS28 | PLEKHF1 | MTHFD2 | TMEM140 | ARNTL |
| ZNF91 | FABP3 | CMTM2 | GMNN | SLC26A6 | MAL | HSF4 | RIMS4 | TSTA3 |
| SRA1 | CDCA4 | AOC1 | CWF19L1 | PIF1 | GSG1 | ELAVL2 | NPAS1 | SERPINF1 |
| PPM1J | ACADM | TRAF2 | BASP1 | LRRN1 | DDX3X | CCR5 | ITIH2 | PASK |
| MGAT5B | TFRC | SARS2 | VCX3A | GPBP1 | BUB1B | SYNE4 | FBXL16 | LAGE3 |
| HIST1H2BJ | RBM5 | OR1J4 | SLC17A5 | CYB5B | YEATS4 | PTDSS2 | CERS1 | GABRB2 |
| DOK5 | NELFCD | KIR3DL1 | PGGHG | BEND4 | SMC2 | MTHFD1L | ADGRL4 | CPA3 |
| CAPZA1 | IL4 | FOXH1 | LRG1 | VPREB3 | PLEKHB2 | HSF2BP | TMEM132A | ARNT |
| ZNF90 | FABP1 | CMTM1 | GMDS | SLC26A5 | MAK | ELANE | RIMS2 | TSSK6 |
| SQSTM1 | CDCA3 | ANXA5 | CUZD1 | PIEZO1 | GRPR | CCR3 | NOXO1 | SERPINE2 |
| PPM1H | ACADL | TRAF1 | BARX2 | LRRIQ4 | DDX39A | SYN3 | ITGB8 | PARVB |
| MGAM | TFPT | SARDH | VCX | GPATCH2 | BUB1 | PTDSS1 | FBN3 | LAG3 |
| HIST1H2BH | RBM3 | OR1J2 | SLC16A8 | CXorf40A | YEATS2 | MTFR2 | CEP78 | GABRA3 |
| DOCK9 | NEK5 | KIR2DL4 | PGF | BCL7A | SMC1B | HSD17B3 | ADGRG3 | COX7C |
| CAPN3 | IL3RA | FOXG1 | LRFN5 | VOPP1 | PLEKHB1 | EIF4A1 | TMEM129 | ARMT1 |
| ZNF878 | FAAP24 | CMKLR1 | GM2A | SLC26A2 | MAJIN | CCR2 | RILPL2 | TSSK1B |
| SPX | CDCA2 | ANXA4 | CTU1 | PICALM | GRM8 | SYCP2L | NOXA1 | SERPINA3 |
| PPM1F | ACAD8 | TRADD | BARX1 | LRRC56 | DDX17 | PTCRA | ITGB7 | PARVA |
| MFSD4B | TFPI | SAPCD2 | VCAN | GPAT4 | BTNL8 | MTERF3 | FBN2 | LACTB2 |
| HIST1H2BG | RBM20 | OPRD1 | SLC16A7 | CXCR6 | YBX2 | HSD17B11 | CEP72 | GABPB1 |
| DOCK6 | NEK3 | KIR2DL3 | PGC | BCL6B | SMAD4 | EIF3L | ADGRG1 | COX7B2 |
| CAPN13 | IL37 | FOXE3 | LRFN4 | VNN3 | PLEKHA4 | CCR10 | TMEM119 | ARMCX3 |
| ZNF789 | F8A1 | CMA1 | GLYATL2 | SLC25A6 | MAGOHB | SYCP2 | RIBC2 | TSPAN7 |
| SPTSSB | CDC7 | ANTXR1 | CTTNBP2 | PIAS3 | GRM5 | PTCHD4 | NOX4 | SERPINA1 |
| PPM1E | ACACB | TPX2 | BANK1 | LRRC52 | DDX11 | MTCP1 | ITGB4 | PARPBP |
| MFSD3 | TFF2 | SAMSN1 | VCAM1 | GPAA1 | BTN3A2 | HSD11B2 | FBN1 | L1CAM |
| HIST1H2BF | RBIS | OPCML | SLC16A6 | CXCR5 | YBX1 | EIF3H | CEP68 | GABARAP |
| DOCK5 | NEK2 | KIR2DL1 | PGAM4 | BCL6 | SMAD2 | CCR1 | ADGRF5 | COX7B |
| CAPN12 | IL34 | FOXD4L6 | LRFN1 | VNN2 | PLEK2 | SYCE3 | TMEM116 | ARMC12 |
| ZNF747 | F8 | CLUAP1 | GLUL | SLC25A52 | MAGED4 | PTBP1 | RHPN1 | TSPAN5 |
| SPTAN1 | CDC6 | ANP32E | CTTN | PI3 | GRM2 | MTBP | NOX3 | SERINC4 |
| PPM1B | ACACA | TPTE | BANF1 | LRRC45 | DDR1 | HSD11B1 | ITGB2 | PARK7 |
| MFSD2B | TFF1 | SAMD10 | VASN | GOLT1B | BTN2A2 | EIF2S2 | FBLN5 | KYNU |
| HIST1H2BC | RBCK1 | OPA1 | SLC16A3 | CXCR3 | YAE1 | CCNO | CEP57 | GAB3 |
| DOCK2 | NEIL3 | KIFC2 | PFN2 | BCL2L12 | SMAD1 | SYCE2 | ADGRE5 | COX6B1 |
| CAPG | IL32 | FOXD4L5 | LRCH4 | VNN1 | PLEK | PTAFR | TMEM101 | ARL9 |
| ZNF716 | F3 | CLTC | GLUD1 | SLC25A40 | MAGED2 | MSRB1 | RHOU | TSPAN15 |
| SPSB4 | CDC5L | ANOS1 | CTSZ | PHYHIPL | GRM1 | HS6ST3 | NOX1 | SERINC3 |
| PPIB | ABTB1 | TPST1 | BAMBI | LRRC42 | DDIT3 | EIF2AK2 | ITGB1 | PAQR6 |
| MFSD10 | TFEC | SAMD1 | VASH2 | GOLM1 | BTLA | CCNI2 | FBLN1 | KSR1 |
| HIST1H2BB | RBBP8NL | ONECUT2 | SLC16A14 | CXCR2 | XXYLT1 | SYCE1L | CEP55 | G6PD |
| DOCK10 | NEFL | KIFC1 | PFKP | BCL2L10 | SLITRK6 | PSTPIP1 | ADGRE3 | COX6A1 |
| CAMP | IL3 | FOXD4L4 | LPIN3 | VN1R1 | PLD5 | MSR1 | TMEFF2 | ARL6 |
| ZNF710 | F2RL3 | CLTB | GLT8D2 | SLC25A37 | MAGED1 | HS3ST2 | RHOT2 | TSLP |
| SPRY3 | CDC45 | ANO9 | CTSW | PHRF1 | GRK7 | EIF1B | NOTUM | SERINC1 |
| PPIAL4G | ABT1 | TPSB2 | BACH2 | LRRC4 | DDIAS | CCNF | ITGAX | PAQR5 |
| MFGE8 | TFDP3 | SAGE1 | VASH1 | GOLGA8B | BTG3 | SYCE1 | FBLL1 | KRTCAP2 |
| HIST1H2AM | RAX | OLR1 | SLC16A11 | CXCR1 | XRCC6 | PSRC1 | CEP152 | G0S2 |
| DOC2B | NEDD4L | KIF4B | PFKM | BCL2L1 | SLITRK1 | MSMP | ADGRE2 | COTL1 |
| CAMLG | IL2RB | FOXD4L1 | LPGAT1 | VMAC | PLD4 | HRK | TMED2 | ARL17A |
| ZNF707 | F2RL2 | CLSPN | GLS2 | SLC25A35 | MAGEC3 | EIF1 | RHOJ | TSKS |
| SPRY2 | CDC25C | ANO7 | CTSV | PHOSPHO1 | GRINA | CCNE2 | NOS3 | SERHL2 |
| PPIAL4C | ABI3BP | TPSAB1 | BACE2 | LRRC26 | DCUN1D5 | SVOPL | ITGAM | PAQR4 |
| MFAP5 | TFCP2L1 | SACM1L | VAPA | GOLGA8A | BST2 | PSPN | FATE1 | KRTAP1-5 |
| HIST1H2AL | RASSF5 | OLIG1 | SLC15A4 | CXCL9 | XRCC3 | MSLNL | CEP131 | FZR1 |
| DOC2A | NECTIN3 | KIF4A | PFKFB4 | BCL2A1 | SLIT1 | HRH4 | ADGRE1 | CORT |
| CAMKV | IL2RA | FOXD4 | LPAR5 | VLDLR | PLCL2 | EID1 | TMC6 | ARL1 |
| ZNF703 | F13A1 | CLPTM1L | GLRB | SLC25A21 | MAGEC2 | CCNE1 | RHOB | TSHR |
| SPP1 | CDC25B | ANO5 | CTSK | PHLDA1 | GRIN3A | SVEP1 | NOS2 | SERGEF |
| PPIA | ABHD5 | TPM2 | BAALC | LRRC25 | DCSTAMP | PSPH | ITGAL | PAPSS2 |
| MFAP4 | TFAP2E | SAC3D1 | VANGL2 | GOLGA7B | BST1 | MSL1 | FASN | KRT80 |
| HIST1H2AJ | RASSF4 | OLFML3 | SLC15A3 | CXCL5 | XRCC2 | HRH2 | CENPW | FZD8 |
| DNTTIP1 | NECTIN2 | KIF3C | PFDN2 | BCL2 | SLFN13 | EGR3 | ADGRB2 | CORO7 |
| CAMKK2 | IL26 | FOXD1 | LPAR2 | VIRMA | PLCH2 | CCND2 | TMBIM6 | ARID5A |
| ZNF695 | F12 | CLPSL2 | GLP1R | SLC24A5 | MAGEC1 | SV2C | RHOA | TSGA13 |
| SPON2 | CDC25A | ANLN | CTSG | PHKA1 | GRIN2D | PSPC1 | NOP58 | SEPTIN8 |
| PPFIBP2 | ABHD3 | TPM1 | B4GALT7 | LRRC14B | DCST2 | MSI1 | ITGA9 | PANX2 |
| MFAP3L | TFAP2B | S1PR5 | VAMP2 | GOLGA6L1 | BSND | HRH1 | FASLG | KRT74 |
| HIST1H2AG | RASIP1 | OLFML2B | SLC15A2 | CXCL3 | XPR1 | EGR2 | CENPO | FZD6 |
| DNMT3B | NDUFS8 | KIF2C | PF4 | BCL11B | SLF1 | CCND1 | ADGRB1 | CORO1A |
| CAMK2N2 | IL22 | FOXA1 | LPAR1 | VIM | PLCG2 | SUV39H1 | TM9SF4 | ARID4B |
| ZNF692 | F11R | CLPSL1 | GLOD4 | SLC24A3 | MAGEA6 | PSMG3 | RHEBL1 | TSC22D3 |
| SPON1 | CDC20B | ANKS3 | CTSE | PHF7 | GRIN2B | MSH5 | NOP10 | SEPTIN5 |
| PPFIBP1 | ABHD2 | TPK1 | B4GALT6 | LRP8 | DCST1 | HPSE | ITGA4 | PAMR1 |
| MEX3A | TEX45 | S1PR1 | VAC14 | ACTL10 | BSN | EGLN3 | FARP1 | KRT23 |
| HIST1H2AE | RASGRP3 | OIP5 | SLC12A8 | THOC6 | XPOT | CCNB3 | CENPN | FZD4 |
| DNMT3A | NDUFS6 | KIF26B | PEX5L | RECQL4 | SLCO5A1 | SUSD3 | ADD2 | COQ9 |
| CALR | IL21R | FOSL1 | LOXL4 | NFE4 | PLCB4 | PSMG1 | TM6SF1 | ARID3A |
| ZNF681 | EZR | CLNS1A | GLIPR1 | ING3 | MAGEA3 | MSH4 | RHBG | TSC22D1 |
| SPOCK2 | CDC20 | ANKRD9 | CTRL | FAM166A | GRID2 | HPS4 | NOMO3 | SEMA6B |
| PPFIA4 | ABHD17A | TPI1 | B4GALT3 | CDK1 | DCN | EGFR | ITGA2B | PAM |
| METTL9 | TEX38 | S100B | UTS2B | ACTG1 | BRSK2 | CCNB2 | FAP | KREMEN2 |
| HIST1H2AD | RASGRP2 | OGT | SLC12A6 | THEMIS2 | XPO6 | SULT4A1 | CENPM | FZD3 |
| DNMT1 | NDUFB9 | KIF24 | PEX10 | REC8 | SLCO4C1 | PSME3 | ADCYAP1 | COPZ2 |
| CALN1 | IL21 | FOSB | LOX | NFE2L3 | PLBD1 | MSC | TM4SF4 | ARHGEF39 |
| ZNF668 | EZH2 | CLNK | GLDN | INA | MAGEA2 | HPGDS | RHBDD3 | TSACC |
| SPOCK1 | CDC14A | ANKRD55 | CTPS2 | FAM153B | GREM2 | EGFL7 | NOMO2 | SEMA4A |
| PPFIA2 | ABHD16B | TPBG | B4GALNT1 | CDIP1 | DCLK3 | CCNB1 | ITGA11 | PALMD |
| METTL27 | TEX264 | S100A9 | UTP20 | ACTA2 | BRMS1 | SULT1C4 | FANCI | KRBA1 |
| HIST1H1D | RASGRP1 | OGDHL | SLC12A5 | THEMIS | XKRX | PSMD4 | CENPL | FZD2 |
| DNM1 | NDUFAF6 | KIF23 | PELO | RDX | SLCO2B1 | MSANTD3 | ADCY9 | COPE |
| CALHM3 | IL1RN | FOLH1 | LONRF1 | NFE2L2 | PLAUR | HPGD | TM4SF19 | ARHGEF38 |
| ZNF629 | EYS | CLN8 | GLDC | IMPG1 | MAGEA12 | EGFL6 | RGS4 | TRPV6 |
| SPN | CDA | ANKRD39 | CTNS | FAM136A | GREM1 | CCNA2 | NOL7 | SEMA3F |
| PPFIA1 | ABHD11 | TP63 | B3GNT4 | CDHR1 | DCLK2 | SULT1C2 | ISM2 | PALLD |
| METTL26 | TEX19 | S100A5 | USP9Y | ACSS3 | BRIX1 | PSMD3 | FANCG | KPNA7 |
| HIST1H1C | RASGRF2 | OGA | SLC12A2 | THEM6 | XKR7 | MS4A7 | CENPK | FYN |
| DND1 | NDUFA8 | KIF21A | PEG10 | RDM1 | SLCO2A1 | HPDL | ADCY4 | COMMD5 |
| CALHM1 | IL1RL1 | FNIP2 | LNP1 | NFE2 | PLAU | EFNB1 | TM4SF18 | ARHGEF15 |
| ZNF609 | EYA2 | CLN6 | GLB1L3 | IMPDH2 | MAGEA11 | CCNA1 | RGS3 | TRPV2 |
| SPIRE1 | CD96 | ANKRD34B | CTNND2 | FAM135B | GRB7 | SULF1 | NOL4 | SEMA3B |
| PPEF1 | ABCG2 | TP53TG3B | B3GAT1 | CDH8 | DCAF8L2 | PSMD2 | ISLR | PAK5 |
| METTL21A | TEX14 | S100A4 | USP40 | ACSM1 | BRIP1 | MS4A6A | FANCE | KPNA2 |
| HIST1H1B | RASA4 | OFD1 | SLC12A1 | THEG | XKR4 | HPCA | CENPI | FYB1 |
| DNASE2B | NDUFA7 | KIF20A | PECAM1 | RCSD1 | SLCO1C1 | EFNA5 | ADCY1 | COMMD4 |
| CALD1 | IL1RAPL1 | FN1 | LMNB2 | NFATC4 | PLAGL2 | CCN4 | TLX3 | ARHGEF1 |
| ZNF600 | EXT2 | CLK4 | GK | IMPDH1 | MAGEA10 | SUB1 | RGS20 | TRPS1 |
| SPINK1 | CD93 | ANKRD13D | CTNNA2 | FAM133A | GRB2 | PSMC3IP | NOL3 | SEMA3A |
| PPBP | ABCD1 | TP53RK | B3GALNT1 | CDH7 | DCAF4L2 | MS4A4A | ISL2 | PAFAH1B3 |
| METTL11B | TESMIN | S100A12 | USP35 | ACSL3 | BRINP2 | HOXD1 | FANCD2 | KNTC1 |
| HILPDA | RASA3 | ODC1 | SLC11A1 | THBS4 | XCL2 | EFNA4 | CENPH | FXYD6 |
| DNASE1L2 | NDUFA6 | KIF1A | PEAR1 | RCOR3 | SLC9C1 | CCN2 | ADCK5 | COLQ |
| CALCB | IL1B | FLVCR2 | LMNB1 | NFATC3 | PLAG1 | STXBP6 | TLR9 | ARHGAP8 |
| ZNF587 | EXOSC9 | CLIC6 | GJD2 | IMP3 | MAFB | PSMB8 | RGS2 | TRPM8 |
| SPINDOC | CD9 | ANKRD10 | CTLA4 | FAM124B | GRB10 | MS4A3 | NOL11 | SEM1 |
| PPAT | ABCC5 | TP53INP1 | B2M | CDH5 | DCAF4L1 | HOXC13 | ISG20 | PADI4 |
| METRNL | TESC | S100A1 | USP32 | ACSL1 | BRINP1 | EFNA1 | FANCB | KNOP1 |
| HIGD2B | RASA1 | OCSTAMP | SLBP | THBS2 | XCL1 | CCL8 | CENPF | FXYD3 |
| DNASE1 | NDUFA12 | KIF18B | PDZD11 | RCOR2 | SLC9A5 | STX4 | ADCK1 | COLEC12 |
| CALCA | IL1A | FLT4 | LMF2 | NFATC2IP | PLAC9 | PSMB4 | TLR8 | ARHGAP29 |
| ZNF563 | EXOSC8 | CLIC2 | GJB7 | ILF2 | MAFA | MS4A2 | RGS17 | TRPM6 |
| SPIB | CD8B | ANKLE1 | CTCFL | FAM117B | GRAP2 | HOXC12 | NODAL | SELPLG |
| PPARGC1A | ABCC4 | TP53I13 | AZU1 | CDH4 | DCAF15 | EFEMP2 | ISG15 | PADI3 |
| MET | TERT | RYR1 | USP26 | ACRV1 | BRF2 | CCL7 | FANCA | KNL1 |
| HIGD2A | RARRES2 | OCIAD2 | SLAMF9 | THBS1 | XBP1 | STX1A | CENPE | FUT9 |
| DNAJC9 | NDRG1 | KIF18A | PDXK | RCN3 | SLC7A8 | PSMA7 | ADAT3 | COLCA2 |
| CALB2 | IL18RAP | FLT3LG | LMAN2L | NFATC1 | PLAC8L1 | MS4A1 | TLR7 | ARHGAP26 |
| ZNF560 | EXOSC7 | CLECL1 | GJB4 | IL9R | MAD2L2 | HOXB9 | RGS16 | TRPM4 |
| SPG21 | CD8A | ANKFN1 | CTBS | FAM111B | GPX2 | EFEMP1 | NOD2 | SELP |
| PPARG | ABCC11 | TOX4 | AXL | CDH24 | DCAF13 | CCL5 | ISCU | PACSIN1 |
| MEST | TEP1 | RXRG | USP18 | ACP6 | BRDT | STX18 | FAM9C | KMT2A |
| HIGD1A | RARA | OAS3 | SLAMF8 | THBD | WWP1 | PSMA6 | CENPA | FUT8 |
| DNAJC4 | NDFIP2 | KIF15 | PDX1 | RCC2 | SLC7A7 | MRPS30 | ADAT2 | COL9A2 |
| CADPS | IL18R1 | FLI1 | LMAN2 | NFAT5 | PLA2G7 | HOXB5 | TLR2 | ARHGAP25 |
| ZNF552 | EXOSC6 | CLEC9A | GJB1 | IL9 | MAD2L1 | EFCAB13 | RGS13 | TRPM2 |
| SPERT | CD86 | ANK1 | CTAG2 | DUSP12 | GPT2 | CCL4L2 | NNT | SELL |
| PPA1 | ABCC1 | TOX | AXIN2 | CCDC146 | DCAF10 | STX16 | IRX6 | PACRG |
| MESP2 | TENM1 | RUVBL2 | USO1 | ST6GALNAC4 | BRCA2 | PSKH1 | FAM90A1 | KLRK1 |
| HIC2 | RAPGEF6 | OAS2 | SLAMF1 | PRKAG2 | WRAP73 | MRPS21 | CEMP1 | FUT5 |
| DNAJC12 | NDC80 | KIF14 | PDRG1 | MMP9 | SLC7A6 | HOXB13 | ADARB1 | COL9A1 |
| CADM1 | IL18 | FLAD1 | LKAAEAR1 | HLA-DMA | PLA2G4F | EDRF1 | TLR10 | ARHGAP22 |
| ZNF541 | EXOSC5 | CLEC7A | GJA3 | DUS4L | MACC1 | CCL4 | RGS11 | TROAP |
| SPDYE8P | CD84 | ANGPTL2 | CTAG1B | CCDC134 | GPSM1 | STX10 | NMUR2 | SELE |
| POU6F2 | ABCB9 | TOPBP1 | AVPR1B | ST6GALNAC2 | DBX1 | PSG2 | IRX5 | PABPC1L2B |
| MESP1 | TEKT5 | RUNX3 | USF1 | PRIM1 | BRCA1 | MRPS17 | FAM78B | KLRG1 |
| HIC1 | RAP1GAP | OAS1 | SLA | MMP25 | WNT7B | HOXA9 | CEMIP | FUT4 |
| DNAJC10 | NCR3 | KIF13B | PDPN | HLA-C | SLC7A5 | EDN1 | ADAR | COL8A2 |
| CACYBP | IL17REL | FKBP6 | LITAF | DUS1L | PLA2G4C | CCL3L1 | TLR1 | ARHGAP19 |
| ZNF528 | EXOC6 | CLEC5A | GIPR | CCDC117 | MAB21L3 | STRA8 | RGS1 | TRMT12 |
| SPDYE6 | CD80 | ANGPT4 | CSTL1 | ST3GAL6 | GPRIN1 | PSAT1 | NMRAL1 | SEC61G |
| POU5F1B | ABCB5 | TOP2A | AURKB | PRG2 | DBNDD1 | MRPL55 | IRX3 | PABPC1L2A |
| MEP1A | TEKT2 | RUNX2 | USB1 | MMP24 | BRAP | HOXA4 | FAM72D | KLRF1 |
| HIBCH | RANGRF | NXPH4 | SKP2 | HLA-B | WNT7A | EDARADD | CELSR3 | FUT1 |
| DNAJB13 | NCR1 | KIF13A | PDP1 | DTYMK | SLC7A11 | CCL26 | ADAP2 | COL6A3 |
| CACNG7 | IL17A | FKBP4 | LIPA | CCDC102B | PLA2G4A | STRA6 | TLL2 | ARHGAP15 |
| ZNF521 | EXOC2 | CLEC4C | GINS4 | ST3GAL5 | M1AP | PSAP | RGPD5 | TRMO |
| SPDYC | CD79B | ANGPT2 | CSTA | PRF1 | GPRC5B | MRPL52 | NME8 | SEC31B |
| POU4F3 | ABCB4 | TOP1MT | AURKA | MMP2 | DBF4 | HOXA3 | IRGC | PABPC1L |
| MELTF | TEK | RUBCNL | UQCRFS1 | HLA-A | BRAF | EDAR | FAM72B | KLRD1 |
| HHIPL2 | RANBP17 | NXF5 | SKP1 | DTX4 | WNT5B | CCL23 | CELF5 | FURIN |
| DNAJB1 | NCOA4 | KIF11 | PDLIM4 | CC2D2B | SLC7A10 | STPG4 | ADAMTSL1 | COL6A2 |
| CACNG4 | IL16 | FKBP1B | LINGO3 | ST3GAL4 | PLA2G2D | PRX | TLE5 | ARHGAP11A |
| ZNF492 | EXO5 | CLEC4A | GINS2 | PREP | LYZ | MRPL47 | RGMB | TRIT1 |
| SPDYA | CD79A | ANGEL1 | CST7 | MMP16 | GPR89B | HOXA2 | NME3 | SEC14L1 |
| POU4F1 | ABCA13 | TONSL | AUNIP | HK3 | DAZAP2 | EDA2R | IRF9 | PABPC1 |
| MELK | TEDC2 | RTTN | UQCRC1 | DTX1 | BPIFA2 | CCL22 | FAM72A | KLRC4- |
| HHIP | RANBP1 | NXF2 | SKAP2 | CBX8 | WNT10B | STPG3 | CELF4 | FUCA1 |
| DNAH2 | NCKAP1L | KIAA1549L | PDK3 | ST3GAL1 | SLC6A4 | PRSS50 | ADAMTS3 | COL6A1 |
| CACNA1D | IL12RB2 | FITM2 | LINGO1 | PRELP | PLA1A | MRPL4 | TLE3 | ARHGAP10 |
| ZNF488 | EXO1 | CLEC2L | GINS1 | MMP15 | LYVE1 | HOXA1 | RGL3 | TRIP13 |
| SPDEF | CD74 | ANAPC13 | CST1 | HJURP | GPR65 | ECT2 | NMBR | SDK1 |
| POU3F2 | AARD | TOMM40L | ATP9A | DTNB | DAXX | CCL21 | IRF8 | P4HB |
| MEIOC | TEDC1 | RTP2 | UQCRB | CBX6 | BPI | STPG2 | FAM71F2 | KLRC4 |
| HHEX | RAMP3 | NVL | SKAP1 | ST18 | WIPI2 | PRSS41 | CELF3 | FTSJ3 |
| DNAH17 | NCF2 | KIAA1328 | PDK1 | PRELID3A | SLC6A3 | MRPL24 | ADAMTS20 | COL5A3 |
| CACNA1B | IL12B | FIGN | LIN9 | MMP12 | PKP4 | HORMAD1 | TLCD1 | ARFGEF3 |
| ZNF48 | EWSR1 | CLEC2D | GIMAP5 | HIVEP2 | LYST | ECSCR | RGL2 | TRIP12 |
| SPCS3 | CD72 | ANAPC11 | CSRP2 | DTL | GPR61 | CCL20 | NMB | SDCBP |
| POU2F2 | A4GNT | TNK2 | ATP8B4 | CBX3 | DARS | STON2 | IRF7 | P4HA1 |
| MEGF9 | TECPR2 | RTL9 | UQCC3 | SSX5 | BPGM | PRSS23 | FAM71E2 | KLRC3 |
| HGH1 | RAMP2 | NUTM1 | SKA3 | PRDM4 | WIPF1 | MROH7 | CEL | FTL |
| DNAH14 | NCF1 | KIAA1324L | PDIA4 | MMEL1 | SLC52A2 | HOMER2 | ADAMTS2 | COL5A2 |
| CABYR | IL12A | FIBIN | LIN7A | HIST4H4 | PKMYT1 | ECE2 | TKTL1 | AQP9 |
| ZNF469 | EVI5 | CLEC2B | GIMAP1- | DTD1 | LYPD6B | CCL2 | RGCC | TRIM9 |
| SPC25 | CD70 | AMZ1 | CSRP1 | CBX2 | GPR45 | STMN1 | NLRP8 | SCX |
| POU2AF1 | TEC | TNIP3 | ATP8A2 | SSX1 | DAPP1 | PRSS21 | IRF4 | P2RY2 |
| MEGF8 | RALGPS2 | RTL1 | UPK3A | PRDM13 | BORA | MRGPRE | FAM71E1 | KLRC1 |
| HESX1 | NCBP2 | NUSAP1 | SKA1 | MME | WFIKKN1 | HNRNPM | CEACAM8 | FSTL5 |
| DNAH11 | IL10RB | KIAA1324 | PDGFRL | HIST3H2BB | SLC4A8 | ECE1 | ADAMTS18 | COL4A4 |
| CABP7 | EVI2B | FHL2 | LIN28B | DSN1 | PKM | CCL19 | TK1 | AQP3 |
| ZNF468 | CD7 | CLEC1A | GGT5 | CBWD6 | LYG2 | STK32A | RFXANK | TRIM74 |
| SPC24 | TEAD2 | AMT | CSPG4 | SSUH2 | GPR4 | PRRX2 | NLRP3 | SCUBE2 |
| POP5 | RALGAPA2 | TNFSF9 | ATP7A | PRCP | DAPK2 | MRGBP | IRF1 | P2RY14 |
| MEFV | NCAPH | RTKN2 | UPK2 | MMD | BOP1 | HNRNPLL | FAM71D | KLRB1 |
| HES7 | IKZF1 | NUP85 | SIX4 | HIST3H2A | WFDC5 | EBI3 | CEACAM6 | FSTL1 |
| DNAAF3 | EVI2A | KIAA1257 | PDGFRB | DSCC1 | SLC45A3 | CCL18 | ADAMTS17 | COL4A1 |
| CABP4 | CD69 | FGR | LIN28A | CBWD3 | PKD2L2 | STK31 | TIPIN | AQP12A |
| ZNF467 | TDRD6 | CLEC17A | GGT1 | SSU72 | LYG1 | PRRX1 | RFX6 | TRIM67 |
| SPATA9 | RALB | AMPD2 | CSNK2A1 | PRC1 | GPR27 | MRC2 | NLRP2 | SCRT1 |
| POP4 | NCAPG2 | TNFSF8 | ATP6V1H | MLPH | DAPK1 | HNRNPH1 | IRAK3 | P2RY13 |
| MEF2C | IKBKG | RTEL1 | UPK1B | HIST2H3D | BOLA2 | EBF2 | FAM50A | KLK5 |
| HES6 | ETV4 | NUP37 | SIX3 | DSC1 | WFDC10A | CCL17 | CEACAM3 | FSIP1 |
| DNA2 | CD68 | KIAA0319 | PDGFRA | CBLN1 | SLC45A2 | STIM1 | ADAMDEC1 | COL3A1 |
| CABLES2 | TDRD5 | FGL2 | LIMS3 | SSPO | PJVK | PRR7 | TIPARP | APP |
| ZNF443 | RAI14 | CLEC16A | GGH | PRAP1 | LY96 | MRC1 | RFC5 | TRIM59 |
| SPATA25 | NCAPG | AMPD1 | CSMD3 | MLANA | GPR26 | HNRNPD | NLRP12 | SCNN1D |
| POMP | IKBIP | TNFSF4 | ATP6V1F | HIST2H3C | DAP3 | EBF1 | IQGAP3 | P2RY10 |
| MED7 | ETV3 | RTBDN | UNC79 | DRICH1 | BMX | CCL14 | FAM49A | KLK2 |
| HES4 | CD63 | NUP210L | SIX1 | CBLB | WDR97 | STIL | CEACAM19 | FSD1 |
| DMTN | TDRD1 | KHDC4 | PDGFA | SS18L2 | SLC44A5 | PRR5L | ADAM8 | COL2A1 |
| CA9 | RAE1 | FGGY | LIMS1 | PRAMEF4 | PJA2 | MPZL1 | TINAGL1 | APOO |
| ZNF442 | NCAPD2 | CLEC14A | GGCT | MKRN3 | LY9 | HNMT | RFC4 | TRIM37 |
| SPATA12 | IGSF8 | AMOTL2 | CSMD2 | HIST2H2AC | GPR25 | EAF2 | NLRP11 | SCNN1A |
| POLR3K | ETV1 | TNFSF18 | ATP6V1D | DRC7 | DAGLB | CCL13 | IQCB1 | P2RX5 |
| MED13 | CD6 | RSRC1 | UNC5D | CBARP | BMPR1B | STEAP4 | FAM47C | KLK15 |
| HES1 | TCTN3 | NUP210 | SIT1 | SRXN1 | WDR91 | PRR5 | CDT1 | FSCN1 |
| DMRTC2 | RADX | KHDC1L | PDF | PRAME | SLC44A2 | MPV17L2 | ADAM7 | COL26A1 |
| CA8 | NCAM2 | FGFR4 | LIMK1 | MKI67 | PIWIL4 | HNF1B | TIMM8A | APOLD1 |
| ZNF439 | IGSF6 | CLEC10A | GFRA3 | HIST1H4J | LY86 | E2F7 | RFC3 | TRIM36 |
| SPAG4 | ETS1 | AMIGO3 | CSF3R | DRAXIN | GPR19 | CCL1 | NLGN1 | SCNM1 |
| POLR2J2 | CD59 | TNFSF15 | ATP6V1C2 | CAVIN2 | DACH1 | STEAP1 | IPO11 | P2RX1 |
| MED12L | TCTN2 | RSPH14 | UNC5A | SRSF7 | BMP6 | PRR19 | FAM43A | KLK1 |
| HERC6 | RAD9A | NUP155 | SIRPG | PRAC2 | WDR87 | MPV17 | CDKN3 | FRY |
| DMRTA2 | NCALD | KEL | PDE7B | MITF | SLC39A6 | HMSD | ADAM33 | COL23A1 |
| CA4 | IGLON5 | FGFBP2 | LIME1 | HIST1H4I | PIWIL3 | E2F5 | TIMM50 | APOL6 |
| ZNF432 | ESRRA | CLDN9 | GFRA1 | DPYSL5 | LY6G5C | CCKBR | REXO2 | TRIM24 |
| SP7 | CD58 | AMH | CSF2RA | CAV1 | GPR183 | STC2 | NKG7 | SCN9A |
| POLR2I | TCP11 | TNFSF14 | ATP6V1B2 | SRSF6 | DAB2IP | PRR16 | IPCEF1 | OXGR1 |
| MED10 | RAD54L | RSAD2 | UNC13A | PQBP1 | BMP2K | MPP6 | FAM3C | KLHL7 |
| HERC5 | NBPF6 | NUP107 | SIRPB1 | MIS18A | WDR86 | HMMR | CDKN2AIP | FRMPD4 |
| DMRT2 | IGLL5 | KDR | PDE6C | HIST1H4H | SLC39A14 | E2F3 | ADAM28 | COL1A2 |
| CA12 | ESM1 | FGF9 | LIMA1 | DPYD | PIWIL2 | CCDC91 | TIMM13 | APOL3 |
| ZNF395 | CD55 | CLDN5 | GFPT2 | CATSPERG | LUZP2 | STAT5B | RETREG1 | TRIM17 |
| SP6 | TCL1B | AMDHD2 | CSF2 | SRSF4 | GPR18 | PRR11 | NKD1 | SCN3A |
| POLR2H | RAD54B | TNFSF13 | ATP6V1B1 | PPP4R3C | DAB2 | MPP1 | INTS8 | OXCT1 |
| MED1 | NBPF4 | RRP9 | UMODL1 | MIPEP | BMI1 | HMGB3 | FAM3B | KLHL4 |
| HEPHL1 | IGHMBP2 | NUF2 | SIRPA | HIST1H4E | WDR83 | E2F1 | CDKN2A | FRMPD2 |
| DMRT1 | ESCO2 | KDM6B | PDE4B | DPY19L1 | SLC38A8 | CCDC88A | ADAM23 | COL1A1 |
| C9orf50 | CD53 | FGF7 | LILRB5 | CAT | PISD | STAT5A | TIMELESS | APOE |
| ZNF324 | TCL1A | CLDN25 | GFOD2 | SRSF12 | LUM | PRPF4 | RETNLB | TRIM16L |
| SP140 | RAD51AP1 | AMACR | CSF1R | PPP4R3A | GPR179 | MPO | NKAIN3 | SCIN |
| POLQ | NBPF15 | TNFSF10 | ATP6V1A | MINPP1 | DAB1 | HMGB2 | INTS7 | OTX2 |
| ME3 | IGFLR1 | RRP12 | ULK1 | HIST1H4D | BLVRB | DYTN | FAM24B | KLHL35 |
| HEPACAM2 | ESAM | NUDT9 | SIK1 | DPT | WDR76 | CCDC80 | CDKAL1 | FRMD8 |
| DMC1 | CD52 | KDM4C | PDE2A | CASQ1 | SLC38A1 | STAT4 | ADAM21 | COL14A1 |
| C9orf116 | TCF7 | FGF3 | LILRB4 | SRSF1 | PIR | PROSER1 | TIMD4 | APOD |
| ZNF322 | RAD21 | CLDN20 | GFOD1 | PPP1R3C | LUC7L3 | MPL | RET | TRIM11 |
| SOX9 | NAXE | ALPL | CSF1 | MIF4GD | GPR174 | HMGA1 | NKAIN2 | SCHIP1 |
| POLN | IGFBP6 | TNFRSF9 | ATP6V0D2 | HIST1H4C | CYTH1 | DYSF | INTS4 | OTUD7A |
| ME1 | ERP27 | RRM2 | ULBP2 | DPP7 | BLOC1S3 | CCDC77 | FAM234B | KLHL3 |
| HENMT1 | CD5 | NUDT8 | SIGLEC9 | CASP8 | WDR66 | STAT3 | CDK6 | FRMD4A |
| DMBX1 | TCF19 | KDM1A | PDE1C | SRRM3 | SLC35G6 | PROK2 | ADAM15 | COL12A1 |
| C8orf76 | RACGAP1 | FGF18 | LILRB2 | PPP1R35 | PIP5KL1 | MPG | TIGIT | APOC1 |
| ZNF320 | NAV3 | CLDN11 | GFM1 | MIF | LTO1 | HMG20B | REPS2 | TRIB2 |
| SOX7 | IGFBP5 | ALPK3 | CSE1L | HIST1H4B | GPR171 | DYNLT1 | NIPSNAP3B | SCGN |
| POLE2 | ERICH5 | TNFRSF8 | ATP6V0B | DPP4 | CYSLTR2 | CCDC74A | INSYN2A | OTP |
| MDM2 | CD48 | RRM1 | ULBP1 | CASP5 | BLNK | STAT1 | FAM222A | KLHL26 |
| HELLS | TCF15 | NUDT16 | SIGLEC8 |  | WDR53 | PRODH | CDK5RAP3 | FRMD3 |
| DLX6 | RAC3 | KCTD6 | PDCD5 |  | SLC35E3 | MPC2 | ADAM12 | COL11A2 |
| C7orf61 | NAV1 | FGF17 | LILRA6 |  | PIP5K1A | HLA-G | TIGD5 | APOBEC3G |
| ZNF30 | IGFBP3 | CLCNKB | GFI1 |  | LTK | DYNC2LI1 | REPS1 | TRHDE |
| SOX5 | ERI1 | ALOXE3 | CSAG3 |  | GPR153 | CCDC57 | NIPSNAP3A | SCGB1D2 |
| POLD1 | CD47 | TNFRSF6B | ATP6AP2 |  | CYREN | STARD3 | INSYN1 | OSM |
| MDK | TBXT | RRAGD | UHRF1 |  | BLM | PROCA1 | FAM189B | KLHL21 |
| HECW2 | RABEP2 | NUDT1 | SIGLEC6 |  | WDR48 | MON1B | CDK5RAP2 | FRK |
| DLX5 | NATD1 | KCTD19 | PDCD2L |  | SLC35D3 | HLA-DRB1 | ACVRL1 | COCH |
| C6orf52 | IGF2BP3 | FGF12 | LILRA5 |  | PIP4P1 | DVL3 | TIGD3 | APOBEC3A |
| ZNF292 | ERG | CLCN7 | GEMIN6 |  | LTC4S | CCDC34 | RENBP | TREML4 |
| SOX30 | CD40LG | ALOX5AP | CSAG2 |  | GPR152 | STAP1 | NINJ1 | SCG5 |
| POLA2 | TBXAS1 | TNFRSF4 | ATP6AP1 |  | CYP4Z1 | PRMT1 | INSR | OSER1 |
| MDH1 | RAB6B | RPUSD1 | UGT2B17 |  | BLK | MOCS3 | FAM189A1 | KLHL17 |
| HEATR6 | NAT10 | NUDCD1 | SIGLEC5 |  | WDR34 | HLA-DRA | CDK5R1 | FRAT2 |
| DLX4 | IGF2BP2 | KCP | PDCD1LG2 |  | SLC35B3 | DUT | ACVR1C | COA6 |
| C6orf223 | ERCC6L | FGD5 | LILRA4 |  | PIMREG | CCDC28B | TIE1 | APLP2 |
| ZNF286A | CD40 | CLCN5 | GEMIN2 |  | LTBP2 | STAG3 | REN | TREML2 |
| SOX2 | TBXA2R | ALOX5 | CSAG1 |  | GPR15 | PRLR | NID2 | SCG3 |
| POGLUT2 | RAB42 | TNFRSF25 | ATP5MG |  | CYP4F3 | MOCOS | INSM1 | OSBPL3 |
| MDGA2 | NAT1 | RPS9 | UGT1A8 |  | BIVM | HLA-DQB1 | FAM186B | KLHL14 |
| HEATR4 | IGF2BP1 | NUCB2 | SIGLEC15 |  | WDHD1 | DUSP6 | CDK5 | FPR3 |
| DLX2 | ERC2 | KCNV1 | PDCD1 |  | SLC31A2 | CCDC183 | ACTR3B | CNTNAP5 |
| C6orf15 | CD4 | FGD4 | LILRA2 |  | PIM3 | STAC | TICRR | APLP1 |
| ZNF280A | TBX6 | CLC | GDPD5 |  | LTB | PRKY | REM2 | TREML1 |
| SOX18 | RAB40B | ALOX15 | CRYGN |  | GPR146 | MOB1B | NIBAN3 | SCG2 |
| POGK | NARF | TNFRSF1B | ATP5ME |  | CYP4F2 | HLA-DQA2 | INPPL1 | OSBPL1A |
| MCOLN3 | IGF2 | RPS7 | UFSP1 |  | BIRC7 | DUSP5 | FAM184B | KLHDC4 |
| HDC | ERBB3 | NTS | SIGLEC14 |  | WASHC5 | CCDC18 | CDK2 | FPR2 |
| DLL4 | CD3G | KCNU1 | PCSK9 |  | SLC30A8 | STAB1 | ACTR3 | CNTNAP4 |
| C5orf34 | TBX21 | FFAR2 | LILRA1 |  | PIM2 | PRKRIP1 | THY1 | APLN |
| ZNF239 | RAB3C | CKS2 | GDPD4 |  | LTA | MNDA | RELN | TREM2 |
| SOX17 | NANOS3 | ALKBH6 | CRYBB1 |  | GPR143 | HLA-DQA1 | NHSL2 | SCARF2 |
| POFUT1 | IGF1R | TNFRSF1A | ATP5F1B |  | CYP2W1 | DUSP23 | INPP5F | OSBPL10 |
| MCM8 | ERBB2 | RPS6KL1 | UCN |  | BIRC5 | CCDC177 | FAM178B | KLHDC2 |
| HCN4 | CD3E | NTRK1 | SIGLEC10 |  | WASF1 | ST8SIA5 | CDK18 | FPR1 |
| DLK1 | TBX2 | KCNT2 | PCSK5 |  | SLC30A5 | PRKCQ | ACTN4 | CNTNAP2 |
| C5AR2 | RAB3B | FEZ1 | LIG1 |  | PILRB | MND1 | THUMPD2 | APCDD1 |
| ZNF222 | NADSYN1 | CKS1B | GDPD1 |  | LST1 | HLA-DPB1 | RELL2 | TREM1 |
| SOX12 | IGF1 | ALKBH3 | CRYBA4 |  | GPR139 | DUSP2 | NHLRC1 | SCARB2 |
| PODXL2 | EQTN | TNFRSF18 | ATP5F1A |  | CYP2S1 | CCDC167 | INPP4B | OSBP2 |
| MCM7 | CD3D | RPS6KC1 | UCKL1 |  | BIRC3 | ST8SIA4 | FAM174B | KLF9 |
| HCN3 | TBRG4 | NTN3 | SIGLEC1 |  | WARS | PRKCH | CDK16 | FPGS |
| DLGAP5 | RAB3A | KCNQ3 | PCSK2 |  | SLC30A3 | MMS22L | ACTL8 | CNTN3 |
| C5AR1 | NACC1 | FEV | LHX9 |  | PILRA | HLA-DPA1 | THUMPD1 | APBB2 |
| ZNF22 | IGDCC3 | CKAP5 | GDF9 |  | LSP1 | DUSP15 | RELA | TRAT1 |
| SOX11 | EPYC | ALG8 | CRYAB |  | GPR137C | CCDC154 | NHLH1 | SCARA3 |
| PODXL | CD38 | TNFRSF17 | ATP1B3 |  | CYP27C1 | ST8SIA2 | INO80C | ORM1 |
| MCM6 | TBCD | RPS26 | UCHL1 |  | BIRC2 | PRKCG | FAM171A2 | KLF5 |
| HCN2 | RAB39B | NTN1 | SIAH1 |  | VWF | MMRN2 | CDK12 | FOXRED2 |
| DLGAP3 | NAB1 | KCNN2 | PCP2 |  | SLC2A6 | HLA-DOB | ACTL6A | CNR2 |
| C3orf18 | IFRD1 | FES | LHFPL4 |  | PIK3R3 | DUSP14 | THSD7B | APBA2 |
| ZNF217 | EPS8 | CKAP4 | GDF6 |  | LSM7 | CCDC151 | REG4 | TRAPPC5 |
| SOX10 | CD37 | ALG1L | CRY2 |  | GPR1 | ST8SIA1 | NFKBIA | SCAMP5 |
| PODN | TBCC | TNFRSF13B | ATP1A3 |  | CYP27B1 | PRKCD | INKA2 | ORC6 |
| MCM5 | RAB38 | RPS24 | UBL3 |  | BIN2 | MMRN1 | FAM170A | KLF12 |
| HCLS1 | NAALADL2 | NT5M | SHROOM4 |  | VWA5B2 | HLA-DMB | CDK10 | FOXP3 |
| DLEU7 | IFNG | KCNN1 | PCOLCE2 |  | SNCA | TAF4 | AHCYL1 | CPT1B |
| C3AR1 | EPOR | FERMT1 | LHCGR |  | PLPP2 | PTPRB | TMEM255A | ASF1B |
| ZNF213 | CD36 | CKAP2L | GDF15 |  | MAPK15 | MYBPC3 | RNF125 | TUBB8 |
| SOSTDC1 | TBCB | ALG11 | CRX |  | GZMA | HVCN1 | NR0B1 | SFXN1 |
| POC1B | RAB30 | TNFRSF11B | ATP1A1 |  | DENND5B | EME1 | KAAG1 | PCDHA1 |
| MCM4 | NAALADL1 | RPS21 | UBE2T |  | C19orf25 | CD163 | FCAR | LAPTM5 |
| HCK | IFNA10 | NT5DC3 | SHOX2 |  | ZC3H7A | TAF2 | CHAF1B | GALNT11 |
| DLC1 | EPOP | KCNMB4 | PCOLCE |  | SNAP47 | PTPN7 | AGPAT5 | CPSF4L |
| C3 | CD34 | FEN1 | LGSN |  | PLP1 | MYBL2 | TMEM223 | ASCL4 |
| ZNF205 | TBC1D9 | CKAP2 | GDF1 |  | MAP9 | HUS1B | RNF113B | TUBB4A |
| SORL1 | RAB27B | ALDOA | CRTC3 |  | GYPE | EMCN | NQO1 | SFN |
| POC1A | NAAA | TNFRSF11A | ATP13A1 |  | DENND1C | CD160 | JUP | PCDH19 |
| MCM3AP | IFITM2 | RPS2 | UBE2S |  | C17orf99 | TAF15 | FBXW9 | LAPTM4B |
| HCFC1R1 | EPN2 | NT5DC2 | SHOC1 |  | ZC3H3 | PTPN6 | CHAF1A | GALC |
| DISP3 | CD33 | KCNK9 | PCNX4 |  | SMYD2 | MYBL1 | AGO2 | CPSF1 |
| C2CD6 | TBC1D5 | FDX1 | LGMN |  | PLOD3 | HUNK | TMEM222 | ASCL2 |
| ZNF165 | RAB20 | CITED4 | GDE1 |  | MAP7D2 | ELP3 | RNASEH2A | TUBB3 |
| SORCS3 | NAA38 | ALDH9A1 | CRTC2 |  | GUSB | CD151 | NPTX2 | SF3B4 |
| PNPLA8 | IFIT5 | TNFRSF10C | ATP10D |  | DENND1B | TACSTD2 | JTB | PCDH18 |
| MCM3 | EPHX4 | RPRML | UBE2QL1 |  | C17orf64 | PTPN20 | FBXO5 | LAP3 |
| HCAR1 | CD320 | NT5C1B | SHLD1 |  | ZBTB8B | MYB | CHAD | GAL3ST4 |
| DISC1 | TBC1D4 | KCNK5 | PCNX2 |  | SMS | HTR2C | AGAP6 | CPNE9 |
| C2CD4D | RAB1A | FDPS | LGALS9 |  | PLLP | ELP2 | TMEM213 | ASCL1 |
| ZNF135 | NAA20 | CIT | GDAP1 |  | MAP4K2 | CD14 | RNASE6 | TUBB2B |
| SORBS2 | IFIT3 | ALDH3A2 | CRTAM |  | GUCY2D | TACC3 | NPR1 | SF1 |
| PNPLA7 | EPHB6 | TNFAIP6 | ATM |  | DEGS2 | PTPN11 | JPH3 | PBXIP1 |
| MCM2 | CD302 | RPP38 | UBE2Q1 |  | C17orf53 | MXRA8 | FBXO47 | LAMTOR2 |
| HBEGF | TBC1D3H | NSUN7 | SHISA7 |  | ZBTB41 | HTR2B | CH25H | GAL3ST1 |
| DIRAS2 | RAB15 | KCNJ3 | PCNX1 |  | SMPDL3B | ELOVL7 | AGAP5 | CPNE7 |
| C21orf58 | NAA10 | FDFT1 | LGALS1 |  | PLK4 | CD109 | TMEM211 | ASB8 |
| ZNF117 | IFIT2 | CIPC | GDA |  | MAP4K1 | TAC4 | RNASE3 | TUBB |
| SOD1 | EPHB4 | ALDH1B1 | CRPPA |  | GUCA1A | PTH2R | NPPA | SEZ6L2 |
| PNPLA6 | CD300LF | TNFAIP3 | ATL2 |  | DEFA4 | MXD3 | JMJD4 | PBX4 |
| MCM10 | TBC1D3C | RPP21 | UBE2L6 |  | C12orf75 | HSPG2 | FBXO43 | LAMP5 |
| HAVCR2 | RAB11FIP4 | NSUN5 | SHE |  | ZBTB32 | ELOC | CGB5 | GAGE2A |
| DIRAS1 | MZT2A | KCNJ15 | PCNA |  | SMPD3 | CD101 | AGAP4 | CPNE5 |
| C20orf204 | IFIT1 | FCSK | LFNG |  | PLK1 | TAC3 | TMEM205 | ASB2 |
| ZMIZ2 | EPHB3 | CIP2A | GCSH |  | MAP3K13 | PTGS2 | RNASE2 | TUBA3E |
| SOCS1 | CD300LB | ALDH1A2 | CRISPLD2 |  | GTSF1 | MXD1 | NPL | SEZ6L |
| PNOC | TBC1D3B | TNFAIP2 | ATIC |  | DEDD | HSPD1 | JCHAIN | PBX3 |
| MCF2L2 | RAB11FIP1 | RPL39L | UBE2F |  | C12orf45 | ELOB | FBXO41 | LAMP3 |
| HAVCR1 | MZT1 | NSMF | SHCBP1 |  | ZBTB18 | CCZ1B | CFTR | GAGE12J |
| DIPK2B | IFIH1 | KCNJ11 | PCM1 |  | SMN1 | SYTL5 | AFF3 | CPNE2 |
| C1S | EPHB2 | FCRLB | LEPROTL1 |  | PLIN5 | PTGS1 | TMEM198 | ASB16 |
| ZIC5 | CD300E | CIAO3 | GCNT1 |  | C1orf54 | ICAM3 | FCGR3A | LCN10 |
| SNX9 | TBC1D31 | ALCAM | CRISPLD1 |  | ZFP36L1 | ENGASE | CHL1 | GAS2L3 |
| PNMA3 | QTRT1 | TNFAIP1 | ATG7 |  | SNRPA1 | CD200 | AIF1 | CREB3L1 |
| MCCC2 | MZB1 | RPL21 | UBE2D2 |  | PLXNC1 | TAL1 | TMEM63C | ASPHD1 |
| HAUS8 | IFI6 | NSMCE2 | SHC1 |  | MARCO | PUF60 | RNF19A | TYMP |
| DIPK1B | EPHA8 | KCNIP1 | PCLAF |  | H2AFY | MYH4 | NR5A1 | SGPL1 |
| C1R | CD300A | FCRLA | LEF1 |  | DGKA | ICAM2 | KBTBD12 | PCDHB11 |
| ZIC4 | TBC1D3 | CHTF18 | GCK |  | C1orf162 | ENG | FCGR2B | LCK |
| SNX31 | QRSL1 | AKT3 | CRISP3 |  | ZEB2 | CD2 | CHIT1 | GAPDH |
| PNMA2 | MYT1 | TMSB15A | ATG4D |  | SNRNP40 | TAGLN3 | AICDA | CREB1 |
| MC1R | IFI44L | RPL10L | UBE2C |  | PLXNA3 | PTX4 | TMEM38A | ASPH |
| HAUS6 | EPHA1 | NSMCE1 | SHARPIN |  | MARCKSL1 | MYEOV | RNF185 | TYK2 |
| DIAPH3 | CD2BP2 | KCNH8 | PCGF2 |  | H2AFX | ICA1 | NR4A3 | SGO2 |
| C1QTNF6 | TBC1D26 | FCRL6 | LDLRAD3 |  | DGCR6 | ENC1 | KBTBD11 | PCDHA8 |
| ZIC3 | QRFPR | CHST7 | GBX2 |  | C1orf159 | CD1E | FCGR2A | LCE1C |
| SNX24 | MYRIP | AKR1E2 | CRIP3 |  | ZEB1 | TAGLN | CHI3L2 | GALR3 |
| PNLDC1 | IFI44 | TMPRSS9 | ATF7IP |  | SNRNP25 | PTRH2 | AHSA1 | CRACR2B |
| MBP | EPDR1 | RPIA | UBD |  | PLXNA2 | MYCT1 | TMEM33 | ASNS |
| HAUS3 | CD28 | NSD3 | SHANK3 |  | 3-Mar | IBSP | RNF183 | TXNRD2 |
| DHX58 | TBC1D24 | KCNH2 | PCGF1 |  | H2AFB1 | EN2 | NR4A2 | SGO1 |
| C1QTNF3 | QPCTL | FCRL5 | LDHA |  | DGAT2 | CD1D | KAZALD1 | PCDHA7 |
| ZIC2 | MYPOP | CHST2 | GBP3 |  | C1orf112 | TAGAP | FCGR1B | LCE1B |
| SNX22 | IFI35 | AKR1C3 | CRIP2 |  | ZDHHC9 | PTPRT | CHI3L1 | GALR1 |
| PNKP | EPB41 | TMPRSS3 | ATF4 |  | SNORC | MYCN | AHR | CR2 |
| MBOAT7 | CD274 | ROPN1B | UBASH3A |  | PLXNA1 | IARS | TMEM31 | ASIC1 |
| HASPIN | TBC1D14 | NRXN3 | SH3TC2 |  | MAPRE3 | EN1 | RNF175 | TXNRD1 |
| DHX34 | QPCT | KCNH1 | PCDHGC4 |  | GZMM | CD1C | NR2F6 | SGMS1 |
| C1QTNF12 | MYO7A | FCRL4 | LDB3 |  | DEPP1 | TAFA3 | KATNAL2 | PCDHA5 |
| ZIC1 | IFI30 | CHST15 | GBP2 |  | C1GALT1C1 | PTPRJ | FCGR1A | LBR |
| SNX13 | EOMES | AKR1C1 | CRHR2 |  | ZDHHC23 | MYCL | CHEK2 | GALNTL6 |
| PNCK | CD27 | TMIE | ATAD5 |  | SNN | HYLS1 | AHNAK | CPXM2 |
| MBLAC1 | TBC1D1 | ROMO1 | UBA52 |  | PLVAP | EMP3 | TMEM270 | ASH2L |
| HAPLN3 | QDPR | NRTN | SH3RF1 |  | MAPK8IP2 | CD1B | RNF144B | TXK |
| DHRS7 | MYO3A | KCNG3 | PCDHGB1 |  | GZMK | TAFA2 | NR2E3 | SGK3 |
| C1QL4 | IFI16 | FCRL3 | LDB2 |  | DEPDC5 | PTPRG | KAT6B | PCDHA4 |
| ZGRF1 | ENY2 | CHRNB4 | GBA |  | C19orf57 | MYCBP | FCER2 | LBH |
| SNURF | CD248 | AKR1B10 | CRH |  | ZCRB1 | HYDIN | CHEK1 | GALNT17 |
| PMPCB | TASOR2 | TMEM97 | ATAD3B |  | SNF8 | EMP1 | AHI1 | CPXM1 |
| MBL2 | PYHIN1 | ROBO4 | UBA2 |  | PLSCR1 | CD1A | TMEM259 | ASGR2 |
| HAL | MYO1H | NRP2 | SH3KBP1 |  | MAPK8IP1 | TAF7L | RNF141 | TUT7 |
| DHRS11 | IDO1 | KCNG2 | PCDHGA8 |  | GZMH | PTPRCAP | NR2E1 | SGCB |
| C1QL1 | ENTPD6 | FCRL2 | LCTL |  | DEPDC1B | MYC | KAT6A | PCDHA2 |
| ZGLP1 | CD247 | CHRNB2 | GATM |  | C19orf53 | HYAL3 | FCER1G | LAT |
| SNTG1 | TAS2R38 | AKNA | CREM |  | ZCCHC12 | EMID1 | CHD5 | GALNT16 |
| PMFBP1 | PYDC1 | TMEM9 | ATAD2 |  | SNCG | CD19 | AHCYL2 | CPVL |
| MB | MYO1G | RNPS1 | UBA1 |  | PLPP5 | TAF4B | TMEM255B | ASGR1 |
| HAGHL | IDH3A | NRIP3 | SH3D19 |  | MAPK7 | PTPRC | RNF128 | TUSC1 |
| DHFR | ENTPD1 | KCNG1 | PCDHB8 |  | GZMB | MYBPHL | NR2C2AP | SFXN3 |
| C1QC | CD244 | FCRL1 | LCT |  | DEPDC1 | HYAL2 | KANK2 | PCDHA11 |
| ZFYVE1 | TAS1R1 | CHRNA5 | GATD1 |  | C19orf48 | EME2 | FCER1A | LARS2 |
| SNRPN | PYCR3 | AKAP4 | CREG2 |  | ZC3HC1 | CD180 | CHD1L | GALNT14 |
| PMEPA1 | MYO1F | TMEM8B | ASRGL1 |  | SNRPD1 | ICOSLG | AIM2 | CREB5 |
| MATN2 | ID3 | RNFT2 | UAP1L1 |  | PMCH | ENO2 | TMEM74 | ASPRV1 |
| HADHA | ENPP3 | NRG4 | SH2D1B |  | MAST1 | CD209 | RNF208 | TYR |
| DGLUCY | CD226 | KCNF1 | PCDHB5 |  | H2BFM | TAP1 | NRG1 | SH2B1 |
| C1QB | TARDBP | FCN1 | LCP2 |  | DGKI | PVRIG | KCNB2 | PCDHB2 |
| ZFR2 | PYCR1 | CHRM5 | GATA3 |  | C1orf61 | MYL9 | FCGR3B | LCN12 |
| SNRPE | MYO15B | AK5 | CREBZF |  | ZFP36L2 | ICOS | CHML | GASK1B |
| PMEL | ID1 | TMEM81 | ASPSCR1 |  | SNRPC | ENKD1 | AIFM2 | CREB3L4 |
| MASTL | ENPP1 | RNF43 | TYROBP |  | PM20D1 | CD207 | TMEM65 | ASPM |
| H3F3A | CD22 | NRG2 | SH2D1A |  | MASP1 | TANGO2 | RNF207 | TYMS |
| DGKZ | TAPBP | KCNE3 | PCDHB3 |  | H2AFY2 | PUS1 | NRBP2 | SGSM1 |
| C1QA | PXK | FCMR | LCORL |  | DGKH | MYH7B | KCNA3 | PCDHB16 |
| ZFP69B | MYO10 | CHRM3 | GATA2 |  |  |  |  |  |
|  |  |  |  |  |  |  |  |  |

**Supplementary Table 2:** There were 1251 TME-associated genes (FDR<0.05 and |log2FC|>1) differentiated in expression between LUAD and normal lung tissues.

| gene | conMean | treatMean | logFC | pValue | fdr |
| --- | --- | --- | --- | --- | --- |
| SRPK1 | 4.363002 | 14.59816 | 1.742394 | 1.31E-35 | 4.47E-33 |
| SRMS | 0.224173 | 1.331281 | 2.57013 | 4.92E-13 | 1.03E-12 |
| PPP1R16A | 2.946937 | 6.163408 | 1.064512 | 2.24E-16 | 5.73E-16 |
| PPP1R14B | 12.96301 | 59.87535 | 2.207561 | 9.08E-33 | 1.98E-31 |
| ZP3 | 0.599899 | 2.595115 | 2.113007 | 2.43E-21 | 8.95E-21 |
| SRD5A3 | 5.835606 | 19.69833 | 1.755119 | 1.74E-22 | 7.14E-22 |
| DPEP2 | 6.636512 | 1.636144 | -2.02013 | 2.75E-33 | 8.06E-32 |
| SRCIN1 | 0.384226 | 1.463205 | 1.929107 | 1.14E-15 | 2.80E-15 |
| PPM1N | 0.403701 | 0.927547 | 1.200132 | 3.17E-06 | 4.50E-06 |
| MIA | 0.076117 | 1.201876 | 3.980933 | 0.015352 | 0.017492 |
| ZNF92 | 1.75342 | 4.022346 | 1.197866 | 1.88E-24 | 9.26E-24 |
| MGST1 | 20.0587 | 42.51528 | 1.083753 | 3.24E-11 | 6.06E-11 |
| DONSON | 1.526713 | 4.786749 | 1.648617 | 1.55E-32 | 3.06E-31 |
| CARD11 | 1.959326 | 7.582994 | 1.95241 | 3.89E-17 | 1.03E-16 |
| PPM1J | 0.232396 | 0.651441 | 1.487048 | 1.48E-15 | 3.60E-15 |
| DOK5 | 0.273322 | 1.481343 | 2.438233 | 3.66E-16 | 9.26E-16 |
| PPM1H | 1.984256 | 4.58986 | 1.209852 | 1.49E-09 | 2.52E-09 |
| PPM1F | 7.016774 | 3.225868 | -1.12112 | 1.39E-26 | 8.47E-26 |
| CAPN13 | 3.503563 | 11.5192 | 1.717146 | 0.007219 | 0.008417 |
| SPTSSB | 0.067526 | 1.698157 | 4.652388 | 1.24E-07 | 1.90E-07 |
| MFSD3 | 5.092371 | 11.87597 | 1.221636 | 4.08E-17 | 1.08E-16 |
| CAPN12 | 0.573502 | 2.186404 | 1.930691 | 1.50E-17 | 4.11E-17 |
| CAMP | 4.229829 | 0.764149 | -2.46867 | 2.04E-29 | 1.81E-28 |
| ZNF710 | 5.381256 | 11.73859 | 1.125244 | 1.79E-10 | 3.19E-10 |
| DOC2A | 0.25871 | 0.554079 | 1.098754 | 0.037684 | 0.041662 |
| ZNF703 | 4.605118 | 9.601545 | 1.060028 | 0.007865 | 0.009156 |
| SPP1 | 9.26432 | 288.484 | 4.960662 | 2.02E-30 | 2.18E-29 |
| MFAP4 | 243.2948 | 43.66264 | -2.47823 | 1.38E-32 | 2.79E-31 |
| DNTTIP1 | 12.14757 | 38.49636 | 1.664054 | 6.52E-26 | 3.76E-25 |
| MFAP3L | 1.876336 | 0.488571 | -1.94128 | 6.61E-29 | 5.49E-28 |
| DNMT3B | 0.373817 | 1.697957 | 2.183397 | 4.31E-23 | 1.85E-22 |
| CAMK2N2 | 0.056844 | 0.618683 | 3.44412 | 6.59E-19 | 2.01E-18 |
| ZNF692 | 2.480082 | 8.257908 | 1.735388 | 2.63E-24 | 1.28E-23 |
| MEX3A | 0.27052 | 5.454727 | 4.333699 | 1.03E-33 | 3.69E-32 |
| DNMT3A | 2.311246 | 4.786462 | 1.050289 | 3.90E-20 | 1.30E-19 |
| ZNF681 | 0.488368 | 1.189318 | 1.284096 | 1.38E-16 | 3.56E-16 |
| SPOCK2 | 100.4634 | 12.21165 | -3.04034 | 8.43E-35 | 9.78E-33 |
| PPFIA4 | 0.108938 | 0.639768 | 2.554048 | 1.07E-17 | 2.99E-17 |
| SPOCK1 | 0.295525 | 2.730587 | 3.207857 | 3.98E-13 | 8.40E-13 |
| DNM1 | 0.668171 | 1.626114 | 1.283138 | 3.59E-11 | 6.69E-11 |
| SPN | 15.06505 | 3.478662 | -2.1146 | 1.41E-32 | 2.82E-31 |
| METTL26 | 14.36081 | 28.93822 | 1.010839 | 1.78E-21 | 6.69E-21 |
| DNASE2B | 2.921888 | 0.444593 | -2.71634 | 1.31E-26 | 8.06E-26 |
| SPINK1 | 1.319622 | 146.1993 | 6.791668 | 1.77E-21 | 6.65E-21 |
| PPBP | 8.773011 | 2.348288 | -1.90146 | 1.27E-22 | 5.29E-22 |
| HILPDA | 4.523102 | 15.02985 | 1.732446 | 1.91E-15 | 4.63E-15 |
| ZNF587 | 1.437117 | 3.163394 | 1.138295 | 1.13E-19 | 3.65E-19 |
| PPAT | 0.994201 | 4.2394 | 2.092251 | 4.42E-35 | 7.13E-33 |
| DNASE1 | 0.728985 | 1.758051 | 1.270017 | 1.01E-18 | 3.05E-18 |
| SPIB | 0.678391 | 1.399431 | 1.044652 | 3.50E-05 | 4.69E-05 |
| MET | 17.25154 | 46.3165 | 1.424801 | 0.00013 | 0.000168 |
| PPARG | 12.49131 | 4.583032 | -1.44655 | 1.03E-25 | 5.74E-25 |
| MEST | 6.261665 | 16.60375 | 1.406891 | 7.08E-24 | 3.26E-23 |
| MESP2 | 0.084782 | 0.82201 | 3.277323 | 4.66E-20 | 1.55E-19 |
| DNAJC12 | 0.637536 | 13.03312 | 4.353533 | 2.16E-23 | 9.52E-23 |
| CADM1 | 23.36637 | 10.04656 | -1.21773 | 1.39E-24 | 7.01E-24 |
| POU6F2 | 0.036647 | 0.63639 | 4.118149 | 3.35E-05 | 4.49E-05 |
| MESP1 | 0.349121 | 1.968325 | 2.495171 | 5.34E-22 | 2.08E-21 |
| DNAJC10 | 3.914026 | 7.946915 | 1.021741 | 1.75E-24 | 8.66E-24 |
| DNAJB13 | 0.608471 | 1.252554 | 1.041612 | 1.72E-05 | 2.34E-05 |
| SPDYC | 0.034443 | 0.789137 | 4.517976 | 0.012279 | 0.0141 |
| MELTF | 0.447671 | 3.398201 | 2.924261 | 6.37E-24 | 2.95E-23 |
| HHIPL2 | 0.045025 | 3.151802 | 6.129292 | 1.68E-21 | 6.34E-21 |
| MELK | 0.402604 | 5.577887 | 3.792284 | 5.62E-33 | 1.32E-31 |
| HHIP | 10.12159 | 1.566188 | -2.69211 | 8.21E-31 | 9.57E-30 |
| SPDEF | 2.218434 | 22.97972 | 3.372748 | 1.94E-22 | 7.94E-22 |
| POU3F2 | 0.00794 | 1.147906 | 7.175607 | 9.14E-13 | 1.88E-12 |
| HGH1 | 5.537863 | 13.35381 | 1.26985 | 2.97E-27 | 1.94E-26 |
| DNAH14 | 0.141393 | 0.698802 | 2.305173 | 5.15E-32 | 7.91E-31 |
| CABYR | 0.1505 | 4.054008 | 4.751514 | 1.57E-27 | 1.07E-26 |
| ZNF469 | 0.359988 | 0.731389 | 1.022689 | 6.26E-07 | 9.23E-07 |
| SPC25 | 0.506184 | 2.856357 | 2.496442 | 4.21E-29 | 3.66E-28 |
| POU2AF1 | 1.171905 | 3.860912 | 1.720086 | 4.15E-12 | 8.19E-12 |
| SPC24 | 0.470754 | 3.326226 | 2.820843 | 2.53E-32 | 4.47E-31 |
| DNAAF3 | 2.311748 | 1.114241 | -1.05292 | 0.002311 | 0.002769 |
| HES6 | 1.597285 | 12.91409 | 3.015252 | 1.91E-22 | 7.83E-22 |
| DNA2 | 0.389165 | 2.046537 | 2.394733 | 7.67E-32 | 1.11E-30 |
| CABLES2 | 1.790122 | 4.822408 | 1.429696 | 1.61E-27 | 1.10E-26 |
| SPATA25 | 0.402202 | 0.830016 | 1.045219 | 1.04E-09 | 1.77E-09 |
| CA9 | 0.269007 | 13.52855 | 5.652221 | 1.57E-25 | 8.56E-25 |
| SPAG4 | 0.923316 | 7.56165 | 3.033805 | 3.77E-34 | 2.06E-32 |
| DMRTA2 | 0.021968 | 0.826842 | 5.234163 | 1.03E-11 | 1.99E-11 |
| CA4 | 18.53024 | 1.012545 | -4.19382 | 3.12E-34 | 1.91E-32 |
| POLR2H | 7.142637 | 17.57617 | 1.299092 | 3.24E-32 | 5.39E-31 |
| POLQ | 0.070479 | 0.790218 | 3.486985 | 1.25E-31 | 1.71E-30 |
| ZNF322 | 0.938846 | 2.312519 | 1.300504 | 2.02E-11 | 3.81E-11 |
| SOX9 | 3.599251 | 10.16003 | 1.497136 | 0.001224 | 0.001491 |
| DMBX1 | 0.006498 | 0.649561 | 6.643222 | 1.24E-28 | 9.84E-28 |
| C8orf76 | 1.83722 | 4.43489 | 1.271374 | 1.79E-27 | 1.20E-26 |
| SOX7 | 10.20786 | 1.494049 | -2.77238 | 5.77E-32 | 8.72E-31 |
| POLE2 | 0.419142 | 2.394512 | 2.514219 | 1.83E-32 | 3.49E-31 |
| HELLS | 0.299349 | 1.786048 | 2.576873 | 3.95E-32 | 6.33E-31 |
| MDK | 16.85298 | 152.947 | 3.181956 | 4.49E-30 | 4.49E-29 |
| HECW2 | 4.352237 | 1.94634 | -1.16099 | 5.03E-16 | 1.26E-15 |
| DLX5 | 0.116954 | 0.855583 | 2.870965 | 1.08E-12 | 2.21E-12 |
| C6orf52 | 0.692502 | 1.430859 | 1.046991 | 1.67E-07 | 2.53E-07 |
| DLX4 | 0.414788 | 1.050372 | 1.340452 | 3.37E-08 | 5.32E-08 |
| SOX2 | 2.928975 | 9.544951 | 1.704342 | 0.000497 | 0.000623 |
| SOX18 | 10.59604 | 3.659028 | -1.53399 | 8.49E-19 | 2.57E-18 |
| C5orf34 | 0.329216 | 1.491569 | 2.179725 | 1.82E-29 | 1.63E-28 |
| ZNF239 | 0.758345 | 2.69116 | 1.827302 | 1.93E-28 | 1.48E-27 |
| SOX17 | 6.132305 | 1.059538 | -2.53299 | 1.29E-30 | 1.47E-29 |
| MCM8 | 0.860975 | 2.471919 | 1.521588 | 2.60E-26 | 1.55E-25 |
| SOX12 | 2.788093 | 7.712259 | 1.467875 | 1.70E-21 | 6.41E-21 |
| PODXL2 | 2.449605 | 25.5601 | 3.383272 | 2.04E-22 | 8.29E-22 |
| MCM7 | 11.44687 | 30.22744 | 1.400906 | 6.15E-25 | 3.20E-24 |
| HCN3 | 0.48822 | 1.850806 | 1.922551 | 4.34E-26 | 2.53E-25 |
| DLGAP5 | 0.345981 | 5.004003 | 3.854316 | 1.42E-33 | 4.69E-32 |
| C5AR1 | 32.14265 | 9.266557 | -1.79438 | 3.00E-30 | 3.10E-29 |
| SOX11 | 0.020063 | 0.662692 | 5.045762 | 1.40E-11 | 2.69E-11 |
| MCM6 | 4.696438 | 14.41752 | 1.618184 | 2.27E-27 | 1.51E-26 |
| ZNF217 | 5.850371 | 16.54703 | 1.499972 | 1.94E-29 | 1.72E-28 |
| SOSTDC1 | 14.24233 | 1.189914 | -3.58126 | 1.71E-30 | 1.89E-29 |
| MCM4 | 2.791688 | 14.32319 | 2.359143 | 1.51E-33 | 4.90E-32 |
| HCK | 27.3316 | 12.18712 | -1.16521 | 1.95E-23 | 8.61E-23 |
| DLC1 | 33.07002 | 7.671232 | -2.10799 | 1.36E-30 | 1.52E-29 |
| POC1A | 1.395251 | 4.564433 | 1.709911 | 7.64E-28 | 5.42E-27 |
| MCM3 | 14.21239 | 29.34394 | 1.045913 | 1.54E-24 | 7.72E-24 |
| HCAR1 | 0.396951 | 2.357964 | 2.57051 | 8.11E-11 | 1.48E-10 |
| C2CD4D | 0.192345 | 1.201663 | 2.643263 | 3.48E-27 | 2.24E-26 |
| MCM2 | 2.730414 | 12.28471 | 2.169672 | 2.43E-30 | 2.55E-29 |
| HBEGF | 49.23505 | 9.127159 | -2.43145 | 1.38E-29 | 1.26E-28 |
| ZNF117 | 2.175158 | 6.375972 | 1.551525 | 1.82E-10 | 3.26E-10 |
| PNPLA6 | 26.29036 | 9.907712 | -1.40791 | 2.67E-32 | 4.65E-31 |
| MCM10 | 0.142682 | 1.870008 | 3.712168 | 2.41E-32 | 4.31E-31 |
| DIRAS1 | 0.303557 | 1.984963 | 2.709072 | 5.21E-13 | 1.09E-12 |
| PNOC | 0.284506 | 0.899527 | 1.660708 | 5.23E-12 | 1.03E-11 |
| HAVCR1 | 0.033876 | 1.381952 | 5.350293 | 3.40E-18 | 9.82E-18 |
| DIPK2B | 6.224066 | 2.006649 | -1.63307 | 4.86E-23 | 2.07E-22 |
| PNMA3 | 0.102681 | 0.736268 | 2.842068 | 2.88E-07 | 4.32E-07 |
| MC1R | 0.453179 | 1.118391 | 1.30327 | 1.95E-15 | 4.71E-15 |
| C1QTNF6 | 0.900043 | 4.700747 | 2.384824 | 4.70E-30 | 4.67E-29 |
| C1QTNF3 | 1.369691 | 3.108929 | 1.182567 | 0.002038 | 0.002449 |
| ZIC2 | 0.014699 | 0.615569 | 5.388143 | 5.70E-12 | 1.12E-11 |
| SNX22 | 3.707964 | 1.122942 | -1.72334 | 1.27E-28 | 1.00E-27 |
| HASPIN | 0.254867 | 1.086212 | 2.09149 | 3.50E-23 | 1.52E-22 |
| DHX34 | 2.338032 | 5.011589 | 1.099974 | 5.34E-23 | 2.27E-22 |
| C1QTNF12 | 0.213541 | 0.86327 | 2.015299 | 2.48E-10 | 4.37E-10 |
| HAPLN3 | 2.292753 | 5.749931 | 1.326464 | 3.83E-14 | 8.56E-14 |
| ZGRF1 | 0.261392 | 0.638358 | 1.28815 | 2.91E-16 | 7.39E-16 |
| HAL | 0.342345 | 2.252861 | 2.718234 | 8.95E-08 | 1.38E-07 |
| DHRS11 | 1.007451 | 2.308387 | 1.196175 | 7.27E-25 | 3.76E-24 |
| MB | 0.496206 | 4.88369 | 3.298961 | 3.40E-21 | 1.24E-20 |
| HAGHL | 0.564506 | 1.499691 | 1.409604 | 9.69E-14 | 2.12E-13 |
| DHFR | 1.421009 | 2.971057 | 1.064061 | 4.49E-22 | 1.77E-21 |
| C1QB | 427.7749 | 158.0551 | -1.43642 | 9.56E-24 | 4.37E-23 |
| SNRPE | 15.22746 | 36.356 | 1.255519 | 3.00E-30 | 3.10E-29 |
| PMEL | 0.514618 | 1.10553 | 1.103166 | 1.57E-19 | 5.03E-19 |
| C1QA | 436.3972 | 163.6435 | -1.41509 | 8.49E-26 | 4.80E-25 |
| ZFP69B | 0.311472 | 1.134739 | 1.865186 | 9.62E-31 | 1.11E-29 |
| SNRPA1 | 4.116106 | 8.782022 | 1.093273 | 2.23E-30 | 2.37E-29 |
| MARCO | 216.7747 | 29.67918 | -2.86867 | 3.67E-33 | 9.87E-32 |
| DGKA | 1.561663 | 3.405533 | 1.124797 | 1.39E-17 | 3.83E-17 |
| C1orf162 | 24.12059 | 7.694499 | -1.64837 | 2.31E-31 | 3.04E-30 |
| ZEB2 | 5.386688 | 2.468137 | -1.12598 | 3.08E-27 | 2.00E-26 |
| PLXNA3 | 2.946945 | 6.62178 | 1.167999 | 2.79E-16 | 7.10E-16 |
| MARCKSL1 | 31.3964 | 129.6716 | 2.046191 | 7.56E-29 | 6.25E-28 |
| C1orf159 | 1.060747 | 2.22626 | 1.069541 | 1.28E-20 | 4.43E-20 |
| ZEB1 | 6.721329 | 2.969283 | -1.17863 | 1.77E-23 | 7.90E-23 |
| SNRNP25 | 4.903559 | 10.12453 | 1.045954 | 1.74E-23 | 7.80E-23 |
| DGAT2 | 1.007249 | 2.050411 | 1.025494 | 2.91E-08 | 4.62E-08 |
| C1orf112 | 0.468137 | 1.748614 | 1.901208 | 2.86E-34 | 1.91E-32 |
| ZDHHC9 | 15.41567 | 40.92771 | 1.408681 | 5.30E-23 | 2.25E-22 |
| DEPP1 | 124.1783 | 40.68355 | -1.6099 | 3.63E-10 | 6.35E-10 |
| MAPK8IP2 | 0.613394 | 1.835488 | 1.581279 | 0.000204 | 0.000263 |
| DEPDC1B | 0.165097 | 2.246302 | 3.766168 | 9.82E-34 | 3.56E-32 |
| PLPP5 | 6.103883 | 14.7333 | 1.271281 | 3.64E-25 | 1.93E-24 |
| DEPDC1 | 0.128865 | 2.080523 | 4.013012 | 4.16E-33 | 1.08E-31 |
| SNCA | 1.880944 | 0.741169 | -1.34358 | 3.54E-26 | 2.09E-25 |
| PLPP2 | 2.361901 | 13.37117 | 2.501106 | 1.17E-29 | 1.07E-28 |
| MAP7D2 | 0.237706 | 2.630744 | 3.468219 | 1.18E-18 | 3.53E-18 |
| PLLP | 9.812116 | 3.754716 | -1.38586 | 4.19E-26 | 2.45E-25 |
| ZBTB41 | 2.645954 | 5.523921 | 1.061905 | 2.04E-23 | 9.02E-23 |
| SMPDL3B | 6.762494 | 26.37782 | 1.963698 | 2.19E-22 | 8.86E-22 |
| PLK4 | 0.478726 | 2.207302 | 2.205012 | 4.96E-29 | 4.25E-28 |
| PLK1 | 0.577681 | 5.911287 | 3.355128 | 2.65E-34 | 1.91E-32 |
| C12orf45 | 3.461728 | 7.454181 | 1.106557 | 6.24E-28 | 4.53E-27 |
| GTSE1 | 0.291776 | 2.800319 | 3.262656 | 1.21E-32 | 2.49E-31 |
| C11orf80 | 2.255927 | 6.700564 | 1.570562 | 2.66E-31 | 3.44E-30 |
| MAOB | 18.54889 | 7.109124 | -1.38359 | 8.93E-26 | 5.03E-25 |
| ZBP1 | 0.46696 | 1.218957 | 1.384278 | 1.02E-09 | 1.74E-09 |
| PLEKHO2 | 27.51541 | 13.66514 | -1.00974 | 1.83E-24 | 9.07E-24 |
| MANEAL | 1.745763 | 6.861815 | 1.974733 | 3.50E-21 | 1.28E-20 |
| PLEKHH2 | 5.392449 | 2.534392 | -1.0893 | 1.40E-16 | 3.61E-16 |
| PLEKHG4 | 1.38195 | 3.456545 | 1.322625 | 3.58E-09 | 5.91E-09 |
| YJEFN3 | 0.494851 | 1.054162 | 1.091029 | 4.91E-05 | 6.54E-05 |
| BZW2 | 10.23896 | 28.11381 | 1.45721 | 2.73E-32 | 4.65E-31 |
| MAL | 9.219382 | 4.106129 | -1.16689 | 5.33E-20 | 1.76E-19 |
| BUB1B | 0.31596 | 3.725579 | 3.559653 | 1.75E-34 | 1.54E-32 |
| DDX39A | 7.57036 | 15.90453 | 1.071003 | 8.68E-24 | 3.98E-23 |
| BUB1 | 0.62834 | 4.43815 | 2.82034 | 7.17E-30 | 6.82E-29 |
| SMC1B | 0.047218 | 0.613508 | 3.69967 | 5.86E-12 | 1.14E-11 |
| YBX2 | 0.047643 | 1.389752 | 4.866416 | 1.32E-20 | 4.58E-20 |
| DDX11 | 1.108671 | 3.377629 | 1.60718 | 5.13E-22 | 2.01E-21 |
| PLEK2 | 1.780108 | 12.68838 | 2.833471 | 9.80E-33 | 2.11E-31 |
| PLD5 | 0.087228 | 0.660598 | 2.920912 | 1.09E-05 | 1.50E-05 |
| MAGED1 | 22.05251 | 46.95869 | 1.090449 | 5.24E-24 | 2.46E-23 |
| DCUN1D5 | 7.727219 | 15.4667 | 1.001144 | 1.52E-25 | 8.30E-25 |
| DCSTAMP | 2.932208 | 0.717602 | -2.03073 | 2.42E-20 | 8.20E-20 |
| BST1 | 4.888719 | 2.424576 | -1.01172 | 5.20E-24 | 2.44E-23 |
| XRCC2 | 0.149462 | 1.570328 | 3.393216 | 2.62E-34 | 1.91E-32 |
| SLFN13 | 1.678373 | 5.500784 | 1.712574 | 6.23E-22 | 2.42E-21 |
| PLCH2 | 0.282305 | 0.726418 | 1.363545 | 0.000269 | 0.000344 |
| MAGEC1 | 0.001756 | 0.648726 | 8.528803 | 5.80E-11 | 1.06E-10 |
| GRIN2D | 0.248595 | 1.605198 | 2.69088 | 2.91E-12 | 5.78E-12 |
| DCST2 | 0.155836 | 0.860862 | 2.465756 | 9.40E-23 | 3.93E-22 |
| XPR1 | 4.979491 | 20.57072 | 2.046522 | 2.00E-30 | 2.17E-29 |
| SLF1 | 0.847602 | 1.843395 | 1.120906 | 2.74E-13 | 5.83E-13 |
| XPOT | 7.55555 | 15.92054 | 1.075281 | 2.75E-24 | 1.33E-23 |
| MAGEA3 | 0.018058 | 5.829635 | 8.334592 | 1.65E-07 | 2.50E-07 |
| DCN | 80.76485 | 32.3461 | -1.32014 | 4.60E-22 | 1.81E-21 |
| SLCO4C1 | 7.720221 | 3.735107 | -1.04749 | 1.54E-20 | 5.31E-20 |
| XKRX | 0.65563 | 5.132237 | 2.968633 | 2.88E-17 | 7.75E-17 |
| SLCO2B1 | 23.48197 | 9.735124 | -1.27028 | 2.37E-25 | 1.27E-24 |
| MAGEA12 | 0.015506 | 2.89764 | 7.545872 | 1.49E-05 | 2.04E-05 |
| GREM1 | 0.131946 | 4.714221 | 5.158999 | 2.67E-28 | 2.00E-27 |
| BRIX1 | 3.676698 | 9.514308 | 1.371688 | 6.95E-31 | 8.16E-30 |
| SLCO2A1 | 41.73055 | 13.37395 | -1.64168 | 4.32E-22 | 1.70E-21 |
| PLAU | 10.13356 | 67.75672 | 2.741223 | 1.24E-20 | 4.30E-20 |
| GRB7 | 6.127748 | 15.10178 | 1.30129 | 9.22E-18 | 2.60E-17 |
| BRIP1 | 0.150043 | 1.099309 | 2.873148 | 2.48E-32 | 4.41E-31 |
| MAGEA10 | 0.005566 | 1.502189 | 8.076261 | 3.52E-08 | 5.54E-08 |
| SLC9A5 | 0.47994 | 1.029011 | 1.100333 | 1.65E-07 | 2.51E-07 |
| PLAC9 | 19.52898 | 3.360133 | -2.53903 | 1.45E-34 | 1.34E-32 |
| MAD2L2 | 4.541793 | 9.410412 | 1.050996 | 6.41E-20 | 2.11E-19 |
| GPX2 | 1.192359 | 77.04338 | 6.013781 | 1.91E-08 | 3.04E-08 |
| DCAF13 | 2.357971 | 6.622089 | 1.48974 | 4.16E-33 | 1.08E-31 |
| BRDT | 0.020958 | 1.702053 | 6.343623 | 6.97E-10 | 1.20E-09 |
| SLC7A7 | 18.21189 | 7.437519 | -1.29199 | 2.53E-28 | 1.91E-27 |
| MAD2L1 | 0.576884 | 3.733171 | 2.694049 | 2.56E-31 | 3.33E-30 |
| GPT2 | 1.074532 | 12.18198 | 3.502969 | 6.08E-35 | 8.18E-33 |
| BRCA2 | 0.339542 | 0.843169 | 1.312233 | 3.66E-16 | 9.26E-16 |
| PLA2G4F | 15.00374 | 2.525668 | -2.57059 | 1.92E-32 | 3.61E-31 |
| MACC1 | 3.93194 | 8.73907 | 1.152239 | 2.42E-05 | 3.27E-05 |
| BRCA1 | 0.689916 | 2.127065 | 1.624371 | 9.06E-20 | 2.94E-19 |
| SLC7A5 | 4.325764 | 30.81443 | 2.83258 | 2.35E-27 | 1.56E-26 |
| GPRIN1 | 0.373279 | 2.709483 | 2.85969 | 4.57E-31 | 5.69E-30 |
| DBNDD1 | 1.699044 | 7.289974 | 2.101191 | 8.05E-29 | 6.56E-28 |
| WNT7A | 3.087012 | 0.89429 | -1.7874 | 6.37E-28 | 4.61E-27 |
| SLC7A11 | 0.540253 | 4.988254 | 3.206827 | 1.29E-18 | 3.86E-18 |
| PLA2G4A | 3.678173 | 21.35452 | 2.53748 | 8.18E-13 | 1.69E-12 |
| DBF4 | 1.138061 | 3.383468 | 1.571925 | 1.29E-28 | 1.02E-27 |
| WNT5B | 1.222304 | 2.495168 | 1.029533 | 0.000274 | 0.00035 |
| SLC7A10 | 0.036954 | 1.126402 | 4.929857 | 4.63E-12 | 9.13E-12 |
| PLA2G2D | 1.737763 | 4.215494 | 1.27847 | 2.96E-05 | 3.98E-05 |
| GPR89B | 0.330867 | 0.737493 | 1.156379 | 1.59E-25 | 8.61E-25 |
| BPIFA2 | 0.109904 | 4.721623 | 5.424962 | 3.04E-12 | 6.04E-12 |
| SLC6A4 | 29.26943 | 0.461356 | -5.98737 | 2.75E-34 | 1.91E-32 |
| LYVE1 | 18.66214 | 2.270797 | -3.03884 | 1.79E-32 | 3.44E-31 |
| SLC6A3 | 0.134945 | 2.324019 | 4.106184 | 0.001686 | 0.002037 |
| SLC52A2 | 10.13001 | 29.99057 | 1.565874 | 6.08E-30 | 5.86E-29 |
| PKMYT1 | 0.286727 | 2.541627 | 3.148002 | 3.55E-31 | 4.50E-30 |
| LYPD6B | 0.455289 | 1.989392 | 2.127474 | 0.000638 | 0.000794 |
| BORA | 0.782256 | 1.891964 | 1.274172 | 8.54E-23 | 3.58E-22 |
| PKM | 78.9756 | 161.822 | 1.034929 | 9.55E-26 | 5.36E-25 |
| GPR4 | 6.089397 | 2.251548 | -1.43538 | 2.43E-21 | 8.95E-21 |
| DAPK2 | 5.475327 | 1.577199 | -1.79558 | 4.53E-30 | 4.52E-29 |
| BOP1 | 4.983937 | 17.78826 | 1.835568 | 5.60E-30 | 5.47E-29 |
| LYG1 | 0.352831 | 0.929788 | 1.397924 | 1.03E-12 | 2.11E-12 |
| DAP3 | 13.30665 | 27.13381 | 1.027945 | 1.44E-32 | 2.86E-31 |
| BMX | 1.612946 | 0.778034 | -1.05179 | 3.80E-27 | 2.44E-26 |
| SLC44A5 | 0.380079 | 2.391342 | 2.65345 | 7.33E-11 | 1.34E-10 |
| LY86 | 19.44194 | 8.213823 | -1.24305 | 2.48E-23 | 1.09E-22 |
| DACH1 | 3.868593 | 0.785387 | -2.30033 | 2.17E-30 | 2.32E-29 |
| WDR86 | 0.463497 | 2.375686 | 2.357713 | 1.38E-13 | 2.98E-13 |
| SLC39A14 | 4.323341 | 8.734559 | 1.014588 | 1.97E-11 | 3.73E-11 |
| GPR18 | 0.427108 | 0.984455 | 1.204726 | 0.000496 | 0.000622 |
| WDR76 | 1.171566 | 2.870057 | 1.292641 | 2.55E-20 | 8.62E-20 |
| GPR174 | 0.555535 | 1.141781 | 1.039336 | 0.007773 | 0.009052 |
| PIP5KL1 | 0.945041 | 3.014324 | 1.673386 | 7.30E-15 | 1.71E-14 |
| BLM | 0.311872 | 1.479816 | 2.246392 | 1.19E-31 | 1.65E-30 |
| PIMREG | 0.251039 | 2.543709 | 3.34095 | 6.69E-31 | 7.90E-30 |
| WDHD1 | 0.727597 | 2.808125 | 1.948396 | 2.74E-28 | 2.05E-27 |
| SLC31A2 | 2.494554 | 0.989293 | -1.33431 | 2.47E-26 | 1.48E-25 |
| GPR146 | 2.034673 | 0.468839 | -2.11763 | 1.74E-31 | 2.36E-30 |
| PIM2 | 13.11401 | 28.94863 | 1.142387 | 1.97E-10 | 3.51E-10 |
| LTA | 0.360051 | 0.734822 | 1.029192 | 6.42E-10 | 1.11E-09 |
| GPR143 | 0.763882 | 1.925767 | 1.334011 | 3.53E-07 | 5.27E-07 |
| BIRC5 | 0.806704 | 11.4556 | 3.82787 | 2.17E-32 | 3.96E-31 |
| WASF1 | 1.088084 | 2.89759 | 1.413063 | 8.89E-16 | 2.20E-15 |
| PILRB | 0.57161 | 1.389185 | 1.281136 | 1.81E-08 | 2.88E-08 |
| LST1 | 18.83928 | 8.171183 | -1.20513 | 3.56E-22 | 1.42E-21 |
| PILRA | 15.00874 | 5.86919 | -1.35457 | 1.69E-27 | 1.14E-26 |
| CYP27C1 | 0.104288 | 0.899661 | 3.10881 | 2.06E-16 | 5.29E-16 |
| VWF | 93.31578 | 20.74349 | -2.16946 | 2.06E-31 | 2.77E-30 |
| CYP27B1 | 0.431851 | 2.429642 | 2.492137 | 5.47E-29 | 4.67E-28 |
| SLC2A1 | 3.128873 | 38.73479 | 3.629915 | 3.56E-32 | 5.77E-31 |
| CYP27A1 | 69.03513 | 23.32754 | -1.5653 | 3.54E-28 | 2.63E-27 |
| BIK | 1.975254 | 8.529409 | 2.110408 | 5.42E-22 | 2.11E-21 |
| SLC29A4 | 0.566021 | 5.402046 | 3.254578 | 1.05E-20 | 3.68E-20 |
| GPM6B | 6.035741 | 1.72885 | -1.80372 | 1.13E-29 | 1.04E-28 |
| GPI | 18.00716 | 42.69694 | 1.245562 | 1.37E-28 | 1.07E-27 |
| VSTM2L | 9.18506 | 41.43832 | 2.173604 | 2.64E-10 | 4.64E-10 |
| GPC3 | 50.44584 | 12.76886 | -1.98211 | 1.92E-29 | 1.71E-28 |
| BEX5 | 7.131386 | 3.269441 | -1.12514 | 2.57E-15 | 6.18E-15 |
| VSIR | 27.87903 | 10.20886 | -1.44936 | 1.29E-33 | 4.35E-32 |
| LRRN4CL | 1.062929 | 0.510911 | -1.0569 | 1.36E-12 | 2.76E-12 |
| GPC2 | 0.071085 | 0.828793 | 3.543392 | 2.49E-26 | 1.49E-25 |
| CYBRD1 | 85.45423 | 27.54254 | -1.63349 | 6.14E-30 | 5.89E-29 |
| VSIG4 | 113.0705 | 24.14763 | -2.22727 | 6.45E-31 | 7.67E-30 |
| LRRN3 | 3.891022 | 0.693411 | -2.48837 | 1.04E-29 | 9.76E-29 |
| CYBB | 63.4362 | 28.48505 | -1.1551 | 1.09E-17 | 3.04E-17 |
| SLC26A6 | 1.24139 | 3.516174 | 1.50205 | 5.50E-24 | 2.57E-23 |
| PIF1 | 0.172689 | 0.980296 | 2.505039 | 6.98E-26 | 4.00E-25 |
| VPREB3 | 1.445418 | 3.196846 | 1.145162 | 3.71E-17 | 9.89E-17 |
| BCL6B | 9.608798 | 3.11141 | -1.62679 | 2.31E-28 | 1.75E-27 |
| PIAS3 | 5.898889 | 13.49011 | 1.193387 | 4.21E-27 | 2.68E-26 |
| BCL2L12 | 3.696463 | 7.992405 | 1.112484 | 4.75E-24 | 2.25E-23 |
| GOLM1 | 11.25503 | 75.79096 | 2.751456 | 7.41E-35 | 9.35E-33 |
| CXCR2 | 3.060057 | 0.567605 | -2.4306 | 4.92E-28 | 3.60E-27 |
| BCL2L10 | 0.032185 | 0.62626 | 4.28229 | 2.17E-22 | 8.81E-22 |
| LRRC4 | 5.318492 | 2.56274 | -1.05333 | 1.48E-14 | 3.39E-14 |
| CXCR1 | 2.556919 | 0.390156 | -2.71228 | 1.80E-23 | 8.00E-23 |
| LRRC26 | 0.128208 | 0.744699 | 2.538171 | 0.010174 | 0.011764 |
| CXCL9 | 14.67827 | 40.45107 | 1.462496 | 0.000543 | 0.000678 |
| BCL2A1 | 20.93467 | 9.994504 | -1.06669 | 5.47E-16 | 1.37E-15 |
| GOLGA7B | 0.621649 | 4.481692 | 2.849871 | 3.88E-22 | 1.54E-21 |
| PHKA1 | 1.252076 | 4.28556 | 1.775162 | 3.17E-32 | 5.32E-31 |
| CXCL3 | 9.594715 | 3.182348 | -1.59215 | 7.89E-11 | 1.44E-10 |
| VIM | 320.7725 | 155.0503 | -1.04881 | 7.64E-28 | 5.42E-27 |
| LRP8 | 0.78251 | 2.101451 | 1.425204 | 4.07E-15 | 9.71E-15 |
| CXCL14 | 4.0955 | 87.22683 | 4.412661 | 9.19E-13 | 1.89E-12 |
| SLC22A4 | 3.295204 | 1.539406 | -1.09799 | 1.79E-07 | 2.71E-07 |
| PHEX | 1.101074 | 0.518669 | -1.08603 | 1.53E-14 | 3.52E-14 |
| GNG11 | 38.29522 | 12.7475 | -1.58695 | 7.39E-25 | 3.81E-24 |
| CXCL13 | 8.026652 | 26.22198 | 1.707906 | 1.14E-18 | 3.41E-18 |
| VGLL3 | 4.948747 | 2.393836 | -1.04774 | 1.03E-21 | 3.94E-21 |
| CXCL12 | 14.45391 | 6.667421 | -1.11626 | 1.11E-20 | 3.86E-20 |
| VEPH1 | 13.57385 | 2.42967 | -2.482 | 4.63E-33 | 1.16E-31 |
| CXCL10 | 16.62578 | 35.43376 | 1.091703 | 0.001579 | 0.001912 |
| SLC17A9 | 0.870874 | 5.421928 | 2.638269 | 3.36E-23 | 1.46E-22 |
| GMNN | 2.884415 | 8.100307 | 1.489698 | 1.63E-27 | 1.10E-26 |
| BASP1 | 11.89493 | 43.69997 | 1.877286 | 8.88E-13 | 1.83E-12 |
| PGGHG | 7.665204 | 31.8949 | 2.05693 | 2.97E-10 | 5.22E-10 |
| GMDS | 2.596787 | 7.904308 | 1.605911 | 2.40E-30 | 2.54E-29 |
| CUZD1 | 0.214295 | 0.768713 | 1.842845 | 7.41E-11 | 1.35E-10 |
| BARX2 | 0.216223 | 3.4182 | 3.982642 | 7.23E-22 | 2.79E-21 |
| SLC16A8 | 0.151627 | 0.76686 | 2.338433 | 7.83E-15 | 1.83E-14 |
| PGF | 0.793966 | 2.314486 | 1.543544 | 1.31E-15 | 3.22E-15 |
| BARX1 | 0.047652 | 9.423461 | 7.627587 | 1.07E-17 | 2.99E-17 |
| VCAN | 5.140556 | 19.55508 | 1.927547 | 1.37E-14 | 3.16E-14 |
| SLC16A7 | 0.838938 | 2.102488 | 1.325461 | 6.13E-06 | 8.56E-06 |
| LRFN4 | 2.46813 | 6.204651 | 1.329932 | 1.58E-10 | 2.84E-10 |
| GLYATL2 | 0.041974 | 1.192861 | 4.828795 | 0.001284 | 0.001561 |
| SLC16A6 | 2.425501 | 1.16557 | -1.05725 | 5.31E-14 | 1.18E-13 |
| SLC16A3 | 8.072909 | 22.64138 | 1.487801 | 3.28E-17 | 8.77E-17 |
| PFN2 | 10.20355 | 30.09917 | 1.560653 | 6.59E-10 | 1.13E-09 |
| SLC16A14 | 0.999568 | 7.943011 | 2.99031 | 1.40E-12 | 2.84E-12 |
| PFKP | 7.629697 | 36.91109 | 2.274357 | 1.22E-26 | 7.50E-26 |
| SLC16A11 | 3.669493 | 1.416164 | -1.37359 | 2.02E-25 | 1.09E-24 |
| LPGAT1 | 5.677511 | 13.53748 | 1.253629 | 2.52E-27 | 1.67E-26 |
| CTSV | 0.403291 | 2.110804 | 2.387899 | 5.12E-13 | 1.07E-12 |
| BACE2 | 4.145421 | 12.48236 | 1.5903 | 3.15E-11 | 5.90E-11 |
| CTSK | 20.54751 | 42.6957 | 1.055128 | 9.40E-08 | 1.44E-07 |
| SLC15A3 | 18.62506 | 8.098175 | -1.20158 | 6.58E-25 | 3.41E-24 |
| PFDN2 | 38.79847 | 80.88077 | 1.059797 | 8.44E-25 | 4.32E-24 |
| LPAR2 | 3.21481 | 6.86847 | 1.095255 | 3.39E-23 | 1.47E-22 |
| CTSG | 3.488351 | 1.069993 | -1.70494 | 6.33E-14 | 1.40E-13 |
| VAMP2 | 37.34693 | 18.40307 | -1.02104 | 1.66E-29 | 1.49E-28 |
| SLC15A2 | 12.24691 | 4.522993 | -1.43707 | 3.96E-27 | 2.53E-26 |
| PF4 | 2.190984 | 0.588709 | -1.89595 | 5.50E-20 | 1.82E-19 |
| SLC12A8 | 1.756905 | 4.796234 | 1.448866 | 3.64E-22 | 1.45E-21 |
| B4GALT3 | 8.739199 | 18.94951 | 1.116588 | 1.27E-30 | 1.45E-29 |
| GLDN | 4.628102 | 0.909076 | -2.34795 | 2.66E-29 | 2.34E-28 |
| B4GALNT1 | 0.126591 | 0.793924 | 2.648822 | 1.06E-11 | 2.05E-11 |
| GLDC | 0.180111 | 1.448384 | 3.007482 | 3.07E-09 | 5.09E-09 |
| B3GNT4 | 0.125486 | 0.587636 | 2.227396 | 8.54E-18 | 2.41E-17 |
| GLB1L3 | 0.068098 | 2.755999 | 5.338819 | 4.41E-09 | 7.24E-09 |
| B3GAT1 | 0.113759 | 0.616411 | 2.437917 | 0.006293 | 0.007361 |
| PECAM1 | 111.7933 | 24.93141 | -2.1648 | 7.32E-36 | 4.47E-33 |
| LMNB2 | 6.641813 | 15.18364 | 1.192869 | 1.43E-19 | 4.57E-19 |
| B3GALNT1 | 8.292141 | 3.766131 | -1.13866 | 2.32E-21 | 8.59E-21 |
| SLC11A1 | 12.84329 | 4.077155 | -1.65538 | 9.64E-26 | 5.40E-25 |
| PEAR1 | 4.838707 | 1.325121 | -1.8685 | 2.27E-30 | 2.41E-29 |
| LMNB1 | 3.371487 | 13.89547 | 2.043158 | 1.61E-29 | 1.45E-28 |
| CTLA4 | 0.97538 | 2.222658 | 1.188249 | 2.14E-09 | 3.58E-09 |
| PDZD11 | 14.82463 | 30.26292 | 1.029555 | 3.85E-26 | 2.27E-25 |
| SLAMF9 | 0.250731 | 1.034905 | 2.045285 | 9.79E-12 | 1.90E-11 |
| PDX1 | 0.001 | 0.594199 | 9.214989 | 7.12E-13 | 1.47E-12 |
| AURKB | 0.562162 | 6.77006 | 3.590111 | 8.17E-33 | 1.84E-31 |
| SKP2 | 2.257473 | 5.941725 | 1.396173 | 2.45E-19 | 7.71E-19 |
| LIPA | 71.36661 | 33.63382 | -1.08534 | 3.58E-14 | 8.01E-14 |
| GINS4 | 0.24228 | 1.44795 | 2.579268 | 7.45E-32 | 1.09E-30 |
| AURKA | 1.280169 | 8.756508 | 2.774022 | 2.56E-31 | 3.33E-30 |
| PDLIM4 | 2.838773 | 7.214417 | 1.345615 | 4.92E-07 | 7.29E-07 |
| GINS2 | 0.499615 | 3.990502 | 2.997682 | 2.19E-32 | 3.97E-31 |
| AUNIP | 0.223502 | 1.246178 | 2.479153 | 1.49E-27 | 1.02E-26 |
| LINGO1 | 0.198593 | 1.217602 | 2.616156 | 6.07E-18 | 1.73E-17 |
| GINS1 | 0.453985 | 4.18352 | 3.204 | 9.31E-35 | 1.00E-32 |
| CST1 | 0.288081 | 26.29625 | 6.512238 | 5.53E-28 | 4.03E-27 |
| PDK1 | 0.666273 | 2.481857 | 1.897234 | 1.33E-30 | 1.50E-29 |
| GIMAP5 | 1.295716 | 0.486417 | -1.41348 | 4.89E-23 | 2.09E-22 |
| UQCC3 | 4.250509 | 9.65516 | 1.183664 | 3.82E-23 | 1.65E-22 |
| SKA3 | 0.246597 | 2.522875 | 3.354841 | 3.64E-33 | 9.87E-32 |
| PDIA4 | 41.76729 | 156.9159 | 1.909546 | 9.27E-34 | 3.45E-32 |
| LIN7A | 3.568346 | 0.718247 | -2.3127 | 4.29E-33 | 1.10E-31 |
| UPK3A | 0.169615 | 1.638396 | 3.271948 | 1.80E-11 | 3.42E-11 |
| SKA1 | 0.23769 | 2.169117 | 3.189953 | 1.33E-30 | 1.50E-29 |
| PDGFRL | 2.442655 | 7.366386 | 1.592507 | 6.34E-17 | 1.67E-16 |
| UPK2 | 0.275323 | 0.958351 | 1.799428 | 0.003025 | 0.003603 |
| SIX4 | 0.74516 | 3.067811 | 2.041587 | 1.65E-24 | 8.19E-24 |
| GGH | 3.334023 | 9.800934 | 1.555655 | 7.02E-08 | 1.09E-07 |
| SIX1 | 1.03436 | 5.704801 | 2.463438 | 1.91E-18 | 5.65E-18 |
| GGCT | 10.0669 | 35.69088 | 1.825936 | 3.22E-34 | 1.91E-32 |
| PDF | 0.679124 | 1.591907 | 1.22901 | 3.95E-23 | 1.70E-22 |
| LIMK1 | 6.225173 | 14.96892 | 1.265784 | 3.47E-22 | 1.39E-21 |
| GFRA3 | 1.508644 | 12.73034 | 3.076947 | 0.000699 | 0.000867 |
| ATP6V1C2 | 0.414814 | 3.571463 | 3.10598 | 1.91E-25 | 1.03E-24 |
| SIRPG | 0.616733 | 1.849157 | 1.584149 | 2.80E-14 | 6.31E-14 |
| LIME1 | 0.430336 | 1.191115 | 1.468776 | 4.53E-14 | 1.01E-13 |
| GFRA1 | 1.59482 | 0.635216 | -1.32808 | 2.49E-19 | 7.82E-19 |
| SIRPB1 | 5.629878 | 0.87858 | -2.67986 | 4.68E-33 | 1.16E-31 |
| UMODL1 | 0.051288 | 1.065216 | 4.376378 | 1.91E-13 | 4.11E-13 |
| SIRPA | 25.99414 | 12.09253 | -1.10407 | 2.88E-24 | 1.39E-23 |
| PDE2A | 2.794141 | 0.907032 | -1.62318 | 1.24E-25 | 6.86E-25 |
| GFOD1 | 2.74506 | 0.891225 | -1.62298 | 3.05E-27 | 1.99E-26 |
| ATP6V0D2 | 3.037014 | 1.221747 | -1.31371 | 0.014192 | 0.016222 |
| PDCD5 | 10.82482 | 22.17287 | 1.034453 | 1.13E-25 | 6.26E-25 |
| LILRA6 | 2.432396 | 1.037135 | -1.22977 | 6.84E-21 | 2.42E-20 |
| UHRF1 | 0.292433 | 3.329679 | 3.509203 | 7.19E-34 | 3.11E-32 |
| SIGLEC6 | 1.103944 | 0.526184 | -1.06903 | 3.61E-17 | 9.64E-17 |
| PDCD2L | 2.096831 | 6.389493 | 1.607491 | 4.79E-31 | 5.88E-30 |
| LILRA5 | 8.361429 | 2.26402 | -1.88486 | 6.26E-29 | 5.24E-28 |
| GEMIN2 | 2.167342 | 4.360995 | 1.00873 | 4.55E-16 | 1.14E-15 |
| CSAG1 | 0.022883 | 3.768316 | 7.363474 | 1.83E-07 | 2.77E-07 |
| PDCD1 | 0.939007 | 2.047047 | 1.124337 | 7.60E-07 | 1.11E-06 |
| UFSP1 | 0.896245 | 1.980025 | 1.143554 | 2.12E-14 | 4.81E-14 |
| SIGLEC14 | 5.579927 | 2.429117 | -1.19981 | 4.11E-18 | 1.18E-17 |
| PCSK9 | 7.800324 | 3.012647 | -1.3725 | 1.38E-22 | 5.73E-22 |
| UCN | 0.532874 | 1.545471 | 1.536181 | 2.44E-12 | 4.88E-12 |
| LIG1 | 2.657986 | 5.370173 | 1.014635 | 7.63E-22 | 2.94E-21 |
| SIGLEC1 | 9.339364 | 4.451622 | -1.06899 | 1.38E-18 | 4.10E-18 |
| CRYAB | 13.23142 | 3.801814 | -1.79921 | 8.20E-29 | 6.64E-28 |
| UCHL1 | 2.681411 | 33.20264 | 3.630234 | 4.38E-11 | 8.11E-11 |
| CRY2 | 16.35472 | 8.068938 | -1.01926 | 1.43E-24 | 7.22E-24 |
| ATP1A3 | 0.187525 | 0.819784 | 2.128157 | 7.02E-06 | 9.77E-06 |
| SHROOM4 | 7.614328 | 2.594255 | -1.5534 | 4.16E-26 | 2.44E-25 |
| PCOLCE2 | 10.01571 | 2.153412 | -2.21757 | 8.80E-28 | 6.21E-27 |
| GDF15 | 15.30221 | 46.21328 | 1.594568 | 5.75E-06 | 8.03E-06 |
| UBE2T | 1.749227 | 18.9699 | 3.438923 | 7.95E-35 | 9.60E-33 |
| SHOX2 | 0.043161 | 0.565342 | 3.711323 | 2.13E-17 | 5.80E-17 |
| LGSN | 0.160268 | 4.149034 | 4.694214 | 3.36E-16 | 8.52E-16 |
| UBE2S | 2.050522 | 7.475306 | 1.866141 | 2.95E-22 | 1.18E-21 |
| SHOC1 | 0.074994 | 0.759038 | 3.339327 | 5.15E-12 | 1.01E-11 |
| PCNX2 | 0.697395 | 1.419554 | 1.02539 | 2.29E-15 | 5.51E-15 |
| PCNA | 26.99732 | 64.25891 | 1.251081 | 1.43E-25 | 7.84E-25 |
| LFNG | 8.591461 | 20.09474 | 1.225842 | 2.98E-08 | 4.72E-08 |
| GCSH | 1.005736 | 2.230363 | 1.149027 | 2.18E-25 | 1.17E-24 |
| ATIC | 10.40124 | 26.55139 | 1.352032 | 4.50E-33 | 1.14E-31 |
| SHCBP1 | 0.508371 | 2.74097 | 2.430734 | 3.02E-29 | 2.65E-28 |
| PCLAF | 0.635051 | 4.965515 | 2.966998 | 8.99E-33 | 1.98E-31 |
| UBE2C | 1.60742 | 32.38577 | 4.332541 | 3.93E-33 | 1.04E-31 |
| UBD | 3.650097 | 13.54786 | 1.892059 | 6.83E-08 | 1.06E-07 |
| SHANK3 | 8.372976 | 3.396975 | -1.30149 | 6.94E-21 | 2.45E-20 |
| LDHA | 51.09425 | 141.9697 | 1.47435 | 3.71E-30 | 3.76E-29 |
| ATAD5 | 0.398642 | 1.396351 | 1.808497 | 1.13E-25 | 6.26E-25 |
| LDB2 | 16.77068 | 3.082563 | -2.44374 | 1.48E-34 | 1.34E-32 |
| ATAD3B | 1.296969 | 3.954544 | 1.608368 | 1.90E-23 | 8.44E-23 |
| ATAD2 | 1.887985 | 7.583832 | 2.00608 | 1.47E-28 | 1.14E-27 |
| TYROBP | 225.4782 | 89.20492 | -1.33779 | 3.66E-24 | 1.75E-23 |
| GATA2 | 10.00792 | 3.007957 | -1.73429 | 9.55E-25 | 4.87E-24 |
| PCDHB2 | 0.972896 | 2.163727 | 1.153161 | 0.001154 | 0.00141 |
| LCN12 | 0.152016 | 0.87849 | 2.530803 | 3.15E-17 | 8.44E-17 |
| CREB3L4 | 3.3878 | 9.650786 | 1.510298 | 5.94E-26 | 3.43E-25 |
| ASPM | 0.153025 | 2.335435 | 3.931848 | 8.32E-34 | 3.30E-32 |
| TYMS | 2.515166 | 10.35988 | 2.042282 | 3.10E-29 | 2.72E-28 |
| ASPHD1 | 0.717943 | 5.280852 | 2.878829 | 1.31E-18 | 3.91E-18 |
| SGPL1 | 7.430577 | 16.27871 | 1.131441 | 8.22E-30 | 7.80E-29 |
| PCDHB11 | 0.647607 | 1.34249 | 1.05172 | 0.011026 | 0.012728 |
| GAPDH | 216.9479 | 770.9202 | 1.829233 | 3.29E-30 | 3.36E-29 |
| ASPH | 9.562769 | 23.4697 | 1.295299 | 4.10E-08 | 6.42E-08 |
| SGO2 | 0.575351 | 1.908526 | 1.729944 | 7.90E-25 | 4.05E-24 |
| CRACR2B | 4.369826 | 9.875866 | 1.176332 | 3.16E-08 | 5.00E-08 |
| ASNS | 2.819145 | 9.826719 | 1.801452 | 1.47E-29 | 1.34E-28 |
| SGO1 | 0.126615 | 1.323622 | 3.385971 | 9.08E-33 | 1.98E-31 |
| ASIC1 | 0.266747 | 0.728786 | 1.450024 | 4.62E-10 | 8.04E-10 |
| TXNRD1 | 20.07129 | 65.82273 | 1.713452 | 1.88E-06 | 2.69E-06 |
| CPXM1 | 1.572465 | 6.917794 | 2.137284 | 7.94E-20 | 2.60E-19 |
| ASGR1 | 2.057303 | 0.988147 | -1.05796 | 1.60E-20 | 5.50E-20 |
| GALNT14 | 0.355375 | 4.954889 | 3.801437 | 6.00E-22 | 2.33E-21 |
| CPT1B | 0.182946 | 0.755402 | 2.045825 | 4.16E-18 | 1.19E-17 |
| ASF1B | 1.551262 | 8.916342 | 2.52301 | 2.07E-30 | 2.23E-29 |
| SFXN1 | 2.175719 | 6.794359 | 1.642845 | 2.14E-35 | 4.47E-33 |
| TUBB4A | 0.18755 | 1.039602 | 2.470682 | 0.007031 | 0.008202 |
| SFN | 66.35901 | 141.2371 | 1.089755 | 1.43E-12 | 2.90E-12 |
| LAPTM4B | 37.95371 | 114.7432 | 1.596096 | 1.67E-22 | 6.88E-22 |
| CPSF1 | 9.338084 | 19.25557 | 1.044078 | 1.97E-19 | 6.25E-19 |
| ASCL2 | 0.721995 | 1.907635 | 1.401724 | 0.003232 | 0.003843 |
| TUBB3 | 0.068728 | 2.033853 | 4.887168 | 4.81E-33 | 1.18E-31 |
| TUBB2B | 0.401619 | 6.897846 | 4.102245 | 1.09E-11 | 2.10E-11 |
| GAL3ST1 | 0.810766 | 1.662453 | 1.035956 | 0.004556 | 0.005375 |
| CPNE7 | 0.291363 | 2.928165 | 3.329107 | 1.44E-26 | 8.74E-26 |
| SEZ6L2 | 7.154392 | 25.86126 | 1.853891 | 5.39E-15 | 1.27E-14 |
| LAMP5 | 1.025527 | 3.282341 | 1.67836 | 2.67E-13 | 5.71E-13 |
| CPNE5 | 0.995894 | 2.583992 | 1.375537 | 4.93E-14 | 1.10E-13 |
| LAMP3 | 178.4651 | 29.03261 | -2.6199 | 4.24E-34 | 2.24E-32 |
| ASB16 | 0.392089 | 0.821709 | 1.067446 | 1.09E-11 | 2.10E-11 |
| TTYH3 | 9.530038 | 27.50918 | 1.529359 | 4.60E-24 | 2.18E-23 |
| PBK | 0.412442 | 4.809441 | 3.543607 | 6.04E-31 | 7.24E-30 |
| TTK | 0.231631 | 2.532819 | 3.450842 | 2.25E-32 | 4.06E-31 |
| PAX7 | 0.02066 | 1.927816 | 6.543985 | 4.20E-09 | 6.90E-09 |
| CPLX1 | 0.945649 | 2.219329 | 1.230746 | 8.28E-06 | 1.15E-05 |
| ARRB1 | 22.27302 | 10.20457 | -1.12608 | 2.15E-27 | 1.44E-26 |
| GABRD | 0.46155 | 1.035897 | 1.166321 | 1.60E-09 | 2.69E-09 |
| CPD | 10.18191 | 30.5531 | 1.585311 | 3.27E-18 | 9.49E-18 |
| LAGE3 | 10.17503 | 25.35642 | 1.317319 | 1.55E-23 | 7.00E-23 |
| CPA3 | 42.21663 | 14.6929 | -1.52269 | 3.72E-21 | 1.36E-20 |
| SERPINE2 | 1.435156 | 4.581334 | 1.67456 | 1.38E-09 | 2.34E-09 |
| LAG3 | 1.164886 | 3.10239 | 1.413191 | 7.56E-07 | 1.11E-06 |
| GABRA3 | 0.009543 | 0.691884 | 6.17992 | 6.62E-06 | 9.23E-06 |
| TSPAN7 | 26.77469 | 8.496117 | -1.65599 | 7.80E-26 | 4.44E-25 |
| PARPBP | 0.320105 | 1.534218 | 2.260885 | 1.49E-29 | 1.34E-28 |
| TSPAN5 | 2.044911 | 4.886913 | 1.256886 | 6.73E-14 | 1.48E-13 |
| ARL9 | 0.167542 | 0.996929 | 2.57297 | 2.84E-17 | 7.66E-17 |
| PAQR6 | 0.44897 | 1.554127 | 1.791413 | 1.12E-11 | 2.15E-11 |
| G6PD | 18.98468 | 52.21509 | 1.459631 | 1.89E-05 | 2.57E-05 |
| SERINC1 | 127.234 | 62.67066 | -1.02162 | 8.08E-34 | 3.30E-32 |
| PAQR5 | 5.610526 | 2.341574 | -1.26066 | 5.90E-25 | 3.09E-24 |
| KRTCAP2 | 3.560686 | 8.132964 | 1.191626 | 1.20E-26 | 7.39E-26 |
| PAQR4 | 2.125988 | 6.465311 | 1.604586 | 1.01E-24 | 5.13E-24 |
| PAPSS2 | 55.16704 | 21.89565 | -1.33316 | 7.38E-28 | 5.27E-27 |
| KRT80 | 2.616654 | 15.54409 | 2.570572 | 6.75E-26 | 3.89E-25 |
| PANX2 | 0.958882 | 5.663856 | 2.562359 | 5.32E-19 | 1.63E-18 |
| FZD4 | 12.68713 | 4.610279 | -1.46044 | 1.05E-25 | 5.83E-25 |
| KREMEN2 | 0.071653 | 0.614912 | 3.101277 | 3.63E-23 | 1.57E-22 |
| FZD3 | 0.528134 | 1.131639 | 1.099437 | 3.41E-09 | 5.63E-09 |
| ARHGEF39 | 0.199451 | 1.20505 | 2.59499 | 1.32E-34 | 1.32E-32 |
| TSACC | 0.088407 | 0.813501 | 3.201909 | 1.99E-32 | 3.70E-31 |
| PALMD | 5.819183 | 1.572496 | -1.88776 | 3.95E-30 | 3.98E-29 |
| KPNA7 | 0.471657 | 2.469448 | 2.388378 | 6.39E-21 | 2.28E-20 |
| ARHGEF15 | 10.08995 | 2.014127 | -2.32469 | 1.60E-33 | 5.04E-32 |
| TRPV2 | 25.84373 | 7.796905 | -1.72884 | 2.94E-32 | 4.99E-31 |
| SEMA3B | 18.93285 | 7.015993 | -1.43217 | 4.48E-23 | 1.92E-22 |
| KPNA2 | 8.407629 | 36.67205 | 2.12491 | 5.78E-29 | 4.91E-28 |
| SEMA3A | 0.764707 | 2.87094 | 1.908544 | 0.000204 | 0.000262 |
| PAFAH1B3 | 5.336094 | 27.29567 | 2.354816 | 3.20E-31 | 4.11E-30 |
| KNTC1 | 0.604595 | 2.986358 | 2.304347 | 3.56E-32 | 5.77E-31 |
| FXYD6 | 11.25427 | 3.333418 | -1.7554 | 2.22E-31 | 2.96E-30 |
| TRPM8 | 0.009097 | 1.433291 | 7.299722 | 2.60E-28 | 1.96E-27 |
| SEM1 | 3.89127 | 8.233399 | 1.081247 | 1.10E-26 | 6.80E-26 |
| KNOP1 | 1.135452 | 2.759754 | 1.281274 | 4.54E-33 | 1.15E-31 |
| COLEC12 | 18.58936 | 6.445411 | -1.52813 | 4.30E-28 | 3.17E-27 |
| ARHGAP29 | 7.613448 | 3.805338 | -1.00053 | 4.47E-21 | 1.61E-20 |
| SELPLG | 39.66056 | 16.03553 | -1.30643 | 1.55E-28 | 1.19E-27 |
| PADI3 | 0.012682 | 1.837873 | 7.179163 | 6.59E-08 | 1.02E-07 |
| KNL1 | 0.168626 | 1.176502 | 2.802607 | 3.17E-31 | 4.09E-30 |
| COLCA2 | 1.315361 | 2.637663 | 1.003802 | 0.013696 | 0.015673 |
| SELP | 10.31072 | 2.314812 | -2.15518 | 7.11E-32 | 1.04E-30 |
| PACSIN1 | 0.098224 | 0.607921 | 2.629733 | 3.24E-20 | 1.09E-19 |
| FUT8 | 3.509074 | 10.5059 | 1.582037 | 3.45E-26 | 2.04E-25 |
| COL9A2 | 1.845413 | 7.942639 | 2.105674 | 2.28E-10 | 4.04E-10 |
| TRPM2 | 1.056771 | 2.75634 | 1.383091 | 7.93E-15 | 1.85E-14 |
| PACRG | 2.194677 | 0.586447 | -1.90393 | 1.63E-17 | 4.46E-17 |
| TROAP | 0.24479 | 3.855883 | 3.977447 | 6.65E-34 | 2.97E-32 |
| SELE | 4.651574 | 0.685798 | -2.76186 | 5.67E-11 | 1.04E-10 |
| SEC61G | 18.04533 | 38.59602 | 1.096826 | 2.84E-24 | 1.37E-23 |
| KLRF1 | 2.308555 | 0.86477 | -1.4166 | 6.43E-17 | 1.69E-16 |
| COL6A3 | 15.58252 | 40.8635 | 1.390884 | 2.78E-12 | 5.54E-12 |
| PABPC1L | 1.62601 | 7.827753 | 2.267262 | 7.78E-21 | 2.74E-20 |
| FURIN | 40.01926 | 86.84622 | 1.117769 | 0.002679 | 0.003204 |
| ARHGAP11A | 0.65392 | 3.047996 | 2.220674 | 7.70E-29 | 6.35E-28 |
| PABPC1 | 152.5681 | 346.4003 | 1.182986 | 1.22E-27 | 8.42E-27 |
| TRIP13 | 1.075391 | 6.789393 | 2.658422 | 3.37E-25 | 1.79E-24 |
| SDK1 | 1.614831 | 3.969171 | 1.297455 | 0.000299 | 0.000381 |
| P4HB | 110.4562 | 258.0265 | 1.224045 | 1.09E-30 | 1.24E-29 |
| P4HA1 | 19.02591 | 39.17271 | 1.041883 | 1.13E-18 | 3.39E-18 |
| COL5A2 | 6.380411 | 32.22837 | 2.33661 | 1.92E-23 | 8.50E-23 |
| AQP9 | 11.84685 | 4.514153 | -1.39198 | 1.17E-19 | 3.78E-19 |
| SCX | 0.672707 | 2.651463 | 1.978738 | 2.71E-12 | 5.42E-12 |
| COL4A4 | 7.01825 | 3.449906 | -1.02455 | 8.15E-17 | 2.13E-16 |
| P2RY14 | 3.535964 | 1.449163 | -1.28688 | 1.57E-22 | 6.47E-22 |
| COL3A1 | 54.79556 | 352.5949 | 2.685881 | 1.64E-24 | 8.14E-24 |
| TRIM59 | 0.470102 | 1.470788 | 1.645544 | 3.39E-27 | 2.19E-26 |
| SCNN1D | 0.373187 | 1.169747 | 1.648226 | 8.33E-11 | 1.51E-10 |
| P2RX5 | 0.234778 | 0.650192 | 1.469568 | 2.24E-13 | 4.78E-13 |
| FSCN1 | 15.68135 | 33.38634 | 1.090208 | 0.000351 | 0.000444 |
| APOLD1 | 10.87657 | 3.121813 | -1.80077 | 8.66E-15 | 2.02E-14 |
| P2RX1 | 2.122445 | 1.050694 | -1.01438 | 1.29E-16 | 3.34E-16 |
| FRY | 4.73023 | 1.866153 | -1.34184 | 1.49E-28 | 1.15E-27 |
| COL1A2 | 60.07814 | 186.6002 | 1.635039 | 2.89E-13 | 6.14E-13 |
| APOL3 | 14.93982 | 6.555332 | -1.18842 | 3.45E-22 | 1.38E-21 |
| TRIM17 | 0.336048 | 1.642084 | 2.288787 | 5.09E-10 | 8.82E-10 |
| COL1A1 | 41.03804 | 338.6485 | 3.044755 | 4.64E-25 | 2.44E-24 |
| SCIN | 1.399136 | 4.432749 | 1.663665 | 0.000408 | 0.000515 |
| KLHL35 | 0.468068 | 1.262762 | 1.431792 | 2.86E-07 | 4.29E-07 |
| FRMD4A | 2.349142 | 1.062821 | -1.14424 | 2.90E-27 | 1.90E-26 |
| APOC1 | 204.8719 | 85.49405 | -1.26083 | 2.61E-18 | 7.62E-18 |
| FRMD3 | 2.980801 | 0.617503 | -2.27118 | 6.29E-32 | 9.35E-31 |
| COL11A2 | 0.122639 | 0.890318 | 2.859908 | 0.001118 | 0.00137 |
| FRK | 1.491557 | 3.2075 | 1.10463 | 3.57E-16 | 9.04E-16 |
| COCH | 0.144958 | 2.130686 | 3.877613 | 1.67E-20 | 5.70E-20 |
| APOBEC3A | 1.735969 | 0.791345 | -1.13336 | 1.16E-11 | 2.22E-11 |
| SCG5 | 0.776406 | 6.007862 | 2.951969 | 8.02E-21 | 2.82E-20 |
| KLHL17 | 1.048681 | 3.103993 | 1.565549 | 1.32E-21 | 5.01E-21 |
| COA6 | 8.563693 | 20.88509 | 1.286169 | 5.61E-25 | 2.94E-24 |
| SCG2 | 0.924062 | 9.14828 | 3.30744 | 0.018332 | 0.020758 |
| FPR2 | 6.190164 | 0.930407 | -2.73404 | 5.81E-30 | 5.64E-29 |
| APLN | 12.10702 | 4.227025 | -1.51813 | 2.76E-15 | 6.62E-15 |
| FPR1 | 18.99579 | 5.684836 | -1.74049 | 2.37E-21 | 8.78E-21 |
| KLF9 | 32.34433 | 11.39949 | -1.50454 | 4.10E-24 | 1.96E-23 |
| FOXRED2 | 2.870355 | 6.015661 | 1.067494 | 1.09E-12 | 2.23E-12 |
| APBA2 | 0.686626 | 3.031062 | 2.142226 | 2.17E-13 | 4.65E-13 |
| SCAMP5 | 1.4971 | 3.853065 | 1.363836 | 6.97E-17 | 1.83E-16 |
| ORC6 | 0.21395 | 1.948602 | 3.187093 | 3.07E-34 | 1.91E-32 |
| FOXP3 | 0.604574 | 2.389851 | 1.98293 | 1.55E-23 | 7.00E-23 |
| CNR1 | 2.43322 | 0.965062 | -1.33417 | 9.45E-19 | 2.85E-18 |
| ORC1 | 0.339719 | 2.544052 | 2.904713 | 6.11E-32 | 9.14E-31 |
| TRAIP | 0.442935 | 1.714062 | 1.952252 | 5.77E-32 | 8.72E-31 |
| SBK1 | 1.087408 | 7.00331 | 2.687143 | 4.93E-21 | 1.77E-20 |
| KISS1R | 0.014309 | 1.185566 | 6.372512 | 9.15E-31 | 1.06E-29 |
| FOXM1 | 0.682724 | 7.856073 | 3.524434 | 3.93E-33 | 1.04E-31 |
| CNN1 | 12.2086 | 4.817645 | -1.3415 | 3.12E-18 | 9.04E-18 |
| TRAF5 | 1.220595 | 3.42116 | 1.486901 | 5.49E-21 | 1.96E-20 |
| KISS1 | 0.314317 | 1.385279 | 2.139883 | 1.67E-07 | 2.54E-07 |
| AP1S1 | 16.99382 | 40.29081 | 1.24544 | 9.69E-28 | 6.76E-27 |
| TRAF4 | 9.339556 | 25.83759 | 1.468046 | 5.45E-31 | 6.57E-30 |
| FOXI3 | 0.009378 | 1.176205 | 6.97071 | 4.15E-16 | 1.05E-15 |
| SASS6 | 0.824787 | 2.135726 | 1.372633 | 5.68E-24 | 2.65E-23 |
| AOX1 | 4.598798 | 2.219168 | -1.05124 | 8.86E-19 | 2.68E-18 |
| AOC1 | 0.343255 | 11.60149 | 5.078884 | 9.26E-17 | 2.42E-16 |
| TRAF2 | 4.225668 | 9.311616 | 1.139852 | 9.70E-23 | 4.05E-22 |
| SARS2 | 0.975459 | 2.015608 | 1.047062 | 9.93E-19 | 2.99E-18 |
| CMTM1 | 0.374649 | 0.936498 | 1.321737 | 3.44E-20 | 1.15E-19 |
| SAPCD2 | 0.308466 | 4.67058 | 3.92042 | 1.75E-35 | 4.47E-33 |
| TPX2 | 1.23093 | 17.25718 | 3.809377 | 2.45E-33 | 7.32E-32 |
| ANP32E | 15.03827 | 36.68843 | 1.286687 | 2.97E-18 | 8.61E-18 |
| SAMD10 | 1.542031 | 5.588946 | 1.857744 | 6.60E-30 | 6.32E-29 |
| KIFC2 | 1.300877 | 4.916524 | 1.918154 | 1.33E-17 | 3.68E-17 |
| ANOS1 | 31.36189 | 5.022872 | -2.64243 | 3.55E-34 | 2.00E-32 |
| KIFC1 | 1.107668 | 8.277815 | 2.901724 | 2.06E-30 | 2.22E-29 |
| ANO9 | 1.919714 | 6.702737 | 1.803859 | 5.55E-19 | 1.70E-18 |
| TPSB2 | 35.21107 | 10.92155 | -1.68885 | 1.89E-19 | 5.99E-19 |
| OLR1 | 62.38426 | 12.08486 | -2.36798 | 9.44E-32 | 1.33E-30 |
| CLSPN | 0.209081 | 1.567854 | 2.906659 | 9.99E-29 | 8.00E-28 |
| ANO7 | 0.159368 | 0.645026 | 2.016994 | 9.46E-29 | 7.61E-28 |
| TPSAB1 | 31.20318 | 10.37117 | -1.58911 | 1.31E-23 | 5.94E-23 |
| KIF4A | 0.298841 | 4.779645 | 3.999454 | 3.84E-34 | 2.07E-32 |
| CLPTM1L | 21.47807 | 45.62634 | 1.087002 | 3.58E-23 | 1.55E-22 |
| SAC3D1 | 3.052504 | 6.50504 | 1.091565 | 7.00E-22 | 2.71E-21 |
| KIF3C | 1.455313 | 3.415986 | 1.230973 | 5.56E-08 | 8.67E-08 |
| CLPSL2 | 0.018986 | 0.972862 | 5.679246 | 1.24E-23 | 5.63E-23 |
| ANLN | 0.561131 | 8.672361 | 3.950017 | 6.09E-34 | 2.80E-32 |
| KIF2C | 0.527979 | 7.03659 | 3.736325 | 1.93E-34 | 1.63E-32 |
| FOXA1 | 7.609689 | 22.00473 | 1.531904 | 7.50E-16 | 1.86E-15 |
| S1PR1 | 51.13113 | 7.960642 | -2.68325 | 3.93E-35 | 7.12E-33 |
| OIP5 | 0.405346 | 2.315872 | 2.514329 | 5.55E-30 | 5.46E-29 |
| KIF26B | 0.371345 | 3.63609 | 3.291557 | 1.05E-31 | 1.46E-30 |
| TPI1 | 90.79643 | 214.2464 | 1.238564 | 2.81E-28 | 2.10E-27 |
| S100B | 2.889576 | 7.670621 | 1.408486 | 0.000323 | 0.000409 |
| FOSB | 164.7978 | 18.92101 | -3.12264 | 3.41E-20 | 1.14E-19 |
| TPBG | 1.842372 | 6.714161 | 1.865643 | 3.93E-27 | 2.52E-26 |
| OGDHL | 0.054278 | 0.690515 | 3.669227 | 4.83E-10 | 8.40E-10 |
| KIF23 | 0.47961 | 3.178824 | 2.728559 | 3.17E-30 | 3.25E-29 |
| S100A5 | 0.317072 | 1.288691 | 2.023023 | 0.001196 | 0.001458 |
| ANKRD34B | 0.018364 | 0.712086 | 5.277064 | 6.82E-13 | 1.42E-12 |
| KIF20A | 0.541053 | 5.360974 | 3.308652 | 9.18E-34 | 3.45E-32 |
| S100A12 | 5.26648 | 1.451438 | -1.85936 | 2.75E-19 | 8.63E-19 |
| KIF1A | 0.069967 | 3.317511 | 5.567285 | 0.004002 | 0.004742 |
| FLVCR2 | 7.754972 | 3.575618 | -1.11693 | 2.02E-25 | 1.09E-24 |
| CLIC6 | 16.40938 | 41.91268 | 1.352866 | 0.0001 | 0.000131 |
| KIF18B | 0.200138 | 3.070484 | 3.939397 | 3.67E-33 | 9.87E-32 |
| FLT4 | 5.512186 | 2.359697 | -1.22402 | 1.75E-20 | 6.00E-20 |
| OCIAD2 | 9.711724 | 47.84953 | 2.300705 | 3.59E-34 | 2.00E-32 |
| KIF18A | 0.589381 | 1.704394 | 1.531986 | 1.45E-20 | 5.01E-20 |
| RXRG | 2.490992 | 0.535381 | -2.21808 | 1.06E-28 | 8.48E-28 |
| OAS3 | 8.345689 | 16.80138 | 1.009477 | 4.12E-09 | 6.78E-09 |
| KIF15 | 0.238205 | 1.717839 | 2.850321 | 1.58E-31 | 2.16E-30 |
| FLI1 | 8.150835 | 2.860781 | -1.51054 | 8.93E-31 | 1.04E-29 |
| KIF14 | 0.100845 | 1.546874 | 3.939149 | 8.41E-34 | 3.30E-32 |
| FLAD1 | 4.969548 | 14.33484 | 1.528339 | 1.06E-33 | 3.76E-32 |
| OAS1 | 8.033465 | 20.1677 | 1.327952 | 3.97E-12 | 7.84E-12 |
| TOP2A | 1.273108 | 20.86272 | 4.0345 | 4.34E-35 | 7.13E-33 |
| RUNX2 | 0.764265 | 2.622581 | 1.778842 | 2.29E-24 | 1.12E-23 |
| NXPH4 | 0.141485 | 2.885964 | 4.350329 | 5.57E-20 | 1.84E-19 |
| FKBP4 | 10.12017 | 24.29439 | 1.26339 | 1.29E-22 | 5.35E-22 |
| TOP1MT | 2.609473 | 5.363497 | 1.039416 | 6.84E-21 | 2.42E-20 |
| KIF11 | 0.827976 | 5.940285 | 2.842872 | 9.63E-34 | 3.54E-32 |
| TONSL | 0.730551 | 3.503725 | 2.261834 | 6.98E-32 | 1.03E-30 |
| FIBIN | 18.40249 | 4.632205 | -1.99013 | 4.85E-26 | 2.82E-25 |
| NUSAP1 | 1.706817 | 11.2693 | 2.723018 | 9.17E-33 | 1.98E-31 |
| FHL2 | 1.894296 | 10.55126 | 2.477681 | 1.91E-24 | 9.40E-24 |
| CLEC1A | 4.241684 | 0.953415 | -2.15346 | 8.32E-34 | 3.30E-32 |
| RTKN2 | 29.83703 | 2.004473 | -3.89581 | 5.67E-35 | 8.18E-33 |
| NUP85 | 4.603102 | 9.377494 | 1.026596 | 1.13E-29 | 1.04E-28 |
| FGR | 25.32132 | 6.639022 | -1.93131 | 5.10E-32 | 7.88E-31 |
| TNFSF4 | 0.525435 | 1.376967 | 1.389908 | 4.95E-13 | 1.04E-12 |
| KHDC4 | 3.532188 | 10.88714 | 1.623991 | 1.39E-28 | 1.08E-27 |
| CLEC14A | 48.8876 | 10.92271 | -2.16214 | 5.35E-33 | 1.27E-31 |
| NUP210 | 5.516818 | 13.18473 | 1.25696 | 3.34E-18 | 9.68E-18 |
| KHDC1L | 0.013164 | 0.646305 | 5.617526 | 2.10E-09 | 3.51E-09 |
| FGFR4 | 16.10981 | 3.229638 | -2.3185 | 1.38E-30 | 1.54E-29 |
| NUP155 | 2.397755 | 6.699883 | 1.482452 | 2.84E-30 | 2.95E-29 |
| FGFBP2 | 5.13852 | 0.696803 | -2.88253 | 5.06E-31 | 6.17E-30 |
| CLDN9 | 0.806395 | 4.054372 | 2.329921 | 1.02E-05 | 1.41E-05 |
| AMH | 0.147957 | 0.552795 | 1.901568 | 0.016883 | 0.019169 |
| CLDN5 | 45.08863 | 7.497093 | -2.58836 | 3.06E-32 | 5.15E-31 |
| TNFSF13 | 20.37976 | 9.728411 | -1.06686 | 1.49E-28 | 1.15E-27 |
| NUF2 | 0.285065 | 4.163687 | 3.8685 | 5.69E-34 | 2.70E-32 |
| ALPL | 87.7183 | 40.33608 | -1.12081 | 2.80E-18 | 8.16E-18 |
| TNFRSF9 | 0.447006 | 1.305416 | 1.546145 | 3.71E-11 | 6.90E-11 |
| RRM2 | 1.112912 | 11.59455 | 3.381036 | 3.99E-32 | 6.36E-31 |
| NUDT8 | 2.26482 | 6.167662 | 1.445327 | 1.38E-22 | 5.73E-22 |
| CLDN11 | 2.102464 | 0.977596 | -1.10477 | 1.58E-22 | 6.52E-22 |
| NUDT1 | 3.433647 | 7.415442 | 1.110791 | 6.44E-21 | 2.29E-20 |
| ALOX5AP | 88.15452 | 25.56641 | -1.78578 | 7.92E-28 | 5.60E-27 |
| RPUSD1 | 4.494793 | 9.492594 | 1.078548 | 1.36E-25 | 7.47E-25 |
| KCP | 0.127583 | 0.627602 | 2.29841 | 7.33E-09 | 1.19E-08 |
| FGD5 | 11.8214 | 2.734526 | -2.11204 | 4.91E-34 | 2.46E-32 |
| ALOX5 | 46.25524 | 14.52733 | -1.67085 | 5.93E-29 | 5.03E-28 |
| TNFRSF25 | 1.011619 | 4.172716 | 2.044321 | 2.96E-20 | 9.97E-20 |
| ALOX15 | 10.25568 | 2.588698 | -1.98613 | 1.07E-12 | 2.19E-12 |
| CKS2 | 21.39692 | 47.77808 | 1.158946 | 1.43E-11 | 2.74E-11 |
| ALKBH6 | 0.387411 | 0.870738 | 1.168375 | 9.49E-16 | 2.34E-15 |
| RPS6KL1 | 0.315047 | 0.636018 | 1.013499 | 2.64E-05 | 3.56E-05 |
| FEZ1 | 4.9644 | 1.239622 | -2.00172 | 3.53E-33 | 9.77E-32 |
| CKS1B | 3.675147 | 13.28974 | 1.854439 | 1.42E-29 | 1.29E-28 |
| TNFRSF18 | 0.954042 | 6.548463 | 2.779032 | 1.77E-23 | 7.90E-23 |
| KCNQ3 | 0.71973 | 3.220715 | 2.161853 | 1.86E-16 | 4.78E-16 |
| ALG8 | 6.039664 | 12.54512 | 1.054587 | 1.25E-31 | 1.71E-30 |
| TNFRSF17 | 0.966901 | 3.862109 | 1.997949 | 5.76E-12 | 1.13E-11 |
| CKAP4 | 19.27516 | 44.61861 | 1.210902 | 3.10E-25 | 1.65E-24 |
| ALG1L | 0.813759 | 8.508142 | 3.386171 | 4.44E-21 | 1.60E-20 |
| FERMT1 | 0.625695 | 6.352161 | 3.343715 | 1.86E-32 | 3.53E-31 |
| CKAP2L | 0.22467 | 2.257505 | 3.328849 | 2.07E-32 | 3.80E-31 |
| NT5DC3 | 1.030263 | 2.102626 | 1.029179 | 4.39E-14 | 9.77E-14 |
| FEN1 | 4.325888 | 14.41629 | 1.736632 | 3.11E-30 | 3.20E-29 |
| CKAP2 | 2.684483 | 6.377609 | 1.248371 | 2.56E-21 | 9.42E-21 |
| ALDOA | 94.92509 | 234.5513 | 1.305042 | 4.72E-27 | 2.99E-26 |
| TNFRSF11A | 0.496484 | 1.257371 | 1.340591 | 1.22E-12 | 2.49E-12 |
| KCNK5 | 5.995334 | 18.55503 | 1.629898 | 2.04E-14 | 4.64E-14 |
| CIT | 2.55636 | 11.37424 | 2.153607 | 1.55E-10 | 2.78E-10 |
| ALDH1B1 | 6.133906 | 14.22436 | 1.213486 | 1.15E-21 | 4.38E-21 |
| NSUN5 | 4.55092 | 9.785517 | 1.10449 | 1.81E-27 | 1.21E-26 |
| KCNJ15 | 13.77493 | 5.898916 | -1.22352 | 2.18E-19 | 6.88E-19 |
| CIP2A | 0.528737 | 2.316805 | 2.131514 | 1.46E-24 | 7.31E-24 |
| RPL39L | 2.823828 | 15.65095 | 2.470526 | 6.22E-24 | 2.89E-23 |
| NSMF | 4.647903 | 12.21702 | 1.39424 | 3.45E-26 | 2.04E-25 |
| FCRLA | 0.640229 | 1.49057 | 1.219205 | 3.98E-11 | 7.38E-11 |
| CHTF18 | 0.829133 | 3.200743 | 1.94873 | 8.93E-26 | 5.03E-25 |
| TMSB15A | 3.608169 | 1.320513 | -1.45017 | 4.01E-19 | 1.25E-18 |
| AKR1E2 | 0.570655 | 1.553605 | 1.44493 | 1.40E-09 | 2.36E-09 |
| FCRL5 | 0.205713 | 1.392416 | 2.758884 | 1.95E-18 | 5.77E-18 |
| CHST15 | 5.972516 | 13.26686 | 1.151416 | 3.69E-18 | 1.06E-17 |
| AKR1B10 | 0.273728 | 50.59487 | 7.530102 | 3.24E-14 | 7.28E-14 |
| ROBO4 | 20.09393 | 3.107478 | -2.69294 | 3.19E-34 | 1.91E-32 |
| FCRL2 | 0.213026 | 0.914553 | 2.102038 | 1.85E-11 | 3.50E-11 |
| NRIP3 | 0.69062 | 2.673284 | 1.952648 | 5.95E-10 | 1.03E-09 |
| CHRNA5 | 0.114073 | 1.5188 | 3.734903 | 3.77E-28 | 2.78E-27 |
| RNFT2 | 0.219445 | 1.123044 | 2.35548 | 2.09E-24 | 1.03E-23 |
| FCN1 | 8.295384 | 2.791797 | -1.57111 | 4.17E-20 | 1.39E-19 |
| RNF43 | 2.380922 | 6.006443 | 1.33499 | 2.82E-17 | 7.63E-17 |
| AIM2 | 0.743315 | 5.769133 | 2.956308 | 7.49E-15 | 1.75E-14 |
| FCGR3B | 4.1118 | 0.910605 | -2.17487 | 4.07E-18 | 1.17E-17 |
| CHML | 2.417797 | 6.714104 | 1.473501 | 6.00E-14 | 1.33E-13 |
| RNF207 | 0.694361 | 2.240072 | 1.689788 | 6.46E-18 | 1.84E-17 |
| FCGR3A | 84.5669 | 39.00172 | -1.11656 | 8.36E-20 | 2.72E-19 |
| CHL1 | 1.050455 | 3.367791 | 1.680788 | 0.001141 | 0.001395 |
| TMEM63C | 0.119429 | 2.14531 | 4.166956 | 1.60E-20 | 5.50E-20 |
| KBTBD12 | 0.170125 | 0.786875 | 2.209537 | 0.000836 | 0.001033 |
| NR4A3 | 10.51354 | 2.472052 | -2.08847 | 3.04E-17 | 8.17E-17 |
| CHI3L2 | 17.82514 | 5.874345 | -1.60141 | 1.00E-19 | 3.25E-19 |
| RNF183 | 0.057081 | 1.074036 | 4.233883 | 1.36E-14 | 3.14E-14 |
| KAZALD1 | 1.431891 | 2.960511 | 1.047925 | 6.63E-09 | 1.08E-08 |
| CHI3L1 | 21.31106 | 61.65104 | 1.532523 | 0.000567 | 0.000707 |
| NR2F6 | 11.24739 | 23.73529 | 1.077443 | 1.40E-22 | 5.77E-22 |
| CHEK2 | 1.240853 | 3.807431 | 1.617485 | 7.44E-28 | 5.31E-27 |
| AHNAK | 124.7919 | 36.48568 | -1.77412 | 6.94E-28 | 4.98E-27 |
| RNF144B | 20.56191 | 7.177311 | -1.51846 | 2.10E-31 | 2.81E-30 |
| CHEK1 | 0.535429 | 2.816161 | 2.394962 | 1.05E-32 | 2.20E-31 |
| FCER1G | 123.0034 | 57.95699 | -1.08564 | 9.12E-22 | 3.49E-21 |
| KANK2 | 32.60283 | 11.51653 | -1.50129 | 9.00E-32 | 1.29E-30 |
| CHD1L | 9.769551 | 20.37851 | 1.060684 | 7.77E-29 | 6.38E-28 |
| CHAF1B | 0.668851 | 2.810356 | 2.070996 | 7.85E-34 | 3.29E-32 |
| NQO1 | 11.45179 | 99.72122 | 3.122328 | 2.77E-23 | 1.22E-22 |
| CHAF1A | 2.063077 | 4.623187 | 1.16409 | 4.05E-21 | 1.47E-20 |
| RNASEH2A | 4.295015 | 11.56527 | 1.429064 | 8.44E-25 | 4.32E-24 |
| NPTX2 | 0.438613 | 2.841026 | 2.69539 | 6.48E-08 | 1.01E-07 |
| FBXO5 | 1.290247 | 2.634298 | 1.029771 | 2.08E-14 | 4.73E-14 |
| CHAD | 0.835295 | 3.059928 | 1.87314 | 0.001251 | 0.001522 |
| NPR1 | 15.71264 | 2.851762 | -2.462 | 2.40E-33 | 7.25E-32 |
| FBXO41 | 0.559389 | 2.028234 | 1.8583 | 7.52E-23 | 3.16E-22 |
| CFTR | 5.337598 | 2.554318 | -1.06325 | 1.05E-16 | 2.75E-16 |
| AFF3 | 3.021428 | 0.639913 | -2.23928 | 8.85E-30 | 8.33E-29 |
| TMEM198 | 0.757067 | 1.597056 | 1.076923 | 2.72E-08 | 4.32E-08 |
| CFP | 3.112275 | 0.827749 | -1.9107 | 3.26E-31 | 4.17E-30 |
| TMEM178A | 2.92144 | 1.434639 | -1.02599 | 7.66E-21 | 2.70E-20 |
| NPIPB15 | 0.706517 | 1.974775 | 1.482892 | 0.000252 | 0.000323 |
| AFAP1L1 | 8.413644 | 2.851212 | -1.56116 | 6.94E-28 | 4.98E-27 |
| RMI2 | 1.131555 | 6.147781 | 2.441759 | 2.64E-33 | 7.83E-32 |
| FBXL8 | 1.494992 | 2.996498 | 1.00314 | 5.96E-12 | 1.16E-11 |
| TMEM156 | 0.491596 | 2.980489 | 2.600005 | 5.02E-20 | 1.66E-19 |
| ITM2A | 34.2883 | 9.331982 | -1.87746 | 6.08E-30 | 5.86E-29 |
| FBXL6 | 3.083034 | 7.633246 | 1.307946 | 4.20E-24 | 2.00E-23 |
| ADRB2 | 12.79609 | 1.714631 | -2.89973 | 1.22E-35 | 4.47E-33 |
| RIPPLY3 | 0.345547 | 1.021313 | 1.563472 | 0.012464 | 0.014297 |
| CERS6 | 2.445302 | 5.373543 | 1.135861 | 6.32E-19 | 1.93E-18 |
| FBXL16 | 1.233965 | 3.195366 | 1.37268 | 0.029096 | 0.032452 |
| ADGRL4 | 11.04275 | 5.030526 | -1.13432 | 2.46E-20 | 8.31E-20 |
| TMEM132A | 1.927271 | 8.681273 | 2.171347 | 1.08E-23 | 4.92E-23 |
| ITGB8 | 1.077736 | 3.333082 | 1.628853 | 0.005186 | 0.006095 |
| RILPL2 | 9.660828 | 4.309792 | -1.16453 | 5.15E-33 | 1.23E-31 |
| NOXA1 | 3.330621 | 9.423916 | 1.500535 | 8.55E-15 | 2.00E-14 |
| CEP72 | 0.964149 | 2.620686 | 1.442616 | 5.17E-25 | 2.71E-24 |
| ADGRG1 | 7.915722 | 22.89671 | 1.532348 | 8.87E-20 | 2.88E-19 |
| NOX4 | 0.344121 | 0.952887 | 1.469389 | 2.16E-12 | 4.34E-12 |
| ITGB4 | 11.01898 | 37.56465 | 1.769385 | 8.66E-12 | 1.68E-11 |
| RHPN1 | 2.359851 | 9.593977 | 2.023433 | 3.76E-22 | 1.49E-21 |
| FBLN5 | 32.84415 | 10.46268 | -1.65038 | 3.84E-30 | 3.89E-29 |
| ADGRE5 | 51.83579 | 23.54502 | -1.13853 | 8.57E-27 | 5.35E-26 |
| NOX1 | 0.302054 | 0.898188 | 1.572211 | 1.46E-17 | 4.01E-17 |
| FBLN1 | 55.57887 | 24.35508 | -1.19031 | 7.84E-19 | 2.38E-18 |
| CEP55 | 0.722848 | 7.684068 | 3.410107 | 5.84E-33 | 1.35E-31 |
| ADGRE3 | 2.130072 | 0.334822 | -2.66944 | 1.12E-28 | 8.94E-28 |
| RHOJ | 9.821133 | 3.00171 | -1.7101 | 1.13E-31 | 1.57E-30 |
| CEP131 | 3.001683 | 6.082487 | 1.01889 | 1.54E-15 | 3.75E-15 |
| ADGRE1 | 3.965883 | 0.668389 | -2.56888 | 4.53E-29 | 3.91E-28 |
| ITGAL | 13.57204 | 6.574239 | -1.04574 | 2.62E-19 | 8.22E-19 |
| CENPW | 3.143368 | 9.639613 | 1.616664 | 4.35E-16 | 1.10E-15 |
| ADGRB2 | 0.319999 | 1.01388 | 1.663747 | 7.16E-13 | 1.48E-12 |
| CENPO | 1.117795 | 2.526126 | 1.176271 | 8.47E-24 | 3.89E-23 |
| ADGRB1 | 0.181678 | 1.753828 | 3.271052 | 1.46E-06 | 2.11E-06 |
| CENPN | 1.284996 | 3.173989 | 1.304533 | 7.94E-20 | 2.60E-19 |
| TM6SF1 | 3.784514 | 1.593992 | -1.24746 | 4.89E-26 | 2.84E-25 |
| FAP | 0.667199 | 3.573563 | 2.421173 | 6.86E-25 | 3.55E-24 |
| CENPM | 1.384592 | 5.015736 | 1.857 | 5.63E-22 | 2.19E-21 |
| RHBDD3 | 3.919693 | 8.166937 | 1.059054 | 6.69E-22 | 2.59E-21 |
| ITGA11 | 0.565922 | 4.555858 | 3.009047 | 3.10E-25 | 1.65E-24 |
| FANCI | 0.978002 | 4.305453 | 2.138255 | 2.86E-33 | 8.29E-32 |
| CENPL | 0.678513 | 2.014214 | 1.569768 | 3.90E-31 | 4.92E-30 |
| ADCY9 | 9.174169 | 4.586333 | -1.00024 | 1.76E-21 | 6.60E-21 |
| FANCG | 2.062149 | 5.29222 | 1.359725 | 4.91E-29 | 4.23E-28 |
| CENPK | 0.340204 | 1.799776 | 2.403346 | 3.33E-29 | 2.90E-28 |
| ADCY4 | 3.459269 | 1.122263 | -1.62406 | 1.62E-26 | 9.82E-26 |
| CENPI | 0.185364 | 1.39141 | 2.908111 | 5.60E-30 | 5.47E-29 |
| RGS20 | 0.085075 | 0.689488 | 3.018713 | 1.44E-15 | 3.51E-15 |
| NOL3 | 7.098652 | 14.36838 | 1.01728 | 3.97E-17 | 1.05E-16 |
| FANCD2 | 0.639995 | 2.071104 | 1.694267 | 2.15E-30 | 2.30E-29 |
| CENPH | 1.378024 | 4.092731 | 1.570463 | 9.69E-28 | 6.76E-27 |
| ADCK5 | 1.598526 | 5.474292 | 1.77593 | 1.44E-27 | 9.85E-27 |
| CENPF | 0.40657 | 5.128707 | 3.65702 | 1.71E-33 | 5.28E-32 |
| TLR8 | 5.83755 | 1.912064 | -1.61023 | 4.60E-25 | 2.42E-24 |
| RGS17 | 0.116959 | 1.716094 | 3.87505 | 2.23E-28 | 1.70E-27 |
| ISG15 | 33.23618 | 106.8073 | 1.684184 | 6.48E-11 | 1.19E-10 |
| FANCA | 0.436765 | 1.794461 | 2.038621 | 3.93E-31 | 4.94E-30 |
| CENPE | 0.211146 | 1.505078 | 2.833525 | 1.01E-29 | 9.44E-29 |
| CENPA | 0.255685 | 3.531929 | 3.788019 | 5.06E-32 | 7.88E-31 |
| FAM90A1 | 0.273584 | 0.842072 | 1.62196 | 6.58E-07 | 9.68E-07 |
| ADARB1 | 7.3333 | 2.417356 | -1.60103 | 1.28E-27 | 8.81E-27 |
| TLR10 | 0.44719 | 1.155137 | 1.369104 | 1.11E-09 | 1.89E-09 |
| CEMIP | 2.207179 | 10.13753 | 2.19943 | 1.47E-11 | 2.80E-11 |
| CELSR3 | 0.124799 | 1.437725 | 3.52611 | 6.59E-32 | 9.76E-31 |
| CELF3 | 0.033987 | 0.840232 | 4.627747 | 0.000154 | 0.000199 |
| TLCD1 | 4.773114 | 17.57054 | 1.880156 | 7.84E-29 | 6.41E-28 |
| IRF7 | 8.093505 | 21.56299 | 1.413721 | 1.57E-23 | 7.04E-23 |
| CEL | 0.195746 | 1.900246 | 3.279134 | 0.000673 | 0.000835 |
| RGCC | 296.4727 | 45.12487 | -2.7159 | 2.05E-35 | 4.47E-33 |
| IRF4 | 1.298383 | 2.803707 | 1.11062 | 0.001379 | 0.001673 |
| TK1 | 5.003963 | 34.57339 | 2.788519 | 5.11E-30 | 5.06E-29 |
| CEACAM6 | 208.0287 | 600.2231 | 1.528716 | 8.49E-08 | 1.31E-07 |
| ADAMDEC1 | 1.062666 | 5.625023 | 2.404171 | 5.47E-14 | 1.21E-13 |
| RFC5 | 2.85831 | 6.009168 | 1.072003 | 2.15E-25 | 1.16E-24 |
| IQGAP3 | 0.366239 | 5.085683 | 3.795584 | 1.37E-34 | 1.32E-32 |
| ADAM8 | 3.211078 | 15.45878 | 2.267297 | 2.70E-24 | 1.31E-23 |
| RFC4 | 1.789183 | 6.798425 | 1.9259 | 6.81E-33 | 1.57E-31 |
| CDT1 | 0.734409 | 5.414193 | 2.882091 | 1.10E-29 | 1.02E-28 |
| TIMM8A | 1.244792 | 3.16376 | 1.345736 | 3.70E-32 | 5.96E-31 |
| RFC3 | 2.598078 | 5.44099 | 1.066425 | 1.64E-18 | 4.85E-18 |
| FAM43A | 7.092136 | 3.477527 | -1.02816 | 5.40E-16 | 1.35E-15 |
| CDKN3 | 0.655207 | 5.414103 | 3.0467 | 1.11E-29 | 1.03E-28 |
| IPCEF1 | 0.640557 | 1.630311 | 1.347749 | 0.003368 | 0.004001 |
| FAM3C | 8.524795 | 22.00807 | 1.368296 | 5.59E-22 | 2.18E-21 |
| ADAM28 | 0.82597 | 4.421899 | 2.420504 | 4.83E-24 | 2.28E-23 |
| RETREG1 | 7.373706 | 3.083891 | -1.25764 | 3.97E-22 | 1.57E-21 |
| INTS8 | 2.56541 | 6.394457 | 1.317632 | 1.03E-32 | 2.18E-31 |
| FAM3B | 4.088546 | 8.693209 | 1.088301 | 0.044312 | 0.048804 |
| CDKN2A | 0.498778 | 6.306165 | 3.660292 | 1.45E-14 | 3.33E-14 |
| TIMELESS | 2.385667 | 8.681096 | 1.863485 | 8.65E-33 | 1.93E-31 |
| INTS7 | 3.379315 | 6.818607 | 1.012746 | 9.69E-28 | 6.76E-27 |
| FAM24B | 0.590561 | 1.603515 | 1.441081 | 1.22E-21 | 4.65E-21 |
| RET | 0.249382 | 2.093543 | 3.069514 | 0.008169 | 0.009498 |
| ADAM15 | 17.42432 | 36.81897 | 1.079347 | 6.84E-21 | 2.42E-20 |
| TIGIT | 0.66598 | 1.68528 | 1.339436 | 1.10E-10 | 1.98E-10 |
| INSYN2A | 0.090763 | 0.56705 | 2.643294 | 5.15E-07 | 7.63E-07 |
| CDK5RAP3 | 9.165958 | 20.10076 | 1.132893 | 9.13E-20 | 2.96E-19 |
| ADAM12 | 0.337435 | 3.245506 | 3.265761 | 5.64E-24 | 2.63E-23 |
| ACVRL1 | 35.97117 | 6.714144 | -2.42157 | 1.97E-34 | 1.63E-32 |
| TIGD3 | 0.191821 | 0.592217 | 1.626365 | 4.85E-18 | 1.39E-17 |
| CDK5R1 | 0.49372 | 1.592183 | 1.689241 | 6.69E-21 | 2.37E-20 |
| TIE1 | 14.52682 | 4.059272 | -1.83943 | 9.90E-32 | 1.39E-30 |
| TICRR | 0.089273 | 0.756087 | 3.082263 | 4.79E-31 | 5.88E-30 |
| THY1 | 5.818969 | 16.1184 | 1.469873 | 5.03E-16 | 1.26E-15 |
| FAM178B | 0.117164 | 0.943402 | 3.009341 | 5.40E-19 | 1.65E-18 |
| INPP4B | 1.152951 | 2.520874 | 1.128593 | 3.92E-07 | 5.84E-07 |
| FAM171A2 | 0.405678 | 1.751968 | 2.110569 | 1.03E-12 | 2.12E-12 |
| ACTL6A | 8.008319 | 16.97738 | 1.084042 | 9.53E-28 | 6.69E-27 |
| REG4 | 0.010883 | 16.56766 | 10.57209 | 2.78E-06 | 3.96E-06 |
| INKA2 | 1.783384 | 0.662287 | -1.42909 | 2.73E-32 | 4.65E-31 |
| ACTL10 | 0.553002 | 1.385314 | 1.324856 | 7.45E-12 | 1.45E-11 |
| RECQL4 | 0.69834 | 6.271637 | 3.166841 | 4.68E-34 | 2.40E-32 |
| CDK1 | 1.559918 | 9.505669 | 2.607318 | 3.27E-29 | 2.86E-28 |
| NFE2L3 | 4.144887 | 12.07976 | 1.543187 | 2.19E-18 | 6.43E-18 |
| INA | 0.067552 | 1.259496 | 4.220707 | 2.36E-09 | 3.95E-09 |
| FAM136A | 6.65264 | 16.54725 | 1.314593 | 1.58E-35 | 4.47E-33 |
| ACSS3 | 2.420013 | 0.877933 | -1.46283 | 4.38E-28 | 3.22E-27 |
| NFATC4 | 1.961348 | 4.995465 | 1.348773 | 1.95E-17 | 5.31E-17 |
| FAM133A | 0.030404 | 1.564271 | 5.685097 | 0.000309 | 0.000392 |
| FAM124B | 1.592634 | 0.49584 | -1.68347 | 8.51E-25 | 4.35E-24 |
| CDH5 | 42.53584 | 8.024167 | -2.40626 | 2.81E-34 | 1.91E-32 |
| THBS2 | 4.429696 | 37.00495 | 3.062439 | 1.28E-27 | 8.81E-27 |
| RCOR2 | 0.275881 | 2.105193 | 2.931833 | 5.74E-20 | 1.89E-19 |
| ILF2 | 46.90137 | 107.907 | 1.202086 | 3.71E-30 | 3.76E-29 |
| RCN3 | 5.957754 | 21.85884 | 1.875376 | 4.49E-17 | 1.19E-16 |
| FAM111B | 0.457942 | 3.489784 | 2.9299 | 4.92E-31 | 6.03E-30 |
| CDH24 | 1.230606 | 3.939012 | 1.678465 | 2.77E-19 | 8.69E-19 |
| ACP6 | 0.927461 | 2.51943 | 1.441739 | 2.29E-27 | 1.52E-26 |
| THBD | 53.34013 | 10.79865 | -2.30437 | 1.11E-32 | 2.32E-31 |
| RCC2 | 20.25125 | 44.21905 | 1.126657 | 5.99E-29 | 5.05E-28 |
| CDH2 | 0.419067 | 1.841415 | 2.135562 | 0.01477 | 0.016862 |
| ACP5 | 130.5835 | 41.73248 | -1.64573 | 6.04E-29 | 5.08E-28 |
| RCC1 | 4.919104 | 18.05384 | 1.875838 | 1.80E-35 | 4.47E-33 |
| IL7R | 37.2444 | 10.26542 | -1.85923 | 9.88E-25 | 5.02E-24 |
| CDH1 | 35.97208 | 79.27525 | 1.139993 | 3.24E-21 | 1.19E-20 |
| ACHE | 0.85287 | 5.110214 | 2.582985 | 3.06E-08 | 4.84E-08 |
| CDCA8 | 0.936239 | 8.626213 | 3.203778 | 2.47E-34 | 1.91E-32 |
| ACBD7 | 0.326739 | 0.706511 | 1.112571 | 1.36E-05 | 1.86E-05 |
| FABP5 | 25.23207 | 7.736946 | -1.70542 | 7.06E-28 | 5.06E-27 |
| CDCA7 | 0.883535 | 7.403532 | 3.066854 | 9.12E-28 | 6.42E-27 |
| NETO2 | 1.335071 | 3.855475 | 1.529992 | 1.71E-17 | 4.67E-17 |
| FABP4 | 105.6455 | 4.052759 | -4.70418 | 1.35E-35 | 4.47E-33 |
| CDCA5 | 0.72151 | 6.358411 | 3.139576 | 9.18E-32 | 1.30E-30 |
| TGFBR2 | 116.1315 | 40.60868 | -1.5159 | 7.55E-34 | 3.22E-32 |
| RBMS3 | 4.771975 | 1.917918 | -1.31505 | 5.61E-27 | 3.55E-26 |
| IL4I1 | 1.801708 | 7.552526 | 2.067594 | 3.81E-17 | 1.02E-16 |
| CDCA4 | 2.833033 | 8.495795 | 1.584401 | 1.02E-27 | 7.08E-27 |
| CDCA3 | 0.221994 | 2.16831 | 3.287981 | 5.69E-34 | 2.70E-32 |
| ACADL | 6.679917 | 0.841702 | -2.98845 | 1.16E-34 | 1.20E-32 |
| NEK5 | 1.36372 | 0.524077 | -1.3797 | 2.21E-11 | 4.17E-11 |
| IL3RA | 19.35249 | 6.591672 | -1.5538 | 5.83E-32 | 8.76E-31 |
| FAAP24 | 0.877963 | 2.28235 | 1.378288 | 6.15E-25 | 3.20E-24 |
| CDCA2 | 0.193964 | 1.712981 | 3.142648 | 2.57E-32 | 4.50E-31 |
| ACAD8 | 3.208693 | 8.737918 | 1.445304 | 2.21E-20 | 7.51E-20 |
| IL37 | 0.138975 | 12.55855 | 6.497704 | 1.19E-13 | 2.59E-13 |
| CDC7 | 1.207461 | 3.285505 | 1.444139 | 1.42E-19 | 4.55E-19 |
| ACACB | 2.371783 | 1.053739 | -1.17045 | 2.05E-21 | 7.65E-21 |
| TFF2 | 0.016009 | 62.61504 | 11.93343 | 4.32E-07 | 6.42E-07 |
| NEK2 | 0.372782 | 5.481986 | 3.878296 | 1.13E-33 | 3.94E-32 |
| IL34 | 4.524821 | 2.06875 | -1.1291 | 2.11E-21 | 7.87E-21 |
| F8 | 5.945286 | 1.883587 | -1.65826 | 1.38E-26 | 8.41E-26 |
| CDC6 | 0.389468 | 4.621272 | 3.568715 | 5.97E-34 | 2.79E-32 |
| TFF1 | 0.167567 | 110.889 | 9.370166 | 8.29E-13 | 1.71E-12 |
| NEIL3 | 0.08507 | 1.801431 | 4.404354 | 3.50E-33 | 9.76E-32 |
| RBBP8NL | 0.437612 | 2.008949 | 2.198718 | 2.85E-25 | 1.52E-24 |
| F2RL3 | 5.775988 | 1.754005 | -1.71941 | 9.58E-13 | 1.97E-12 |
| CDC45 | 0.405433 | 4.054385 | 3.321947 | 1.71E-32 | 3.33E-31 |
| F2RL2 | 0.343701 | 0.943136 | 1.456313 | 0.000167 | 0.000215 |
| CDC25C | 0.113315 | 1.603509 | 3.822824 | 2.95E-34 | 1.91E-32 |
| ABI3BP | 13.91447 | 2.82123 | -2.30219 | 1.76E-32 | 3.40E-31 |
| IL2RA | 1.207109 | 3.618514 | 1.583842 | 2.09E-14 | 4.75E-14 |
| F12 | 0.263144 | 2.040627 | 2.955087 | 2.21E-26 | 1.33E-25 |
| CDC25A | 0.251153 | 1.427208 | 2.506554 | 1.65E-28 | 1.26E-27 |
| RASIP1 | 11.96009 | 2.672023 | -2.16222 | 3.15E-33 | 8.95E-32 |
| NDUFS6 | 21.28542 | 44.82857 | 1.074553 | 1.05E-22 | 4.38E-22 |
| CDC20 | 1.222837 | 18.55023 | 3.923133 | 7.19E-34 | 3.11E-32 |
| EZH2 | 0.781974 | 5.121606 | 2.711404 | 3.59E-34 | 2.00E-32 |
| NDUFAF6 | 1.510571 | 3.18281 | 1.075207 | 9.40E-21 | 3.29E-20 |
| CDA | 3.159459 | 18.55679 | 2.554198 | 2.99E-05 | 4.02E-05 |
| ABHD11 | 8.027712 | 23.49556 | 1.549327 | 9.24E-26 | 5.19E-25 |
| IL1RL1 | 9.333198 | 1.435899 | -2.70042 | 1.88E-24 | 9.26E-24 |
| ABCG2 | 3.891806 | 1.047589 | -1.89337 | 5.70E-30 | 5.55E-29 |
| CD93 | 58.44944 | 14.18553 | -2.04277 | 2.55E-32 | 4.48E-31 |
| TESMIN | 0.13798 | 1.005579 | 2.865495 | 2.12E-19 | 6.69E-19 |
| TESC | 3.482103 | 45.04009 | 3.693179 | 1.41E-09 | 2.38E-09 |
| IL18R1 | 3.344257 | 1.348028 | -1.31084 | 3.35E-14 | 7.51E-14 |
| NDC80 | 0.372238 | 3.644422 | 3.291393 | 1.20E-32 | 2.48E-31 |
| EXOSC5 | 5.861371 | 14.51602 | 1.308336 | 1.33E-25 | 7.30E-25 |
| ABCB9 | 0.28727 | 0.773419 | 1.428843 | 1.64E-24 | 8.14E-24 |
| TEKT2 | 6.079577 | 2.182762 | -1.47782 | 5.18E-08 | 8.08E-08 |
| TEK | 21.01197 | 2.463381 | -3.0925 | 3.22E-35 | 6.22E-33 |
| CD79A | 7.419555 | 22.8488 | 1.622714 | 2.82E-13 | 6.00E-13 |
| TEDC2 | 0.187499 | 2.366657 | 3.657899 | 9.31E-35 | 1.00E-32 |
| RANBP1 | 6.734712 | 14.16003 | 1.072136 | 1.00E-27 | 6.99E-27 |
| NCKAP1L | 10.89144 | 5.096609 | -1.09558 | 4.94E-22 | 1.93E-21 |
| EXO1 | 0.186794 | 2.540931 | 3.765836 | 7.93E-34 | 3.29E-32 |
| AARD | 3.294592 | 0.98256 | -1.74548 | 1.33E-25 | 7.30E-25 |
| RAMP3 | 80.53385 | 9.563153 | -3.07404 | 9.18E-34 | 3.45E-32 |
| NCF2 | 44.42653 | 15.56003 | -1.51358 | 2.97E-28 | 2.21E-27 |
| RAMP2 | 79.16902 | 11.77206 | -2.74957 | 5.62E-35 | 8.18E-33 |
| NCF1 | 3.478697 | 1.55954 | -1.15743 | 3.96E-16 | 1.00E-15 |
| CD70 | 0.333924 | 0.865164 | 1.373451 | 1.69E-05 | 2.31E-05 |
| RALGPS2 | 1.206699 | 4.248451 | 1.815871 | 3.63E-27 | 2.34E-26 |
| EVI2B | 28.22833 | 13.4597 | -1.0685 | 2.66E-21 | 9.78E-21 |
| NCAPH | 0.517333 | 4.651237 | 3.168449 | 4.51E-32 | 7.11E-31 |
| NCAPG2 | 1.260671 | 4.866678 | 1.948745 | 1.74E-30 | 1.91E-29 |
| ETV4 | 0.921363 | 14.53647 | 3.979764 | 1.28E-33 | 4.35E-32 |
| CD68 | 3.345529 | 1.215483 | -1.4607 | 3.20E-23 | 1.40E-22 |
| TDRD5 | 0.067968 | 0.93751 | 3.785912 | 7.62E-10 | 1.31E-09 |
| NCAPG | 0.325073 | 3.376134 | 3.376538 | 5.10E-32 | 7.88E-31 |
| NCAPD2 | 6.524154 | 14.58827 | 1.160947 | 1.72E-19 | 5.47E-19 |
| ETV1 | 9.359138 | 4.613196 | -1.02061 | 5.83E-21 | 2.08E-20 |
| RADX | 1.583549 | 0.549843 | -1.52607 | 2.93E-23 | 1.28E-22 |
| ETS1 | 33.07689 | 16.42863 | -1.00961 | 1.10E-20 | 3.84E-20 |
| RAD54L | 0.197583 | 2.158312 | 3.449373 | 3.00E-33 | 8.61E-32 |
| IGLL5 | 9.675903 | 59.90694 | 2.630255 | 6.56E-15 | 1.54E-14 |
| RAD54B | 0.159151 | 0.577866 | 1.860341 | 1.40E-28 | 1.09E-27 |
| ESCO2 | 0.104607 | 0.731436 | 2.805746 | 7.15E-31 | 8.36E-30 |
| RAD51AP1 | 0.894932 | 4.196483 | 2.229331 | 2.71E-26 | 1.61E-25 |
| ESAM | 57.0259 | 16.36884 | -1.80067 | 8.67E-32 | 1.25E-30 |
| CD52 | 206.8374 | 45.32978 | -2.18997 | 4.20E-31 | 5.25E-30 |
| NAXE | 29.47558 | 67.52569 | 1.195916 | 4.56E-24 | 2.17E-23 |
| TCF19 | 3.078062 | 7.711394 | 1.324969 | 7.87E-22 | 3.02E-21 |
| RACGAP1 | 2.759782 | 8.607962 | 1.641118 | 6.42E-24 | 2.97E-23 |
| ERICH5 | 1.281807 | 3.120875 | 1.283771 | 2.47E-05 | 3.34E-05 |
| RAC3 | 1.863929 | 10.1597 | 2.446439 | 7.45E-25 | 3.84E-24 |
| NAV1 | 1.444989 | 2.946708 | 1.028046 | 1.22E-11 | 2.33E-11 |
| IGFBP3 | 16.59999 | 81.76155 | 2.30024 | 4.54E-21 | 1.64E-20 |
| NATD1 | 7.040391 | 3.411481 | -1.04526 | 6.27E-24 | 2.91E-23 |
| IGF2BP3 | 0.099041 | 1.839618 | 4.215233 | 7.56E-14 | 1.66E-13 |
| ERG | 8.935946 | 2.252198 | -1.98829 | 5.10E-33 | 1.23E-31 |
| RAB6B | 1.41946 | 2.876882 | 1.019164 | 4.98E-05 | 6.62E-05 |
| ERCC6L | 0.108974 | 1.196596 | 3.456887 | 2.16E-35 | 4.47E-33 |
| IGF2BP1 | 0.010749 | 1.050507 | 6.61068 | 3.82E-12 | 7.56E-12 |
| TBX6 | 0.502178 | 1.179256 | 1.231605 | 2.71E-11 | 5.08E-11 |
| NARF | 3.771602 | 9.80841 | 1.378842 | 7.35E-33 | 1.68E-31 |
| ERBB2 | 16.55201 | 37.24483 | 1.170033 | 7.02E-16 | 1.75E-15 |
| TBX2 | 15.10661 | 4.411078 | -1.77598 | 9.19E-27 | 5.71E-26 |
| RAB3B | 0.038561 | 0.769874 | 4.319401 | 1.33E-17 | 3.68E-17 |
| TBRG4 | 5.070502 | 11.76652 | 1.214488 | 1.91E-30 | 2.08E-29 |
| EPYC | 0.017793 | 0.794683 | 5.481028 | 1.02E-15 | 2.51E-15 |
| RAB38 | 4.917325 | 10.28932 | 1.065202 | 2.43E-10 | 4.28E-10 |
| NAALADL2 | 0.590275 | 1.509319 | 1.354439 | 2.94E-10 | 5.17E-10 |
| IFNG | 0.329487 | 0.869432 | 1.399853 | 0.009089 | 0.010538 |
| CD36 | 19.92681 | 2.925336 | -2.76804 | 5.72E-33 | 1.34E-31 |
| EPOP | 0.763591 | 2.212069 | 1.534525 | 2.27E-18 | 6.64E-18 |
| CD34 | 15.84648 | 5.49338 | -1.5284 | 1.82E-28 | 1.39E-27 |
| RAB27B | 4.226172 | 8.482306 | 1.005105 | 0.001138 | 0.001392 |
| CD33 | 2.522869 | 1.039894 | -1.27863 | 5.59E-24 | 2.61E-23 |
| EPHX4 | 0.27154 | 1.825723 | 2.74923 | 4.70E-20 | 1.56E-19 |
| CD302 | 9.625972 | 4.2484 | -1.18001 | 7.86E-26 | 4.47E-25 |
| RAB15 | 4.974696 | 14.28652 | 1.521974 | 3.13E-15 | 7.52E-15 |
| CD300LF | 13.64144 | 4.480087 | -1.6064 | 1.35E-28 | 1.06E-27 |
| MZT2A | 2.730441 | 6.681447 | 1.291027 | 4.30E-26 | 2.51E-25 |
| EPHB3 | 2.773506 | 7.593186 | 1.452994 | 5.06E-15 | 1.20E-14 |
| RAB11FIP1 | 31.90745 | 12.99032 | -1.29646 | 3.84E-24 | 1.84E-23 |
| EPHB2 | 0.658924 | 3.504156 | 2.410884 | 3.42E-22 | 1.37E-21 |
| CD300E | 2.881826 | 1.239954 | -1.2167 | 7.33E-18 | 2.07E-17 |
| TBC1D31 | 0.516219 | 1.183647 | 1.197182 | 2.24E-18 | 6.56E-18 |
| MZB1 | 4.460958 | 21.3666 | 2.259932 | 8.80E-14 | 1.92E-13 |
| EPHA1 | 2.358697 | 4.788177 | 1.021486 | 2.65E-14 | 5.98E-14 |
| QPCT | 2.816222 | 19.57486 | 2.797169 | 9.16E-11 | 1.66E-10 |
| MYO7A | 0.432564 | 1.900273 | 2.135222 | 1.05E-29 | 9.81E-29 |
| CD27 | 2.834097 | 7.695283 | 1.441085 | 1.50E-11 | 2.86E-11 |
| CD244 | 1.764187 | 0.773247 | -1.19 | 1.64E-17 | 4.49E-17 |
| PYCR3 | 2.702142 | 7.720123 | 1.51452 | 5.56E-28 | 4.04E-27 |
| ID3 | 51.63196 | 25.17279 | -1.0364 | 6.07E-18 | 1.73E-17 |
| ENPP3 | 0.581309 | 2.882231 | 2.309809 | 0.022062 | 0.024826 |
| PYCR1 | 3.049044 | 43.9607 | 3.849785 | 2.07E-35 | 4.47E-33 |
| MYL9 | 171.8459 | 57.96609 | -1.56783 | 2.73E-32 | 4.65E-31 |
| PUS1 | 1.758524 | 4.37879 | 1.316167 | 4.14E-27 | 2.64E-26 |
| TAL1 | 3.021217 | 0.489482 | -2.6258 | 9.27E-34 | 3.45E-32 |
| ICAM2 | 8.860958 | 3.665395 | -1.27349 | 2.95E-27 | 1.93E-26 |
| ENG | 107.3439 | 38.43001 | -1.48194 | 4.41E-29 | 3.82E-28 |
| MYEOV | 0.070769 | 5.665579 | 6.322952 | 1.70E-27 | 1.14E-26 |
| ICA1 | 4.356053 | 10.78537 | 1.307982 | 6.67E-27 | 4.21E-26 |
| ENC1 | 7.052932 | 18.16386 | 1.364776 | 1.31E-19 | 4.21E-19 |
| PTRH2 | 2.439905 | 5.364446 | 1.136604 | 2.59E-30 | 2.70E-29 |
| MYCT1 | 11.91621 | 2.781852 | -2.09881 | 1.41E-33 | 4.69E-32 |
| IBSP | 0.266931 | 1.53291 | 2.521732 | 2.13E-18 | 6.25E-18 |
| MYCN | 0.148043 | 2.570822 | 4.118141 | 3.90E-10 | 6.82E-10 |
| MYCL | 1.782059 | 3.601147 | 1.014912 | 0.005583 | 0.006555 |
| HYLS1 | 0.896727 | 2.436972 | 1.442349 | 4.10E-30 | 4.11E-29 |
| EMP1 | 56.41509 | 18.73867 | -1.59006 | 1.44E-18 | 4.28E-18 |
| CD1A | 1.512657 | 5.007731 | 1.727073 | 0.002743 | 0.003279 |
| CD19 | 0.724399 | 1.930329 | 1.41399 | 8.28E-15 | 1.94E-14 |
| HYAL2 | 35.73734 | 16.04036 | -1.15573 | 2.37E-24 | 1.15E-23 |
| EME2 | 0.959178 | 2.173182 | 1.179939 | 5.43E-11 | 1.00E-10 |
| PTPRB | 16.28833 | 3.014602 | -2.4338 | 3.36E-32 | 5.57E-31 |
| EME1 | 0.193216 | 1.456147 | 2.913871 | 3.46E-32 | 5.70E-31 |
| MYBL2 | 1.303019 | 20.4471 | 3.971967 | 2.40E-31 | 3.14E-30 |
| EMCN | 15.40142 | 2.554057 | -2.5922 | 1.54E-33 | 4.90E-32 |
| MYBL1 | 0.475386 | 1.211594 | 1.349736 | 1.11E-11 | 2.14E-11 |
| TACC3 | 2.406433 | 8.446206 | 1.811407 | 1.01E-27 | 7.03E-27 |
| HTR2B | 0.439001 | 1.861874 | 2.084459 | 0.002268 | 0.002719 |
| TAC4 | 0.239383 | 1.926395 | 3.008514 | 7.15E-06 | 9.95E-06 |
| MXD3 | 0.571006 | 1.606716 | 1.492538 | 2.13E-20 | 7.26E-20 |
| CD101 | 6.650992 | 1.102193 | -2.59319 | 1.71E-32 | 3.33E-31 |
| TAC3 | 0.020109 | 0.804346 | 5.321936 | 0.002144 | 0.002574 |
| HSPD1 | 31.14984 | 83.49159 | 1.422406 | 7.94E-33 | 1.80E-31 |
| MX2 | 1.670704 | 5.179641 | 1.632396 | 4.05E-21 | 1.47E-20 |
| PTGIS | 12.22307 | 5.633459 | -1.11751 | 9.55E-18 | 2.68E-17 |
| HSPB7 | 3.181146 | 1.289009 | -1.30328 | 3.91E-22 | 1.55E-21 |
| CCT6A | 26.58498 | 63.17781 | 1.248807 | 1.99E-31 | 2.68E-30 |
| PTGIR | 3.389773 | 1.479187 | -1.19638 | 1.67E-21 | 6.28E-21 |
| MUC20 | 2.529849 | 13.68467 | 2.435437 | 1.19E-17 | 3.32E-17 |
| HSPB6 | 22.66479 | 4.540705 | -2.31946 | 5.06E-32 | 7.88E-31 |
| CCT5 | 16.55123 | 43.61816 | 1.397991 | 5.05E-29 | 4.32E-28 |
| SYT2 | 0.080211 | 0.653405 | 3.026101 | 1.58E-13 | 3.41E-13 |
| PTGFRN | 6.412558 | 15.8115 | 1.302003 | 6.37E-23 | 2.68E-22 |
| MTX1 | 3.391586 | 6.834189 | 1.01081 | 2.31E-28 | 1.75E-27 |
| CCT3 | 31.24448 | 87.14533 | 1.479822 | 2.29E-33 | 6.98E-32 |
| PTGER4 | 10.43943 | 3.915182 | -1.41489 | 5.99E-29 | 5.05E-28 |
| HSPA6 | 2.628795 | 7.114711 | 1.436403 | 4.81E-06 | 6.75E-06 |
| CCT2 | 19.7003 | 41.65363 | 1.080225 | 1.45E-18 | 4.30E-18 |
| PTGDS | 85.02874 | 29.19883 | -1.54204 | 1.49E-23 | 6.74E-23 |
| SYNGR3 | 0.17406 | 1.330911 | 2.93476 | 2.29E-26 | 1.38E-25 |
| MTHFD2 | 5.908016 | 18.69243 | 1.661708 | 3.75E-24 | 1.79E-23 |
| HSF4 | 1.114953 | 4.531847 | 2.023116 | 6.27E-15 | 1.48E-14 |
| SYNE4 | 2.722389 | 5.869444 | 1.108351 | 3.82E-13 | 8.07E-13 |
| MTFR2 | 0.554221 | 1.715875 | 1.630411 | 2.96E-16 | 7.53E-16 |
| PTCRA | 2.028495 | 0.423846 | -2.2588 | 6.27E-31 | 7.48E-30 |
| SYCP2 | 0.310191 | 1.281896 | 2.047049 | 0.000613 | 0.000762 |
| SYCE3 | 0.353699 | 1.386484 | 1.970836 | 2.06E-15 | 4.99E-15 |
| MTBP | 0.231612 | 0.844892 | 1.867058 | 3.60E-28 | 2.67E-27 |
| CCNO | 1.341185 | 6.271205 | 2.225234 | 1.91E-17 | 5.22E-17 |
| SYCE2 | 0.230705 | 0.59357 | 1.363372 | 2.07E-19 | 6.57E-19 |
| MSRB1 | 13.28314 | 29.61098 | 1.156535 | 1.49E-15 | 3.63E-15 |
| CCNI2 | 0.159167 | 0.589841 | 1.889782 | 4.99E-14 | 1.11E-13 |
| SYCE1L | 0.66445 | 1.591562 | 1.260212 | 5.61E-09 | 9.18E-09 |
| MSR1 | 37.22487 | 8.463701 | -2.13691 | 5.21E-31 | 6.32E-30 |
| CCNF | 0.809898 | 3.357331 | 2.051503 | 9.35E-32 | 1.32E-30 |
| PSRC1 | 1.126784 | 2.927586 | 1.3775 | 4.85E-18 | 1.39E-17 |
| CCNE2 | 0.302148 | 1.406972 | 2.219266 | 8.20E-29 | 6.64E-28 |
| MSLNL | 0.192869 | 1.612612 | 3.063709 | 0.011974 | 0.013773 |
| CCNE1 | 0.364593 | 4.021204 | 3.463269 | 5.15E-33 | 1.23E-31 |
| SVEP1 | 6.632569 | 1.764773 | -1.91009 | 5.71E-27 | 3.61E-26 |
| PSPH | 2.899552 | 7.46306 | 1.363937 | 1.22E-18 | 3.65E-18 |
| EGR3 | 6.017003 | 1.976588 | -1.60603 | 1.77E-11 | 3.36E-11 |
| CCND2 | 13.41914 | 6.695247 | -1.00308 | 2.48E-22 | 1.00E-21 |
| EGR2 | 13.46968 | 4.122062 | -1.70828 | 4.73E-20 | 1.57E-19 |
| PSMG3 | 7.047119 | 22.48013 | 1.673545 | 6.57E-31 | 7.78E-30 |
| MSH5 | 0.209408 | 0.771983 | 1.882255 | 2.63E-18 | 7.67E-18 |
| EGLN3 | 1.104242 | 9.751596 | 3.142582 | 2.02E-21 | 7.54E-21 |
| CCNB2 | 0.852725 | 7.72428 | 3.179248 | 3.47E-33 | 9.76E-32 |
| MSC | 1.960152 | 3.959863 | 1.014485 | 3.89E-07 | 5.80E-07 |
| HPGDS | 5.200483 | 2.242681 | -1.21342 | 9.40E-20 | 3.04E-19 |
| CCNB1 | 2.007287 | 17.27492 | 3.10536 | 3.16E-34 | 1.91E-32 |
| SULT1C4 | 3.58618 | 0.820087 | -2.1286 | 8.61E-30 | 8.13E-29 |
| CCNA2 | 0.970357 | 7.966555 | 3.037368 | 1.99E-32 | 3.70E-31 |
| SULT1C2 | 0.772082 | 2.776944 | 1.846672 | 0.000118 | 0.000154 |
| MS4A7 | 32.7882 | 8.466595 | -1.95332 | 3.52E-32 | 5.77E-31 |
| HPDL | 0.110449 | 1.373832 | 3.636759 | 1.08E-26 | 6.69E-26 |
| CCNA1 | 1.289191 | 0.593216 | -1.11984 | 1.59E-08 | 2.55E-08 |
| SULF1 | 3.255376 | 20.78502 | 2.674648 | 2.14E-23 | 9.45E-23 |
| EFNA5 | 2.381649 | 7.022284 | 1.559979 | 1.97E-17 | 5.37E-17 |
| CCN4 | 0.731277 | 3.900706 | 2.415245 | 3.57E-24 | 1.71E-23 |
| MS4A4A | 20.0405 | 9.8156 | -1.02977 | 4.36E-19 | 1.35E-18 |
| EFNA4 | 3.400926 | 16.87627 | 2.310997 | 1.52E-33 | 4.90E-32 |
| CCN2 | 235.774 | 85.93369 | -1.45611 | 2.96E-20 | 9.97E-20 |
| STXBP6 | 6.39154 | 0.686499 | -3.21883 | 5.52E-34 | 2.70E-32 |
| HOXC13 | 0.006079 | 0.974584 | 7.324928 | 3.97E-14 | 8.84E-14 |
| PSMB4 | 58.61094 | 118.3843 | 1.014236 | 7.27E-27 | 4.58E-26 |
| MS4A2 | 3.822198 | 1.18256 | -1.69249 | 5.88E-23 | 2.49E-22 |
| CCL7 | 0.324146 | 1.346088 | 2.054057 | 9.28E-11 | 1.68E-10 |
| STX1A | 0.572127 | 5.20107 | 3.1844 | 4.72E-34 | 2.40E-32 |
| MS4A1 | 1.532164 | 3.312523 | 1.11236 | 1.83E-07 | 2.77E-07 |
| HOXB9 | 0.031933 | 5.508557 | 7.430478 | 6.18E-14 | 1.36E-13 |
| EFEMP1 | 83.14888 | 30.28907 | -1.4569 | 2.66E-29 | 2.34E-28 |
| EFCAB13 | 0.273403 | 0.732263 | 1.421331 | 1.97E-11 | 3.73E-11 |
| HOXB13 | 0.008342 | 0.877495 | 6.716855 | 4.68E-10 | 8.15E-10 |
| EDN1 | 47.06019 | 13.16776 | -1.8375 | 2.09E-22 | 8.48E-22 |
| PSAT1 | 1.120041 | 13.92935 | 3.636505 | 1.27E-30 | 1.45E-29 |
| HOXA4 | 1.811456 | 0.702512 | -1.36656 | 2.39E-24 | 1.16E-23 |
| EDARADD | 0.353703 | 1.128822 | 1.674207 | 0.020489 | 0.02311 |
| CCL26 | 0.324017 | 0.857481 | 1.404034 | 4.81E-05 | 6.41E-05 |
| STRA6 | 0.094309 | 2.302314 | 4.609549 | 2.35E-26 | 1.41E-25 |
| CCL23 | 8.195078 | 1.57977 | -2.37504 | 1.51E-30 | 1.68E-29 |
| PRX | 21.47751 | 2.273021 | -3.24014 | 1.28E-32 | 2.62E-31 |
| CCL22 | 2.670244 | 5.662496 | 1.084466 | 0.000922 | 0.001136 |
| HOXA1 | 0.186444 | 0.84368 | 2.177952 | 0.000293 | 0.000373 |
| ECT2 | 2.0777 | 10.43946 | 2.328988 | 1.38E-32 | 2.79E-31 |
| MRPL24 | 17.04964 | 39.0902 | 1.197066 | 1.67E-27 | 1.13E-26 |
| HORMAD1 | 0.118584 | 2.788986 | 4.555759 | 2.79E-06 | 3.96E-06 |
| ECSCR | 13.31998 | 2.81948 | -2.24009 | 6.20E-35 | 8.18E-33 |
| CCL20 | 10.7287 | 29.75677 | 1.471743 | 1.95E-05 | 2.65E-05 |
| ECE2 | 0.445744 | 2.085256 | 2.225938 | 2.29E-31 | 3.03E-30 |
| CCL2 | 59.71354 | 23.98548 | -1.3159 | 8.87E-07 | 1.30E-06 |
| STMN1 | 11.5508 | 26.15701 | 1.179204 | 1.90E-10 | 3.39E-10 |
| CCL19 | 13.7824 | 32.76171 | 1.249183 | 3.25E-07 | 4.85E-07 |
| STK32A | 0.707849 | 3.304616 | 2.222969 | 9.05E-19 | 2.73E-18 |
| PRRX2 | 0.902867 | 4.570976 | 2.339917 | 1.28E-10 | 2.32E-10 |
| MRGBP | 3.983498 | 9.543635 | 1.260503 | 2.05E-32 | 3.78E-31 |
| CCL18 | 169.8054 | 82.63113 | -1.03912 | 4.35E-06 | 6.12E-06 |
| STK31 | 0.228076 | 1.054391 | 2.208821 | 8.86E-19 | 2.68E-18 |
| PRR7 | 0.608963 | 1.769988 | 1.539314 | 1.63E-16 | 4.20E-16 |
| MRC1 | 84.92547 | 19.30227 | -2.13743 | 1.79E-30 | 1.96E-29 |
| CCL14 | 2.128807 | 0.441847 | -2.26843 | 1.80E-24 | 8.94E-24 |
| STIL | 0.30277 | 2.008665 | 2.729942 | 2.62E-34 | 1.91E-32 |
| E2F7 | 0.107351 | 0.792208 | 2.883541 | 1.83E-25 | 9.92E-25 |
| STEAP1 | 2.050827 | 15.83666 | 2.94899 | 1.44E-23 | 6.54E-23 |
| PRR19 | 0.174877 | 0.878649 | 2.328946 | 7.84E-29 | 6.41E-28 |
| E2F5 | 1.15893 | 3.060339 | 1.400898 | 4.56E-25 | 2.41E-24 |
| MPP6 | 0.717271 | 1.769332 | 1.302614 | 4.38E-16 | 1.10E-15 |
| HMMR | 0.455562 | 4.149489 | 3.187214 | 9.99E-33 | 2.13E-31 |
| E2F3 | 2.160079 | 6.286508 | 1.541175 | 5.35E-31 | 6.47E-30 |
| PRR11 | 0.886873 | 4.658431 | 2.393045 | 1.42E-24 | 7.17E-24 |
| HMGB3 | 6.458027 | 79.15708 | 3.615553 | 5.72E-32 | 8.72E-31 |
| E2F1 | 3.16123 | 9.782405 | 1.629703 | 1.38E-17 | 3.81E-17 |
| HMGB2 | 16.45552 | 33.21375 | 1.013209 | 6.00E-15 | 1.42E-14 |
| HMGA1 | 22.9883 | 122.422 | 2.412891 | 2.54E-30 | 2.66E-29 |
| CCDC34 | 1.254432 | 3.672802 | 1.549847 | 3.98E-23 | 1.71E-22 |
| CCDC28B | 1.05798 | 2.156266 | 1.027222 | 6.08E-15 | 1.43E-14 |
| STAG3 | 0.364148 | 0.945809 | 1.377025 | 1.49E-05 | 2.03E-05 |
| MOCOS | 1.552642 | 3.198321 | 1.042589 | 5.98E-11 | 1.10E-10 |
| STAC | 6.70715 | 1.762353 | -1.9282 | 7.23E-29 | 5.99E-28 |
| CCDC18 | 0.488264 | 0.979497 | 1.004379 | 5.16E-10 | 8.95E-10 |
| MNDA | 23.72042 | 9.614225 | -1.30289 | 7.70E-25 | 3.96E-24 |
| DUSP23 | 30.72795 | 67.80977 | 1.141942 | 1.40E-14 | 3.21E-14 |
| PRKCQ | 4.268511 | 1.602126 | -1.41374 | 3.24E-27 | 2.10E-26 |
| MND1 | 0.284334 | 2.381988 | 3.06651 | 6.46E-34 | 2.93E-32 |
| CCDC167 | 9.757994 | 28.18765 | 1.530406 | 1.58E-26 | 9.59E-26 |
| MMS22L | 0.315208 | 0.640329 | 1.02251 | 1.92E-14 | 4.37E-14 |
| CCDC154 | 0.179256 | 1.081572 | 2.593038 | 1.85E-11 | 3.50E-11 |
| MMRN2 | 17.66402 | 4.446319 | -1.99013 | 1.15E-33 | 3.96E-32 |
| CCDC151 | 2.002117 | 0.862469 | -1.21498 | 1.93E-05 | 2.63E-05 |
| MMRN1 | 8.019148 | 1.776623 | -2.17431 | 9.72E-26 | 5.43E-25 |
| MMP9 | 8.445666 | 37.58517 | 2.15388 | 7.65E-16 | 1.90E-15 |
| DUS4L | 0.819851 | 1.960072 | 1.257474 | 5.20E-30 | 5.13E-29 |
| ST6GALNAC2 | 5.865431 | 2.239954 | -1.38877 | 5.25E-25 | 2.76E-24 |
| PRIM1 | 1.99914 | 4.064334 | 1.02364 | 1.62E-12 | 3.28E-12 |
| DUS1L | 7.625982 | 19.20223 | 1.332279 | 6.66E-30 | 6.36E-29 |
| DTYMK | 5.18755 | 13.7544 | 1.406768 | 2.77E-27 | 1.82E-26 |
| PRF1 | 15.13223 | 6.87434 | -1.13833 | 6.67E-16 | 1.66E-15 |
| HK3 | 15.3666 | 5.025093 | -1.61258 | 1.27E-25 | 7.02E-25 |
| CBX8 | 1.182905 | 3.270773 | 1.467297 | 1.43E-30 | 1.59E-29 |
| PRELP | 39.24863 | 14.70395 | -1.41644 | 2.06E-21 | 7.70E-21 |
| MMP15 | 10.77702 | 23.87824 | 1.147737 | 5.03E-13 | 1.05E-12 |
| HJURP | 0.277425 | 4.523958 | 4.027414 | 2.33E-34 | 1.88E-32 |
| PRELID3A | 0.488322 | 1.062496 | 1.121552 | 4.71E-16 | 1.18E-15 |
| MMP12 | 1.174265 | 20.82349 | 4.148381 | 4.56E-23 | 1.95E-22 |
| DTL | 0.89911 | 3.603516 | 2.002835 | 4.91E-24 | 2.31E-23 |
| CBX3 | 20.96463 | 47.95919 | 1.19385 | 8.67E-32 | 1.25E-30 |
| CBX2 | 0.580479 | 3.526946 | 2.603103 | 4.71E-21 | 1.69E-20 |
| MME | 10.51169 | 2.066744 | -2.34656 | 4.10E-32 | 6.50E-31 |
| DSN1 | 4.057653 | 9.208107 | 1.182259 | 2.25E-24 | 1.10E-23 |
| DSCC1 | 0.841499 | 3.193618 | 1.924159 | 1.66E-23 | 7.44E-23 |
| PRC1 | 1.01468 | 6.471587 | 2.673094 | 1.63E-33 | 5.08E-32 |
| PRAP1 | 0.093759 | 2.146258 | 4.516723 | 0.000401 | 0.000506 |
| SRXN1 | 0.594265 | 1.708452 | 1.523512 | 1.01E-05 | 1.39E-05 |
| PRAME | 0.033246 | 7.252999 | 7.769253 | 4.78E-22 | 1.87E-21 |
| MKI67 | 0.685663 | 6.618968 | 3.271034 | 3.52E-31 | 4.48E-30 |
| CAVIN2 | 118.8121 | 13.33488 | -3.15541 | 9.89E-36 | 4.47E-33 |
| MITF | 4.462575 | 2.222873 | -1.00545 | 3.16E-25 | 1.68E-24 |
| CAV1 | 344.7297 | 34.83055 | -3.30704 | 1.09E-35 | 4.47E-33 |
| MIS18A | 3.060088 | 8.039089 | 1.393459 | 4.79E-31 | 5.88E-30 |
| DPY19L1 | 8.160947 | 19.82448 | 1.280474 | 9.42E-18 | 2.65E-17 |
| CAT | 100.3874 | 29.61703 | -1.76108 | 1.95E-35 | 4.47E-33 |
| SRSF12 | 0.111582 | 0.549999 | 2.301327 | 2.88E-17 | 7.75E-17 |
| DPT | 35.2347 | 13.89835 | -1.34208 | 1.94E-16 | 4.98E-16 |
| SRRM3 | 0.128285 | 0.698609 | 2.445131 | 6.22E-23 | 2.63E-22 |
| PPP1R35 | 6.210993 | 16.47122 | 1.407051 | 7.41E-26 | 4.23E-25 |
| MIF | 17.57731 | 44.02822 | 1.324714 | 1.23E-20 | 4.28E-20 |
| DPP4 | 11.04562 | 29.46024 | 1.415294 | 0.0006 | 0.000748 |

**Supplementary Table 3:** There were 203 prognostic TME-related genes were obtained by univariate Cox analysis in LUAD.

| gene | HR | HR.95L | HR.95H | pvalue |
| --- | --- | --- | --- | --- |
| SRPK1 | 1.3735 | 1.052362 | 1.792635 | 0.019514 |
| MFAP4 | 0.855691 | 0.772807 | 0.947463 | 0.002716 |
| SPOCK2 | 0.865041 | 0.752773 | 0.994052 | 0.040946 |
| SPOCK1 | 1.196261 | 1.074366 | 1.331987 | 0.001083 |
| PPAT | 1.323838 | 1.066351 | 1.643499 | 0.011019 |
| SPIB | 0.762667 | 0.619799 | 0.938468 | 0.010466 |
| PPARG | 1.153329 | 1.002119 | 1.327355 | 0.046647 |
| MELK | 1.216193 | 1.080061 | 1.369482 | 0.001231 |
| CA9 | 1.079436 | 1.00293 | 1.161778 | 0.041553 |
| CA4 | 0.763584 | 0.630098 | 0.925349 | 0.005935 |
| SOX9 | 1.109216 | 1.001007 | 1.229122 | 0.047795 |
| MCM7 | 1.189651 | 1.003441 | 1.410417 | 0.045553 |
| DLGAP5 | 1.336018 | 1.173018 | 1.521669 | 1.28E-05 |
| MCM6 | 1.304853 | 1.083928 | 1.570806 | 0.004931 |
| MCM4 | 1.295664 | 1.110521 | 1.511673 | 0.000993 |
| MCM2 | 1.207929 | 1.037212 | 1.406744 | 0.015101 |
| MC1R | 1.228544 | 1.016748 | 1.484459 | 0.033006 |
| HAL | 1.173859 | 1.050094 | 1.312212 | 0.004805 |
| SNRPE | 1.434807 | 1.103246 | 1.866012 | 0.007085 |
| SNRPA1 | 1.374351 | 1.073368 | 1.759734 | 0.01169 |
| PLK4 | 1.29921 | 1.081183 | 1.561202 | 0.005226 |
| PLK1 | 1.436513 | 1.234352 | 1.671783 | 2.86E-06 |
| MAOB | 0.806627 | 0.702981 | 0.925554 | 0.002195 |
| MAL | 0.789749 | 0.672561 | 0.927356 | 0.003974 |
| XRCC2 | 1.146125 | 1.006184 | 1.305529 | 0.040096 |
| MAD2L1 | 1.224385 | 1.091059 | 1.374004 | 0.000578 |
| BRCA2 | 1.259572 | 1.018378 | 1.557891 | 0.033347 |
| BRCA1 | 1.245211 | 1.041011 | 1.489466 | 0.016405 |
| SLC7A5 | 1.179263 | 1.046732 | 1.328575 | 0.006711 |
| WNT7A | 1.200394 | 1.054859 | 1.366008 | 0.005608 |
| PKMYT1 | 1.368144 | 1.137651 | 1.645336 | 0.000868 |
| PKM | 1.731818 | 1.328536 | 2.257516 | 4.90E-05 |
| GPR18 | 0.684836 | 0.502696 | 0.93297 | 0.016405 |
| BLM | 1.249875 | 1.083857 | 1.441321 | 0.002159 |
| PIM2 | 0.86306 | 0.77049 | 0.966752 | 0.010957 |
| WASF1 | 1.190885 | 1.017159 | 1.394282 | 0.029898 |
| LST1 | 0.870797 | 0.776397 | 0.976676 | 0.018123 |
| SLC2A1 | 1.284995 | 1.15004 | 1.435787 | 9.45E-06 |
| CYP27A1 | 0.781733 | 0.67538 | 0.904833 | 0.000966 |
| BIK | 1.178275 | 1.021889 | 1.358594 | 0.023946 |
| GPM6B | 0.822868 | 0.695781 | 0.973168 | 0.022743 |
| GPI | 1.485331 | 1.195839 | 1.844904 | 0.000348 |
| GPC3 | 0.872646 | 0.788378 | 0.965921 | 0.008559 |
| PFKP | 1.248317 | 1.112656 | 1.400519 | 0.000158 |
| LPGAT1 | 1.456356 | 1.162644 | 1.824268 | 0.001071 |
| CTSG | 0.726834 | 0.583858 | 0.904821 | 0.004305 |
| VAMP2 | 0.791017 | 0.64889 | 0.964276 | 0.020343 |
| SLC15A2 | 0.785366 | 0.674895 | 0.913919 | 0.001786 |
| B4GALNT1 | 1.223383 | 1.029701 | 1.453496 | 0.02186 |
| PECAM1 | 0.798588 | 0.674768 | 0.945129 | 0.008884 |
| LMNB2 | 1.36735 | 1.127782 | 1.657809 | 0.001455 |
| LMNB1 | 1.276194 | 1.091369 | 1.492319 | 0.002248 |
| CTLA4 | 0.779734 | 0.633793 | 0.95928 | 0.018615 |
| PDX1 | 1.195338 | 1.04813 | 1.36322 | 0.00779 |
| GINS1 | 1.167967 | 1.009353 | 1.351507 | 0.037072 |
| UPK2 | 1.167011 | 1.002765 | 1.358158 | 0.045972 |
| GGH | 1.143577 | 1.030355 | 1.26924 | 0.011665 |
| SIX1 | 0.863628 | 0.763719 | 0.976606 | 0.019421 |
| GDF15 | 0.872653 | 0.793306 | 0.959937 | 0.005101 |
| UBE2S | 1.29132 | 1.122997 | 1.484872 | 0.000333 |
| ATIC | 1.552721 | 1.160281 | 2.077895 | 0.003076 |
| UBE2C | 1.171277 | 1.060404 | 1.293742 | 0.001834 |
| LDHA | 1.788229 | 1.441504 | 2.218351 | 1.26E-07 |
| GATA2 | 0.781639 | 0.633503 | 0.964416 | 0.021566 |
| TYMS | 1.307775 | 1.133586 | 1.508731 | 0.000234 |
| GAPDH | 1.597159 | 1.324957 | 1.925283 | 9.04E-07 |
| ASPH | 1.207579 | 1.076233 | 1.354954 | 0.001325 |
| TXNRD1 | 1.152495 | 1.044464 | 1.2717 | 0.004709 |
| TTK | 1.242692 | 1.06689 | 1.447463 | 0.005239 |
| CPA3 | 0.856327 | 0.774219 | 0.947144 | 0.002562 |
| TSPAN7 | 0.869515 | 0.784175 | 0.964144 | 0.007984 |
| KPNA2 | 1.361325 | 1.1647 | 1.591146 | 0.000106 |
| SEMA3A | 1.126228 | 1.026262 | 1.235932 | 0.012191 |
| PAFAH1B3 | 1.15246 | 1.009752 | 1.315337 | 0.035392 |
| SELP | 0.784263 | 0.650198 | 0.945972 | 0.011064 |
| COL9A2 | 0.875705 | 0.771844 | 0.993541 | 0.039344 |
| TROAP | 1.121607 | 1.023299 | 1.229359 | 0.014203 |
| FURIN | 1.227379 | 1.091252 | 1.380487 | 0.000636 |
| ARHGAP11A | 1.323186 | 1.162279 | 1.506368 | 2.30E-05 |
| PABPC1 | 1.331024 | 1.06346 | 1.665908 | 0.012514 |
| TRIP13 | 1.180581 | 1.030616 | 1.352367 | 0.016618 |
| P4HA1 | 1.323417 | 1.096335 | 1.597533 | 0.003528 |
| COL5A2 | 1.139865 | 1.018067 | 1.276233 | 0.023175 |
| COL4A4 | 0.844342 | 0.742381 | 0.960306 | 0.009972 |
| P2RY14 | 0.745149 | 0.605401 | 0.917157 | 0.005503 |
| FSCN1 | 1.286063 | 1.146587 | 1.442505 | 1.74E-05 |
| P2RX1 | 0.607423 | 0.458497 | 0.804723 | 0.000513 |
| COL1A2 | 1.110938 | 1.001326 | 1.232548 | 0.047148 |
| COL1A1 | 1.109394 | 1.007616 | 1.221453 | 0.034473 |
| ORC1 | 1.249255 | 1.083936 | 1.439788 | 0.00212 |
| FOXM1 | 1.215394 | 1.105691 | 1.335982 | 5.31E-05 |
| KIFC1 | 1.177558 | 1.055361 | 1.313905 | 0.003457 |
| KIF2C | 1.229889 | 1.084841 | 1.394331 | 0.00123 |
| S100B | 0.907178 | 0.827515 | 0.99451 | 0.037768 |
| TPBG | 1.270122 | 1.064397 | 1.515609 | 0.007997 |
| KIF23 | 1.328045 | 1.13798 | 1.549856 | 0.000318 |
| FLI1 | 0.796072 | 0.65118 | 0.973204 | 0.026085 |
| KIF14 | 1.388124 | 1.183055 | 1.628739 | 5.79E-05 |
| OAS1 | 1.189114 | 1.038818 | 1.361155 | 0.011993 |
| TOP2A | 1.174275 | 1.04839 | 1.315276 | 0.005491 |
| FKBP4 | 1.56083 | 1.296754 | 1.878684 | 2.50E-06 |
| KIF11 | 1.331078 | 1.139327 | 1.555102 | 0.000314 |
| FHL2 | 1.144456 | 1.009832 | 1.297027 | 0.034585 |
| FGR | 0.825812 | 0.697002 | 0.978428 | 0.026965 |
| ALPL | 0.919842 | 0.846435 | 0.999614 | 0.048947 |
| RRM2 | 1.299595 | 1.156079 | 1.460928 | 1.14E-05 |
| NUDT1 | 1.268721 | 1.072034 | 1.501494 | 0.005618 |
| ALOX15 | 0.867479 | 0.761088 | 0.988742 | 0.033208 |
| CKS2 | 1.172369 | 1.018115 | 1.349994 | 0.027147 |
| CKS1B | 1.333344 | 1.142428 | 1.556165 | 0.000263 |
| TNFRSF17 | 0.828322 | 0.724336 | 0.947237 | 0.005924 |
| CKAP4 | 1.461185 | 1.180305 | 1.808907 | 0.000498 |
| ALDOA | 1.475658 | 1.195267 | 1.821826 | 0.000296 |
| KCNJ15 | 0.878074 | 0.790181 | 0.975744 | 0.01568 |
| FCN1 | 0.827097 | 0.716067 | 0.955344 | 0.009848 |
| CHML | 1.156398 | 1.032483 | 1.295184 | 0.01198 |
| CHAF1B | 1.211781 | 1.064395 | 1.379574 | 0.003694 |
| CHAF1A | 1.269081 | 1.017016 | 1.583621 | 0.034917 |
| CHAD | 0.846269 | 0.729397 | 0.981867 | 0.027715 |
| CFTR | 0.761673 | 0.650866 | 0.891344 | 0.000689 |
| AFF3 | 0.844212 | 0.718448 | 0.991992 | 0.039623 |
| CFP | 0.807711 | 0.669428 | 0.97456 | 0.025818 |
| ADRB2 | 0.700808 | 0.566583 | 0.866832 | 0.001048 |
| ITGB4 | 1.156185 | 1.05448 | 1.267699 | 0.002007 |
| ITGAL | 0.780856 | 0.662891 | 0.919813 | 0.003074 |
| CENPI | 1.298483 | 1.038896 | 1.622932 | 0.021717 |
| CENPF | 1.254307 | 1.097009 | 1.434159 | 0.000919 |
| CENPE | 1.317109 | 1.120166 | 1.548678 | 0.000859 |
| CEL | 0.789232 | 0.633081 | 0.983898 | 0.035354 |
| IRF4 | 0.810803 | 0.702911 | 0.935257 | 0.003993 |
| TK1 | 1.311132 | 1.151471 | 1.492931 | 4.34E-05 |
| RFC4 | 1.209529 | 1.03992 | 1.4068 | 0.013596 |
| RFC3 | 1.212187 | 1.009346 | 1.45579 | 0.039443 |
| CDKN3 | 1.323028 | 1.156212 | 1.513911 | 4.68E-05 |
| CDK5R1 | 1.227797 | 1.092516 | 1.37983 | 0.00057 |
| CDK1 | 1.221964 | 1.099327 | 1.358282 | 0.000203 |
| ACP5 | 0.851813 | 0.729442 | 0.994713 | 0.042667 |
| IL7R | 0.849088 | 0.743206 | 0.970055 | 0.016068 |
| IL3RA | 0.804026 | 0.65564 | 0.985995 | 0.03613 |
| NEK2 | 1.313097 | 1.147328 | 1.502817 | 7.62E-05 |
| F8 | 0.765098 | 0.590809 | 0.990803 | 0.042355 |
| CDC6 | 1.189719 | 1.069978 | 1.322861 | 0.001329 |
| TFF1 | 1.057753 | 1.010062 | 1.107697 | 0.017066 |
| CDC25C | 1.375878 | 1.181978 | 1.601585 | 3.84E-05 |
| F12 | 1.267075 | 1.076674 | 1.491146 | 0.004383 |
| CDC25A | 1.337532 | 1.115505 | 1.60375 | 0.001688 |
| CDC20 | 1.215703 | 1.086636 | 1.360101 | 0.000647 |
| CDA | 1.104774 | 1.024913 | 1.190858 | 0.009248 |
| TEK | 0.838565 | 0.725724 | 0.968951 | 0.016953 |
| CD79A | 0.85743 | 0.778549 | 0.944304 | 0.001785 |
| RANBP1 | 1.2401 | 1.048553 | 1.466639 | 0.011944 |
| NCKAP1L | 0.816867 | 0.687073 | 0.971181 | 0.021953 |
| EVI2B | 0.842681 | 0.729929 | 0.97285 | 0.019515 |
| NCAPH | 1.244312 | 1.085311 | 1.426607 | 0.001727 |
| NCAPD2 | 1.298338 | 1.112661 | 1.514999 | 0.000914 |
| ETV1 | 0.820998 | 0.700452 | 0.962289 | 0.014915 |
| CD52 | 0.841049 | 0.744669 | 0.949904 | 0.00531 |
| IGFBP3 | 1.134628 | 1.016829 | 1.266075 | 0.023923 |
| IGF2BP3 | 1.223991 | 1.089633 | 1.374917 | 0.000657 |
| ERG | 0.753013 | 0.611801 | 0.926817 | 0.007424 |
| RAB3B | 1.219219 | 1.019741 | 1.457718 | 0.029673 |
| RAB27B | 1.208766 | 1.062161 | 1.375607 | 0.004051 |
| CD33 | 0.698439 | 0.532562 | 0.915982 | 0.009478 |
| EPHB2 | 1.172614 | 1.035952 | 1.327305 | 0.011781 |
| TBC1D31 | 1.347213 | 1.072046 | 1.693008 | 0.010565 |
| CD27 | 0.846504 | 0.742604 | 0.96494 | 0.012627 |
| MYCL | 0.818296 | 0.675122 | 0.991835 | 0.041002 |
| CD1A | 0.906606 | 0.823392 | 0.998229 | 0.045929 |
| CD19 | 0.756687 | 0.629848 | 0.90907 | 0.002898 |
| MYBL2 | 1.146419 | 1.041506 | 1.2619 | 0.005263 |
| CD101 | 0.755453 | 0.621086 | 0.918888 | 0.005008 |
| HSPD1 | 1.451717 | 1.165766 | 1.807808 | 0.000867 |
| CCT6A | 1.475032 | 1.236501 | 1.759578 | 1.57E-05 |
| CCT5 | 1.293787 | 1.061621 | 1.576725 | 0.010694 |
| CCT2 | 1.138415 | 1.026876 | 1.262069 | 0.013737 |
| PTGDS | 0.839898 | 0.754876 | 0.934498 | 0.001355 |
| MTHFD2 | 1.231271 | 1.04918 | 1.444964 | 0.010837 |
| PTCRA | 0.650906 | 0.439705 | 0.963551 | 0.031914 |
| CCNF | 1.23749 | 1.008411 | 1.518608 | 0.041335 |
| CCNE1 | 1.206756 | 1.054493 | 1.381005 | 0.006314 |
| PSPH | 1.204149 | 1.036241 | 1.399263 | 0.015326 |
| CCND2 | 0.8222 | 0.689458 | 0.980499 | 0.029318 |
| EGR2 | 0.866497 | 0.757153 | 0.991632 | 0.037337 |
| CCNB1 | 1.29907 | 1.153127 | 1.463484 | 1.68E-05 |
| CCNA2 | 1.302986 | 1.150536 | 1.475636 | 3.06E-05 |
| EFNA5 | 1.197958 | 1.039228 | 1.380933 | 0.012755 |
| MS4A2 | 0.756521 | 0.642393 | 0.890925 | 0.000825 |
| MS4A1 | 0.818131 | 0.731369 | 0.915186 | 0.000449 |
| HOXA1 | 1.173731 | 1.038155 | 1.327012 | 0.010531 |
| CCL20 | 1.111467 | 1.033118 | 1.195759 | 0.004604 |
| MRC1 | 0.886547 | 0.800331 | 0.98205 | 0.021057 |
| STIL | 1.227307 | 1.019619 | 1.477299 | 0.030359 |
| HMMR | 1.431687 | 1.226644 | 1.671003 | 5.36E-06 |
| HMGA1 | 1.311054 | 1.156258 | 1.486574 | 2.39E-05 |
| MNDA | 0.850774 | 0.744131 | 0.972701 | 0.018029 |
| PRIM1 | 1.193688 | 1.03495 | 1.376771 | 0.015023 |
| DTYMK | 1.384497 | 1.130395 | 1.695718 | 0.001663 |
| PRELP | 0.89481 | 0.816091 | 0.981123 | 0.018002 |
| CBX3 | 1.343642 | 1.054952 | 1.711333 | 0.016692 |
| MKI67 | 1.216231 | 1.098038 | 1.347148 | 0.000175 |
| CAT | 0.715882 | 0.587513 | 0.872301 | 0.000917 |
| MIF | 1.305522 | 1.096393 | 1.554541 | 0.002761 |
| DPP4 | 0.908987 | 0.837944 | 0.986054 | 0.02155 |

**Supplementary Table 4:** There were 47 genes related to checkpoint blockade.

| IDO1 | CD276 | CD44 |
| --- | --- | --- |
| LAG3 | CD40 | TNFSF18 |
| CTLA4 | TNFRSF4 | TNFRSF18 |
| TNFRSF9 | TNFSF14 | BTNL2 |
| ICOS | HHLA2 | C10orf54 |
| CD80 | CD244 | CD200R1 |
| PDCD1LG2 | CD274 | TNFSF4 |
| TIGIT | HAVCR2 | CD200 |
| CD70 | CD27 | NRP1 |
| TNFSF9 | BTLA | CD48 |
| ICOSLG | LGALS9 | TNFRSF25 |
| KIR3DL1 | TMIGD2 | CD40LG |
| CD86 | CD28 | ADORA2A |
| CD160 | PDCD1 | VTCN1 |
| LAIR1 | TNFRSF8 | TNFSF15 |
| TNFRSF14 | IDO2 |  |
